# Trans-Atlantic Distribution and Introgression as Inferred from Single Nucleotide Polymorphism: Mussels *Mytilus* and Environmental Factors

**DOI:** 10.3390/genes11050530

**Published:** 2020-05-10

**Authors:** Roman Wenne, Małgorzata Zbawicka, Lis Bach, Petr Strelkov, Mikhail Gantsevich, Piotr Kukliński, Tomasz Kijewski, John H. McDonald, Kristil Kindem Sundsaasen, Mariann Árnyasi, Sigbjørn Lien, Ants Kaasik, Kristjan Herkül, Jonne Kotta

**Affiliations:** 1 Institute of Oceanology, Polish Academy of Sciences, 81-712 Sopot, Poland; mzbawicka@iopan.pl (M.Z.); kuki@iopan.pl (P.K.); tkijewski@iopan.gda.pl (T.K.); 2 Arctic Research Centre, Department of Bioscience, Aarhus University, 4000 Roskilde, Denmark; lb@bios.au.dk; 3 Department of Ichthyology and Hydrobiology, St. Petersburg State University, 199034 St. Petersburg, Russia; p_strelkov@yahoo.com; 4 Department of Invertebrate Zoology, Faculty of Biology, Moscow MV Lomonosov State University, 119234 Moscow, Russia; mgantsevich@gmail.com; 5 Biology Department, Western Washington University, Bellingham, WA 98225, USA; mcdonald@udel.edu; 6 Centre for Integrative Genetics (CIGENE), Department of Animal and Aquacultural Sciences, Faculty of Biosciences, Norwegian University of Life Sciences, 1432 Ås, Norway; kristil.sundsaasen@nmbu.no (K.K.S.); mariann.arnyasi@nmbu.no (M.Á.); sigbjorn.lien@nmbu.no (S.L.); 7 Estonian Marine Institute, University of Tartu, 12619 Tallinn, Estonia; ants.kaasik@ut.ee (A.K.); kristjan.herkul@ut.ee (K.H.); jonne@sea.ee (J.K.)

**Keywords:** *Mytilus*, SNP, molecular population genetics, North Atlantic, environmental variables

## Abstract

Large-scale climate changes influence the geographic distribution of biodiversity. Many taxa have been reported to extend or reduce their geographic range, move poleward or displace other species. However, for closely related species that can hybridize in the natural environment, displacement is not the only effect of changes of environmental variables. Another option is subtler, hidden expansion, which can be found using genetic methods only. The marine blue mussels *Mytilus* are known to change their geographic distribution despite being sessile animals. In addition to natural dissemination at larval phase—enhanced by intentional or accidental introductions and rafting—they can spread through hybridization and introgression with local congeners, which can create mixed populations sustaining in environmental conditions that are marginal for pure taxa. The *Mytilus* species have a wide distribution in coastal regions of the Northern and Southern Hemisphere. In this study, we investigated the inter-regional genetic differentiation of the *Mytilus* species complex at 53 locations in the North Atlantic and adjacent Arctic waters and linked this genetic variability to key local environmental drivers. Of seventy-nine candidate single nucleotide polymorphisms (SNPs), all samples were successfully genotyped with a subset of 54 SNPs. There was a clear interregional separation of *Mytilus* species. However, all three *Mytilus* species hybridized in the contact area and created hybrid zones with mixed populations. Boosted regression trees (BRT) models showed that inter-regional variability was important in many allele models but did not prevail over variability in local environmental factors. Local environmental variables described over 40% of variability in about 30% of the allele frequencies of *Mytilus* spp. For the 30% of alleles, variability in their frequencies was only weakly coupled with local environmental conditions. For most studied alleles the linkages between environmental drivers and the genetic variability of *Mytilus* spp. were random in respect to “coding” and “non-coding” regions. An analysis of the subset of data involving functional genes only showed that two SNPs at Hsp70 and ATPase genes correlated with environmental variables. Total predictive ability of the highest performing models (*r*^2^ between 0.550 and 0.801) were for alleles that discriminated most effectively *M. trossulus* from *M. edulis* and *M. galloprovincialis,* whereas the best performing allele model (BM101A) did the best at discriminating *M. galloprovincialis* from *M. edulis* and *M. trossulus*. Among the local environmental variables, salinity, water temperature, ice cover and chlorophyll *a* concentration were by far the greatest predictors, but their predictive performance varied among different allele models. In most cases changes in the allele frequencies along these environmental gradients were abrupt and occurred at a very narrow range of environmental variables. In general, regions of change in allele frequencies for *M. trossulus* occurred at 8–11 psu, 0–10 °C, 60%–70% of ice cover and 0–2 mg m^−3^ of chlorophyll *a*, *M. edulis* at 8–11 and 30–35 psu, 10–14 °C and 60%–70% of ice cover and for *M. galloprovincialis* at 30–35 psu, 14–20 °C.

## 1. Introduction

Coastal ecosystems are highly complex and driven by multiple environmental factors. Local patterns represent the interplay of historical evolutionary processes (e.g., migration of species across regions, speciation or anagenesis) combined with separate and interactive effects of local environmental gradients on species (e.g., local adaptation as a result of ecologically based divergent selection between environments) [1,2]. These relationships are expected to be scale dependent, i.e., the regional genetic pool sets the range of variability in local populations and within these constraints, environmental stress plays a central role in shaping populations [3,4]. The formation and removal of barriers have occurred repeatedly over geologic time [5] and such broad scale processes modify inter-oceanic migration of shallow coastal marine faunas. Barrier formation fosters speciation as the original range of a species is divided up and divergent changes in the isolated populations are expected [6,7,8,9]. Genetic analyses shed light on the origin of populations and individuals and their range expansion through geological times. Differences in the local environment result in the presence of locally adapted populations [10] with varying potential to respond to differential environmental conditions [11]. Currently, it is still poorly known if and how variability in the local environment is reflected in the genetic structure of populations [12,13,14].

The blue mussel complex, *Mytilus* species, has a wide distribution in coastal regions of the Northern Hemisphere [15,16,17,18]. The species are an important ecosystem engineers in the intertidal and subtidal hard-bottom communities, where the adults are attached by byssus threads to rocks or stones. Such mussel beds are highly productive, and besides being the primary food source for birds, fish and invertebrates, they provide habitat and refuge for numerous organisms [19]. The *Mytilus* complex includes three incompletely isolated species of marine mussels, *Mytilus edulis* (Linne, 1758), *Mytilus trossulus* (Gould, 1850) and *Mytilus galloprovincialis* (Lamarck, 1819). Recent research on the distribution of the species complex evidently reflects a history of inter- and trans-oceanic dispersal, secondary contact and hybridization, besides the distribution effect brought about by human activities [18,20,21,22,23,24,25,26]. The complex originated from *M. trossulus* in the Pacific and invaded the Arctic Ocean from the Pacific Ocean around 3.5 million years ago (mya) through the Bering Strait [27,28,29]. During glacial periods, the Bering Strait closed, and allopatric speciation resulted in the evolution of *M. edulis* in the Atlantic. Since then *M. edulis* has spread to large parts of the Atlantic, and due to apparent low gene flow the populations of *M. edulis* on each side of the Atlantic are genetically distinct [30,31]. An allopatric isolation approximately 2.5 mya resulted in speciation between *M. edulis* and *M. galloprovincialis* [27,28,32,33] with secondary introgression and contact occurring around 0.7 mya [34]. Between interglacial periods 46,000 and 20,000 years ago, *M. trossulus* reinvaded the Arctic Ocean [22,35]. From there, it invaded both sides of the Atlantic resulting in the *M. trossulus*/*M. edulis* hybrid zones along North American and European coasts [20,21,36]. In general, *M. trossulus* is considered as the cold-water adapted species, *M. edulis* resides in temperate areas and *M. galloprovincialis* tolerates higher temperatures and has the most southerly native distribution of these three species in Europe in the Mediterranean and Black Sea. Moreover, *M. galloprovincialis* has successfully invaded temperate waters in other geographic areas such as South Africa, North and South America, Asia and Australia [37,38,39,40,41].

Today the species are in contact in several places in the North Atlantic with both co-occurrence and interbreeding [18,20,21,25,36,42,43,44]. The presence of *M. edulis* has been reported in the northern part of the Atlantic and European seas from the White and Barents Sea to the Atlantic coast of southern France and *M. galloprovincialis* is in the Mediterranean Sea, Black Sea and along the Atlantic coastline of Western Europe, including the British northern islands [21,24,30,36,45,46,47]. Until recently the documentation of *M. trossulus* was limited to the North Pacific, eastern Canada and the Baltic Sea [36], but more recent research has recognized its occurrence on the coasts of Scotland, Iceland, the Barents Sea, the White Sea, Norway and northwest Greenland [17,18,20,25,26,48,49].

As seen above *Mytilus* spp. have large inter-regional genetic differentiation, but it is poorly known if and how much this variation is correlated with local environmental conditions. In general, three important local environmental gradients have been recognized in intertidal hard bottom areas inhabited by *Mytilus* spp.: wetness, exposure to wave action and salinity [50]. The wetness gradient is primarily defined by the availability of solar irradiance. However, tides and waves significantly modulate the gradient, i.e., macrotidal shores (North Atlantic) have more pronounced wetness gradient compared with microtidal shores (Mediterranean and Baltic Seas). Gently sloping areas dissipate the wave energy arriving at the shore, while steeper sloping shores experience much greater physical impact. The salinity gradient occurs in estuaries and semi-enclosed seas. In order to cope with all these different environmental conditions local adaptation is expected to shape the genetic structure of local populations and thereby notable shifts in the frequencies of different alleles are expected along the most important environmental gradients [12,51]. This genetic differentiation can be expressed as differences in resistance to wave exposure [52], different responses to temperatures [53,54], heavy metals [55] and salinity [24,36]. Telesca et al. [56] recently studied links between environment and the shell shape of *Mytilus* spp. and reported salinity among the most important environmental drivers in the North Atlantic and Arctic regions. However, other authors indicate temperature and food concentration as prime environmental factors affecting distribution and geographic range of *Mytilus* populations [57,58,59].

The aims of this study are to provide a basic genetic knowledge on the current distribution ranges of the *Mytilus* species complex in the North Atlantic and Arctic at different geographical scales and to quantify if and how much environmental variables explain the genetic diversity of *Mytilus* spp. Our models included regions as well as key environmental variables known to drive the local variability of *Mytilus* spp. When the between-region variability is important in the models then this suggests that the genetic patterns of *Mytilus* spp. are highly due to the historical colonization of different regions [36]. On the other hand, when environmental variability strongly modulates the local genetic structure independent of regions, then local adaptation processes are expected to be very important. As local patterns represent the interplay of historical evolutionary processes and local adaptation as a result of ecologically based divergent selection between environments [1,2] we expect interregional differences to account for a large part in the genetic variability, but within region we assume that environmental variability strongly modulates the genetic differentiation, with larger differentiation expected in areas characterized by large ranges of environmental gradients. Ultimately, we expect that the linkage between environmental drivers and the genetic variability of *Mytilus* spp. is not random but is stronger for those alleles at genes opposing to alleles that are located in the non-coding regions.

## 2. Materials and Methods

### 2.1. Sample Collection and DNA Preparation

**Figure 1:**
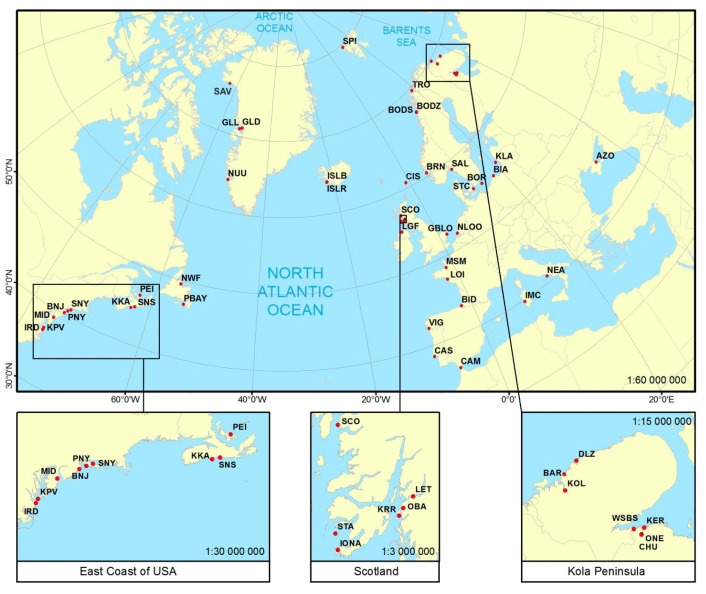
Geographic location of 53 *Mytilus* spp. sampling sites. See Table 1 for definition of site abbreviations.

*Mytilus* spp. samples, consisting of 1204 individuals of mixed ages and size 5–50 mm were collected from 43 localities in North America and Europe, including the arctic and subarctic regions. Eleven samples were obtained from North America (USA, Canada, Greenland); fourteen were obtained from arctic and subarctic regions of Europe (Iceland, Norway, Russia); seven from British Isles and Ireland; four from the Baltic Sea; four from the Atlantic coast of Europe; and three from the Mediterranean and Azov Seas. Forty-three samples consisted of 17 to 40 (mainly about 30) individuals, while two samples from Spitsbergen and Mont Saint-Michel consisted of 4 individuals (Table 1, Figure 1). DNA was isolated from the mantle tissue, stored in 96% ethanol or at −70 °C, using a modified CTAB method according to Hoarau et al. [60]. The individuals of *M. trossulus* from North-west Greenland, Savissivik (SAV), identified in Bach et al. [26] were used as reference samples of *M. trossulus*. Two populations, Indian River, Delaware (IRD) from the Atlantic coast of North America and Loire (LOI) from the Atlantic coast of North Europe, provided reference samples of *M. edulis* [25]. Populations from the Atlantic coast of South Europe at Bidasoa, Spain (BID) and from the Azov Sea (AZO) were reference samples of *M. galloprovincialis* [17,25]. A few samples used in our earlier works (ISLR in [17], ISLB and AZO in [61], BAR, ONE, LET and VIG in [21]) were genotyped with a set of single nucleotide polymorphisms (SNPs) for the purposes of this study. Except for these seven samples, results of genotyping with the same set of SNPs [25,26,37,38,62] were included in this study as shown in Table 1.

### 2.2. SNP Genotyping

SNPs identified in the present study and in earlier work [21,24,25] were used for genotyping of North America and Europe mussel samples. The possible effect of the SNPs, resulting in change or no change in amino acid sequence (non-synonymous or synonymous changes, respectively), was predicted on the basis of the open identified reading frames (Table S1). *Mytilus* individuals were genotyped using the Sequenom MassARRAY iPLEX genotyping platform [63]. Putative SNPs were designed based on DNA sequences obtained by us for 16 specimens and from GenBank DNA and RNA sequences of *M. trossulus*, *M. edulis* and *M. galloprovincialis* individuals from Europe and North America [21,24,25,62] (Table S1). Candidate SNPs were selected randomly and on the basis of genotyping quality (positive results for at least 90% of samples) from 385 putative SNPs. They were enriched with interspecific SNPs (*M. edulis*, *M. trossulus*, *M. galloprovincialis*) [21,24] and were tested on 300 specimens of *Mytilus* collected from geographic regions including Europe, North and South America and New Zealand. Assays were designed for 79 SNPs.

### 2.3. Data Analysis

#### 2.3.1. Genetic Diversity

Arlequin v.3.5.1.2 [64] was applied for population genetic analysis and to estimate genetic diversity, proportion of polymorphic SNP loci (*P_O_*) and minor allele frequency (MAF). Fifty-one samples consisting of at least 11 individuals were used. Expected (*H_E_*) and observed heterozygosity (*H_O_*) in each population was calculated using the whole SNP data set. The statistical significance of inbreeding coefficient *F_IS_* (>0) was tested by 10,000 permutations of alleles between individuals. Departures from Hardy–Weinberg equilibrium (HWE) were tested by exact test and significance was determined by Markov chain Monte Carlo simulations. Pairwise analysis was used to calculate mean pairwise *F_ST_* values defining population differentiation. *F_ST_* values at individual SNPs were calculated using the AMOVA function. Permutation testing with 1000 iterations was used to calculate *p*-values for mean *F_ST_* and locus-by-locus *F_ST_* values. Arlequin was also used to detect loci under selection. The neutral distribution of *F_ST_* was simulated with 30,000 iterations at a 95% confidence level.

The false discovery rate (FDR-BY) was applied to the significance threshold (*α* = 0.05) to control for multiple testing [65,66,67]. GENEPOP 4.1 [68] was used to test for linkage disequilibrium (LD) between all pairwise combinations of 66 polymorphic loci (Table S1) out of those assayed for the three groups of reference populations (*M. edulis*, *M. galloprovincialis* and *M. trossulus*). For a pair of diploid loci, no assumption was made about the gametic phase in double heterozygotes. *F_ST_* distance measures in the Newick format, obtained in POPTREEW [69], were used to construct a neighbor-joining (NJ) tree illustrating the genetic relationships among populations. 16 SNP markers (marked in Table S1 as HI) with alleles having high frequency in pure *M. trossulus* (about 90–100%, Table S2) were chosen to calculate the hybrid index score (HI) as described in Wenne et al [25]. These alleles distinguish *M. trossulus* from *M. edulis* and from *M. galloprovincialis*. A score of zero is a ‘pure’ *M. edulis*, whereas a score of one is a ‘pure’ *M. trossulus*. HI was calculated for individuals and averages for each population to assess the degree of introgression between *M. edulis* and *M. trossulus*. The *M. galloprovincialis* and *M. galloprovincialis*/*M. edulis* populations were excluded from above analysis. NewHybrids v1 [70] was used to estimate the posterior probability that individuals fall into each of the six genotypic categories (or classes corresponding to hybrid categories): native *M. trossulus*, *M. edulis*, F1 hybrids, F2 hybrids and 2 types of backcrosses. The same set of 16 SNP markers (marked in Table S1) with allele characteristic for pure *M. trossulus* (about 90–100%) used in HI analysis were chosen.

#### 2.3.2. Population Genetic Differentiation and Structure

Two methods were carried out to characterize population structure. In the first method clustering analysis was carried out with STRUCTURE v. 2.3.3 software [71,72]. STRUCTURE was used under the model assuming admixture, ignoring population affiliation and allowing for the correlation of allele frequencies between clusters. The admixture model used in this analysis allows individual structure with mixed ancestry, meaning that fractions of the genome could have come from different ancestors. Values of genetic clusters (*K*) were estimated by computing likelihood over 10 runs for values of *K* ranging from 1 to the study number of populations plus 3. At the plateau of the graph curve (plot of likelihood against K), the value of *K* captures the main structure of the populations. The best-fit number of genetic clusters was determined by calculating the logarithmic probability (LnP(K) and using the ΔK method [73]. Threshold q-values of 0.2 were used as a criterion to separate hybrids and pure mussels [74]. Individuals were considered residents if q > 0.8 for the area where they were sampled. Individuals with q-values from 0.2 to 0.8 were considered to be potentially admixed, as they could not be readily assigned as residents or migrants [75]. A Monte Carlo Markov Chain was run for 100000 iterations following a burn-in period of 50,000 iterations. In the second method, correspondence analysis (CA; [76]), implemented in GENETIX [77], was used for visualizing the genetic substructure at population and individual levels. They are presented as a scatter plot, with the axes representing the contribution of inertia of the data matrix in a way that can be considered analogous to the total variance in allelic frequency [76]. Each dot represents a population or an individual.

#### 2.3.3. Environmental Variables

Regional-scale proxies for weather and local environment were obtained from different online databases. A mean climatology for wind speed at 50 m above the surface of the earth was obtained from https://power.larc.nasa.gov/data-access-viewer/ and percent total cloud amount from http://daac.ornl.gov/ [78]. This climatology collection was compiled from existing data sources and algorithms and was designed to satisfy the needs of modelers and investigators of the global carbon, water and energy cycle. The site data were interpolated as a function of latitude, longitude and elevation using thin-plate splines [78]. Solar energy and precipitation were obtained from http://worldclim.org/version2 [79]. Sea water temperature and salinity were obtained from World Ocean Atlas 2018 at https://www.nodc.noaa.gov/cgi-bin/OC5/woa18/woa18.pl. Average swell height was obtained from the Global Atlas of Ocean Waves at http://www.sail.msk.ru/atlas/ and tide height from AVISO+ Satellite Altimetry Data at https://www.aviso.altimetry.fr/en/data/products/auxiliary-products/global-tide-fes/description-fes2014.html. The product Sea surface height amplitude due to non-equilibrium ocean tide at M2 frequency—FES2014b was used. Sea ice concentration (expressed as a mean percentage of ocean area covered by sea ice) was obtained from the Goddard Space Flight Center, Sea Ice Concentrations from Nimbus-7 Scanning Multichannel Microwave Radiometer and the Defense Meteorological Satellites Program Special Sensor Microwave/Imager passive microwave data (https://daac.ornl.gov/cgi-bin/dsviewer.pl?ds_id=981).

These original data were re-gridded by the National Snow and Ice Data Center from their original 25-km spatial resolution and EASE-Grid into equal angle Earth grids with quarter degree spatial resolutions in latitude/longitude [80]. Water chlorophyll *a* was obtained from the Ocean Color Climate Change Initiative dataset, Version 3.1, European Space Agency, available online at http://www.esa-oceancolour-cci.org [81]. Nutrient pollution was obtained from the World Ocean Atlas 2013 at https://www.nodc.noaa.gov/OC5/woa13/woa13data.html. The spatial and temporal resolution of datasets, data range and units are shown in Table 2. The environmental variables data for 53 *Mytilus* samples are shown in Table S3.

As data were originating from multiple sources, the environmental datasets have different temporal range. Here, these climatological time series are used to characterize typical environmental conditions (i.e., averages) of the regions of interest over a very long period of time. In the context of this study, some differences in timing among environmental datasets do not pose any significant problems as regional climatological averages are expected to change over the centuries and millennia rather than a few years. In addition, the environmental datasets also had different spatial resolution from 0.04 to 2°. However, in the current study we focused on large spatial patterns (interregional and regional) of *Mytilus* spp. that vary tens of degrees in the South-North and East-West direction. Thus, the grain of environmental variables is sufficiently detailed relative to the grain of study.

#### 2.3.4. Relationships between Environment and Allele Frequencies

Multicollinearity can be an issue with any spatial modeling algorithm when deciding which environmental variables are of ecological interest. Thus, prior to modeling, multicollinearity was checked with a Pearson correlation analysis in order to exclude highly correlated variables from the model. Most variables were only weakly intercorrelated at *r* < 0.3. Temperature was negatively correlated with ice cover (*r* = −0.87), but this value did not severely distort model estimations and subsequent prediction [82].

The relationships between different environmental variables and allele frequencies of *Mytilus* spp. were explored using the boosted regression trees technique (BRT). Besides environmental variables shown in Table 2, we added the variable region into our analysis (with the levels of the variable region defined as follows: North Atlantic Ocean, Baltic Sea, Barents Sea, Mediterranean and Black Seas). This enabled us to quantify the relative contribution of interregional variability vs the variability due to each environmental variable independent of region in the models of allele frequencies. When the between-region variability is important in the models then this suggests that the genetic patterns of *Mytilus* spp. are mostly due to the historical colonization of different regions by *Mytilus* spp. On the other hand, when environmental variability strongly modulates the local genetic structure independent of regional variability then local adaptation processes are expected to be very important.

The BRT is a technique that combines the strength of machine learning and statistical modeling; it avoids starting with a data model and rather uses an algorithm to learn the relationship between the response and its predictors [83]. The predictive performance of BRT models is superior to most traditional modeling methods (see, e.g., comparisons with GLM, GAM and multivariate adaptive regression splines, [84,85]). BRT performs relatively similar or sometimes even slightly better than other machine learning methods such as Random Forest (RF) [86]. We have recently used the latter technique to test if the genetic differentiation of populations of marine species may be related to any of the key environmental variables known to shape species distributions [12].

The BRT iteratively develops a large ensemble of small regression trees constructed from random subsets of the data. Each successive tree predicts the residuals from the previous tree to gradually boost the predictive performance of the overall model. The final BRT model is a linear combination of many trees (usually hundreds to thousands) that can be thought of as a regression model where each term is a tree. Although BRT models are complex, they can be summarized in ways that give powerful ecological insight [87,88]. R package gbm [89] was used for fitting the BRT models. When the locus had only two possible alleles a Bernoulli loss function was used for modeling the frequency while a multinomial loss function was used when three alleles where possible. Model predictions were determined by the maximal class probability (e.g., when, as predicted by the model, the probability of allele A was 0.3, the probability of allele C was 0.36 and the probability of allele T was 0.34, allele C was used as the model prediction).

Using BRT requires specification of the learning rate and number of fitted trees parameters. Decreasing (slowing) the learning rate increases the number of fitted trees required, and in general a smaller learning rate (and larger number of fitted trees) are preferable, conditional on the number of observations and time available for computation. The usual approach is to estimate the optimal number of trees with an independent test set or with cross validation (CV), using deviance reduction as the measure of success. We used 10-fold CV and learning rate 0.01 to estimate the optimal number of trees for each analysis with 20,000 trees as the limit. This strategy guaranteed that the rule-of-thumb of at least 1000 trees used was followed for each analysis. Typically, the number of trees used was larger than 5000. Relative importance of predictors should only be considered in the context of the model’s total predictive ability. To assess this, an adjusted count pseudo-R-squared was calculated for each model M as follows. First the prediction inaccuracy of model M was calculated as the frequency of incorrect predictions based on the CV predicted values of model M. Then the null model prediction inaccuracy was calculated as the frequency of the incorrect predictions of an intercept-only model. The ratio of the two inaccuracies can be interpreted as the relative prediction inaccuracy of model M and thus the pseudo-R-squared calculated as one minus the ratio is the relative prediction accuracy of model M.

In order to assess if linkages between environmental drivers and the genetic variability of *Mytilus* spp. are stronger when the respective alleles are located in the coding regions, we ran ANOSIM analysis to examine differences in the patterns of variation in genetic variability between the "coding" and "non-coding" regions. Prior to analysis, a Bray-Curtis similarity matrix was calculated using raw data (untransformed) of the BRT modeling results (i.e., variability explained by different environmental variables in each separate BRT models). Environmental variables responsible for the observed genetical differences were identified by the SIMPER analysis. The ANOSIM and SIMPER analyses were performed using the PRIMER 7 software [90].

## 3. Results

### 3.1. SNP Validation

Genotype results obtained with seventy-nine candidate SNPs were collated for 1469 mussels from 53 samples. For 11 SNPs, the assay did not provide an acceptable quality score, and two were monomorphic in all samples. Sixty-six were polymorphic. Of these 66 SNPs, 54 could be scored in all 53 samples and the remaining 12 could be scored in a subset of 16 samples. SNP annotation is presented in Table S1. Sixty of these SNPs have been described in the study of the European, Greenland and New Zealand populations of the *Mytilus* mussels [21,24,25,62]. The remaining 6 SNPs were newly characterized in the present study. Open reading frames (ORF) were identified in all, but eleven of the fragments. Of the 66 SNPs, 50 (90.9%) were located in coding regions, among which only 4 were nonsynonymous and 5 (9.1%) were located in non-coding regions. The population comparisons were performed mainly on the basis of 54 SNPs, but for comparison, some analyses were carried out using the set of 66 SNPs (16 populations).

Very little linkage disequilibrium between pairs of 66 markers was found in the three groups of *Mytilus* spp. populations. Only five pairs of loci out of a total of 2145 were in highly significant linkage disequilibrium (*p* < 0.001). Four were found between SNPs localized in the same fragment (BM57A vs. BM57D, BM5B vs. BM5D, BM9B vs. BM9C and BM21B vs. BM21C) and concerned *M. galloprovincialis* and *M. edulis* populations. The remaining pair consisted of different gene fragments (BM201C vs. BM203B) and concerned only *M. galloprovincialis* populations. *F_ST_* values at individual SNP markers ranged from 0.01 to 0.85. Deviation from expectation of neutrality based on a null distribution of *F*_ST_ values was identified in 41 SNPs which had *F_ST_* values significantly different from zero by outlier tests carried out for all studied populations (Table S1).

### 3.2. Genetic Diversity and Hardy–Weinberg Equilibrium

The level of polymorphic SNPs (*Po*) ranged from 37.04% to 88.9% among populations (Table 1). The lowest values were observed in American and European *M. edulis* populations, particularly in the population from Nuuk (Greenland NUU) and from the Atlantic coast of Europe (NLOO). The highest levels of polymorphism were found in Norway (Bergen BRN) and in the Baltic Sea (STC). There are also high levels of polymorphism in the Arctic and Subarctic populations (e.g., KOL, KER, GLD), where hybridization between *M. edulis* and *M. trossulus* also occurs, with 77–85% of the SNPs being polymorphic (Table 1). Among populations from the Hebrides Scotland, where there is an *M. galloprovincialis* introgression, about half of the SNPs were polymorphic (e.g., IONA, STA).

Most SNP loci were in Hardy–Weinberg equilibrium (HWE) in the vast majority of the study populations. The largest fraction of SNPs that were not in HWE (*P* < 0.05) after FDR-BY correction was observed in the population from Loch Etive, and in the Arctic and Subarctic populations from North Russia (BAR, CHU, KOL, KER), Newfoundland and northwest Greenland (Maarmorilik Fjord), reaching values from 40% to 58% of SNPs (Table 1). Observed heterozygosity (*H*_o_) for 54 loci among 50 populations was in general lower than expected (*H*_e_) in most of the populations. The greatest difference can be seen in the populations from North Russia (BAR, CHU, KOL, KER), Newfoundland (PBAY), Maarmorilik fjord (GLL) and in the Loch Etive (LET) population, where *H*_o_ is about half the value of *H*_e_. The highest values of *H*_o_ were observed in the populations from Atlantic coast of Spain, especially from the Camarinal (CAM), Scotland Hebrides (STA), Danish Straits (STC) and the lowest at populations from Russia White (KER) and Barents Sea (DLZ). The highest gene diversity and differences within population can be observed in populations from Bergen (BRG), Loch Etive (LET), Newfoundland (PBAY) and Danish Straits (STC), where many hybrids were also present (Table 1). The *F_IS_* measures (averaged over all polymorphic loci in each population) show a significant excess of homozygotes in 29 populations. Very large (>0.6) values of *F_IS_* for 30% to 50% of the loci were observed in populations from Russia (KOL, BAR, KER), Newfoundland (PBAY), Maarmorilik fiord (GLL) and in the Loch Etive (LET) population, where hybridization between *M. edulis* and *M. trossulus* occurs. In a few cases, *F_IS_* had values of 1, indicating the existence of two alternative homozygotes without any heterozygotes, mainly in populations from Russia in the White Sea and Barents Sea (e.g., BM202A, BM54A and BM92B in KER, KOL, BAR) (Table S4).

### 3.3. Genetic Variation and Differentiation among Populations

**Figure 2:**
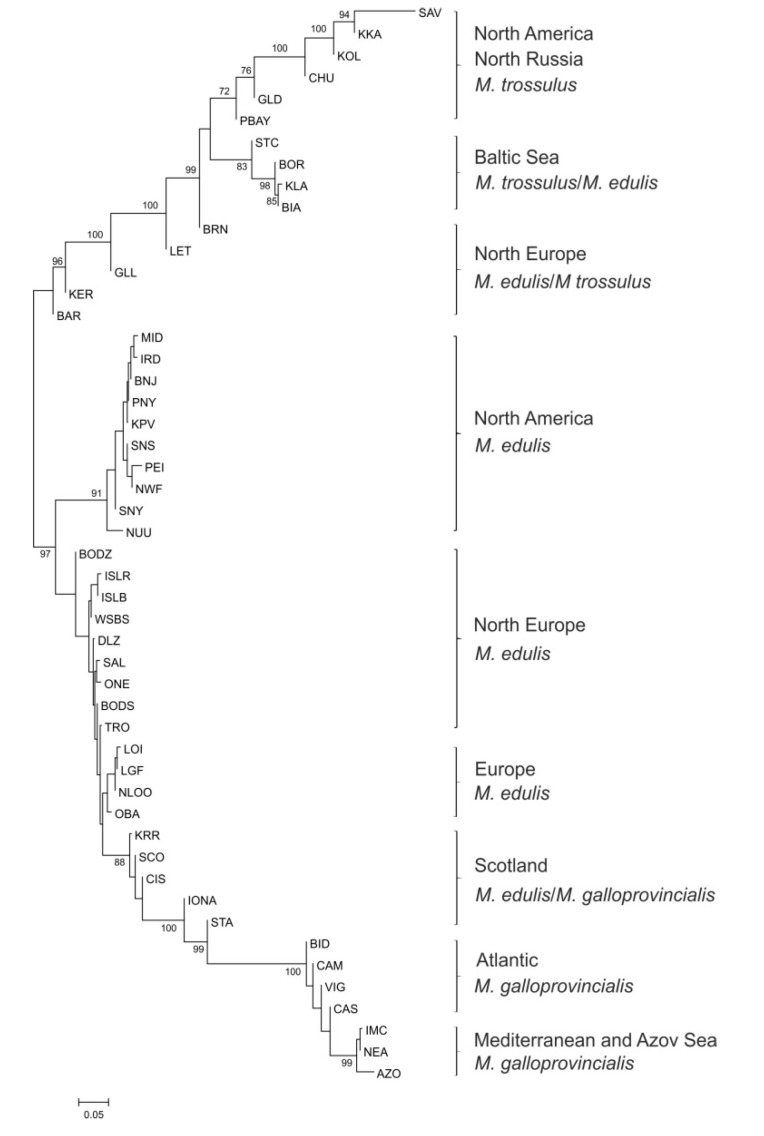
Neighbor-joining tree of 50 *Mytilus* populations based on the *F_ST_* distance matrix from allele frequencies of the nucleotide polymorphism (SNP) loci. Population codes as shown in Table 1.

The NJ tree showing the genetic relationships of 50 samples (those with more than 16 individuals) was constructed based on the *F_ST_* distance matrix (Figure 2). Besides the 3 main groups representing *M. edulis*, *M. trossulus* and *M. galloprovincialis*, the NJ tree revealed nine well-supported clades with strong evidence of geographic structure and different admixture of the main *Mytilus* taxa with marked differences between them. Populations of *M. trossulus* from North America and North Russia grouped separately from Baltic Sea *M. trossulus*/*M. edulis* and other mixed *M. edulis*/*M. trossulus* populations from Europe (Russia—KER, BAR, Norway—BRN, Scotland—LET and Greenland—GLL). The tree shows three separate groups which contain individuals of *M. edulis*: first, the populations from North America (USA, Canada, Greenland), second, North European populations (Arctic and Subarctic regions: Russia, Norway, Iceland), and third, populations originating from the Atlantic coast of Europe (France, Great Britain, Netherlands). In addition, one clade was composed of Scotland *M. edulis*/*M. galloprovincialis* populations. Populations of Atlantic and Mediterranean *M. galloprovincialis* formed two separate groups. Pairwise *F_ST_* values were significant after FDR-BY correction between most of the pairs of populations (Table S5). The greatest differences were observed between *M. trossulus* from North America (SAV) and *M. edulis* from Netherlands (NLOO) (reaching *F_ST_* values as high as 0.841) whereas three groups of populations, *M. edulis* from America, *M. edulis* from Atlantic coast of Europe and Atlantic *M. galloprovincialis*, were mostly homogeneous (*F_ST_* ranging from 0 to 0.006).

### 3.4. M. edulis Populations Structure

**Figure 3:**
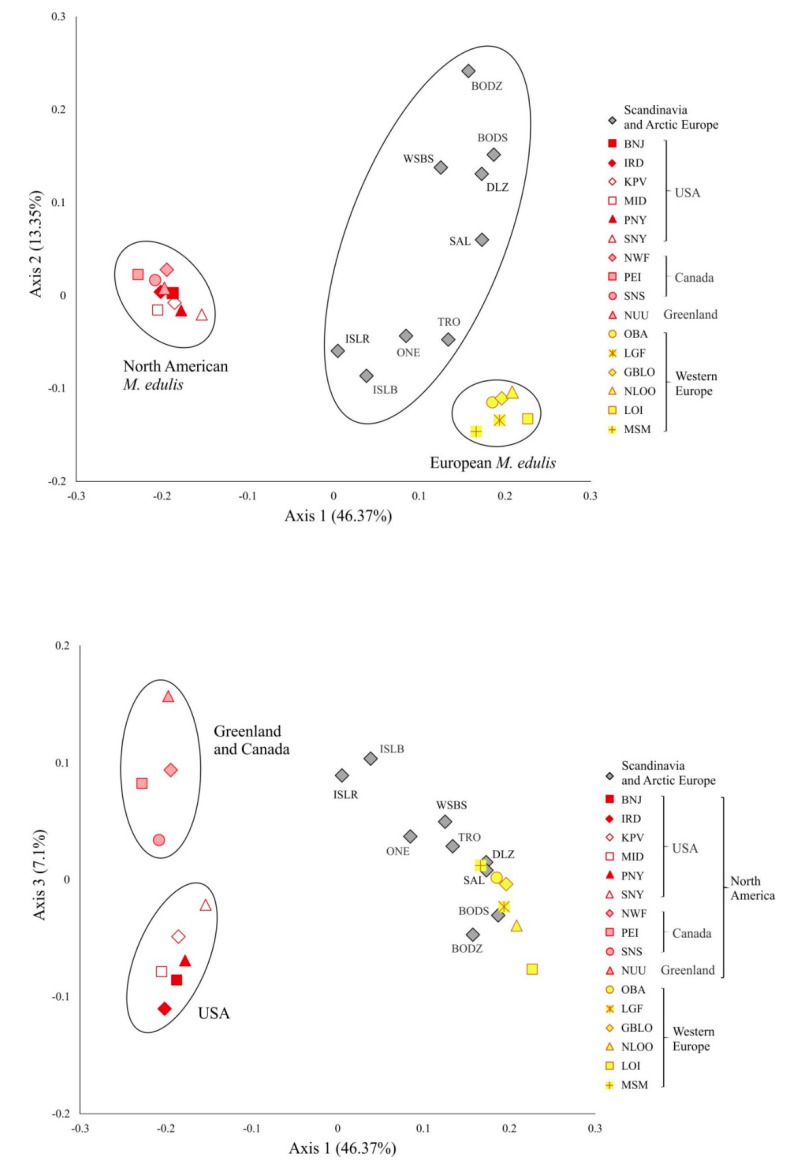
The first three axes of the correspondence analysis (CA) computed from the single nucleotide polymorphism (SNP) data on *M. edulis* from North Atlantic. Each point depicts a population.

**Figure 4:**
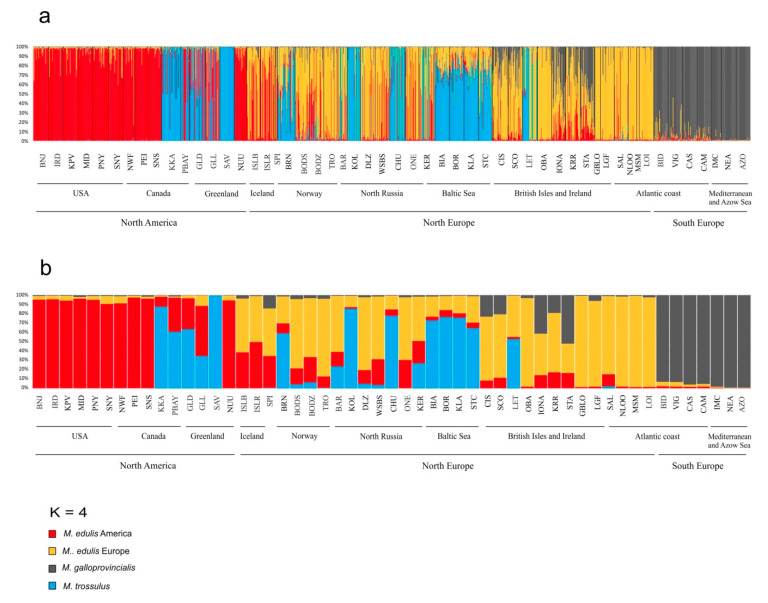
Plot from STRUCTURE analysis at *K* = 4 showing group affinities of 53 study samples. Each individual (**a**) or population (**b**) is represented by a single vertical line representing mean q value for individual and within the sample. Names of the sample sites are shown below bar plots, black vertical lines separate the sample sites.

Correspondence analysis was carried out to characterize *M. edulis* population structure. Analysis was performed for 54 SNP and 25 samples of *M. edulis* from North America, Scandinavia, Arctic and Western Europe (Figure 3). The first 2 axes accounted for 60% of the total variation, while axis 3 accounted for 7% of the variation. Axis 1 showed a clear separation between American and European populations of *M. edulis*. The American and Western Europe populations each form a very tightly clustered group while the Scandinavian and Arctic cluster, situated between them, had much greater dispersion. Along axis 3 there was a separation between populations from USA and those from Canada and Greenland. Structure analysis after applying the ΔK method [73] carried out for 54 SNPs and 53 samples, showed that the highest ΔK was found for *K* = 2, when differentiation was found between *M. trossulus* and other *Mytilus* taxa, then for *K* = 3, when *M. edulis* and *M. galloprovincialis* clustered separately. The LnP(K) value suggests further subdivision into four clusters with *M. edulis* splitting into two distinct groups: American and European populations (Figure 4a,b). This pattern of subdivision confirmed the results obtained using the NJ tree and the CA analysis. Scandinavian and Arctic populations with *M. edulis* genome and three individuals from Spitsbergen were potentially admixed of American and European *M. edulis*.

### 3.5. M. galloprovincialis Identity and Introgression

In STRUCTURE using *K* = 3, most of the individuals (97.38%) from seven populations of *M. galloprovincialis* (BID, VIG, CAS, CAM, IMC, NEA, AZO) were properly assigned to the original taxon. Only five individuals from the Atlantic coast (BID and VIG) were considered potentially admixed (*M. galloprovincialis* × *M. edulis*). Single individuals from British Isles populations (CIS, IONA, STA) were also assigned to the *M. galloprovincialis* cluster (*q* > 0.8), whereas from 30% to 90% of individuals from the British Isles, especially from Hebrides Scotland (IONA, STA) showed very high levels of admixture and were ambiguously assigned to *M. galloprovincialis* and *M. edulis*, with genome admixture values q from 0.2 to 0.8. Additionally, single individuals (total of 8) from Baltic Sea (BIA), North Europe (BODS, BODZ, TRO), Spitsbergen (SPI) and North America (SNS) were assigned to two clusters, mainly *M. edulis* and *M. galloprovincialis,* and rarely *M. trossulus* and *M. galloprovincialis* (BIA).

### 3.6. M. trossulus × M. edulis Hybrid Identification

Three different methods were used to identify hybrid individuals between *M. trossulus* and *M. edulis* taxa in 30 selected populations, comprising the 4 groups of populations: North America (13 populations), North Russia (7 populations), Scandinavia and Loch Etive (6 populations) and Baltic Sea (4 populations). Three populations from France (LOI), USA (IRD) and from Greenland (SAV) provided references of pure *M. edulis* and *M. trossulus* (Table 3). Structure analysis was used to estimate the admixture coefficient (*q*) for each individual by considering 54 allele frequencies, both species-specific and shared. The algorithm with the ΔK method [73] identified two clusters (*K* = 2) indicating *M. trossulus* and other *Mytilus* taxa (*M. edulis* and *M. galloprovincialis*). *M. galloprovincialis* was ignored in further analysis because only a few individuals from the analyzed populations were considered potentially admixed with *M. galloprovincialis* (0.73% of individuals, according to STRUCTURE analysis) (Table 3).

**Figure 5:**
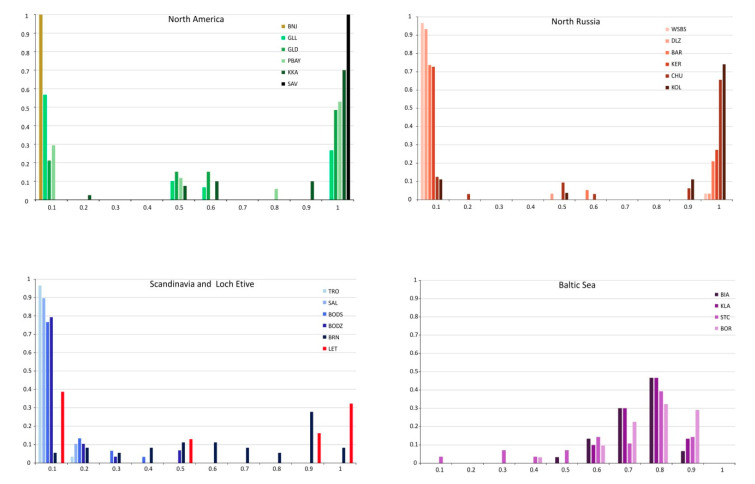
Frequency distribution of the score for a hybrid index giving the percentage of *M. trossulus* characteristic alleles. A score of zero is a pure *M. edulis*, whereas a score of one is a pure *M. trossulus*. Analysis was presented for four groups of populations: North America, North Russia, Scandinavia with Scotland and Baltic Sea. See Table 1 for definition and abbreviations.

A total of 16 SNP markers with alleles having high frequency in pure *M. trossulus* (species-specific) were chosen to detect hybrid individuals by calculating the *HI* score (Figure 5) and using the program NewHybrids. Ten populations from North America were identified as pure *M. edulis* by both Structure and NewHybrids, with an *HI* below 0.05. The remaining 4 populations from Canada (PBAY, KKA) and Greenland (GLD, GLL) showed a high diversity. *HI* varied from 0.37 in Greenland populations to 0.85 in a population from Nova Scotia (KKA), and *V_HI_* (variance of *HI*) reaches 0.43 in the PBAY population. Most individuals were identified as pure *M. trossulus* or pure *M. edulis* using both Structure and NewHybrids. The others were identified as hybrids with *q*- value about 0.5, however F1 and F2 hybrids could not be distinguished using the Structure analysis method. By contrast NewHybrids revealed F1 hybrids in these 4 populations with a probability mostly over 0.9. The percentage occurrence of F1 hybrids varied from 12% (PBAY) to 24% (GLD) of individuals. F2 hybrids were not identified, while only a single backcross to *M. trossulus* was found.

Among seven populations from North Russia only one from White Sea (ONE) was identified as pure *M. edulis*. In two others (KER, WSBS) all individuals were identified as pure *M. trossulus* or *M. edulis*. In the remaining 4 populations, Structure revealed a small number of hybrids in proportion ranging from 0.03 to 0.12. NewHybrids revealed from 0.03 to 0.09 F1 hybrids with a probability over 0.99 and a single *M. trossulus* backcross. The values of *HI* varied from 0.02 (ONE, WSBS, DLZ—the lowest proportion of *M. trossulus* characteristic alleles), through 0.28 (BAR, KER) to 0.82 (CHU, KOL with the highest proportion of *M. trossulus* characteristic alleles) (Table 3). Except for Bergen (BRG) all Scandinavian populations were characterized by very low *HI* values (0.03–0.07). Structure analysis showed pure *M. edulis* ranging from 0.86 to 1.0 in frequency with a few hybrids identified in populations from Bodø (BODS–0.03, BODZ–0.14). NewHybrids with a probability mostly over 0.8 revealed single F1, F2 hybrid (0.03) and *M. edulis* backcrosses (0.03–0.17) as shown in Table 3. Conversely, populations from Bergen and Loch Etive had pure *M. trossulus* and *M. edulis* taxa with *HI* reaching above 0.5. In Bergen, the percentage of hybrids reached 0.5. However, NewHybrids analysis with high probability revealed all hybrid categories in the population from Bergen and F1 hybrid and *M. trossulus* backcrosses in populations from Loch Etive. Populations from the Baltic Sea had low diversity with *HI* ranging from 0.63 to 0.72. Most of the populations showed a unimodal hybrid swarm with varying amounts of *M. trossulus* characteristic alleles (usually classified as *M. trossulus* backcrosses, *M. trossulus* or F2 hybrid). Only the Danish Straits population (STC) represented an admixture of the two taxa with only a single *M. edulis* individual and no F1 hybrids (Table 3).

### 3.7. Relationships between Environment and Allele Frequencies

Our BRT models showed that the used environmental variables described over 40% of variability in about 30% of the allele frequencies of *Mytilus* spp. (Table S6). For 30% of alleles, variability in their frequencies was only weakly coupled with environmental conditions, i.e., in these models the studied environmental variables described less than 10% in the variability of allele frequencies. Total predictive ability of the highest performing models (r^2^ between 0.41 and 0.79) were for BM101A, BM113A, BM11A, BM12A, BM17B, BM202A, BM202B, BM203D, BM21B, BM21C, BM26B, BM54A, BM62A, BM67C, BM92B. Interestingly, these alleles did the best at discriminating *M. trossulus* from *M. edulis* and *M. galloprovincialis* or *M. edulis* from *M. trossulus* and *M. galloprovincialis* (BM17B, BM21C and BM67C), whereas the best performing allele model (BM101A) did the best at discriminating *M. galloprovincialis* from *M. edulis* and *M. trossulus* (see result section above) (Table 1, Figures S1 and S2). In additional analysis involving 4 SNPs in known functional genes for 16 populations, BM116A and BM206A located in Hsp70 and ATPase genes were highly correlated with the following environmental variables: salinity, ice-cover, wave height and temperature.

As expected, inter-regional variability was important in many allele models, especially those explaining over 40% of variability in allele frequencies (Table S6). However, the inter-regional variability never explained more than 16% of variability in any of the studied allele frequencies. Similar to regional variability, salinity, temperature, chlorophyll *a* and ice cover contributed over 10% (sometimes nearly 40%) of the variability of allele frequencies. Overall, the predictive power of models only weakly reflected whether the variable region was included into the model or not (i.e., the average predictive power of the studied allele models with and without the variable region was 25.9% and 22.2%). Thus, judging from the correlative evidence only, historical evolutionary processes do not dominate over selection between environments.

In most cases changes in the allele frequencies along the studied environmental gradients were relatively abrupt and occurred within a narrow range of environmental variables. In general, regions of change in allele frequencies for *M. trossulus* occurred at 8–11 psu, 0–10 °C, 60–70% of ice cover and 0–2 mg m^−3^ of chlorophyll *a*, for *M. edulis* at 8–11 and 30–35 psu, 10–14 °C and 60–70% of ice cover, and for *M. galloprovincialis* at 30–35 psu, 14–20 °C (Figure S1).

When quantifying the strength of associations between allele frequencies and the environment in alleles located in “coding” or “non-coding” regions (ANOSIM and SIMPER analyses) the allele models of “non-coding” regions had higher component of inter-regional variability (7.8%) compared to those of “coding” regions (3.2%) whereas the effect of salinity was stronger for coding alleles (4.2%) than for non-coding alleles (3.4%). However, the observed differences were not statistically significant (*p* = 0.582). Thus, for the studied alleles the linkages between environmental drivers and the genetic variability of *Mytilus* spp. was random in respect to “coding” and “non-coding” regions.

## 4. Discussion

### 4.1. Distribution of Mytilus Taxa on the Coasts of North Atlantic

**Figure 6:**
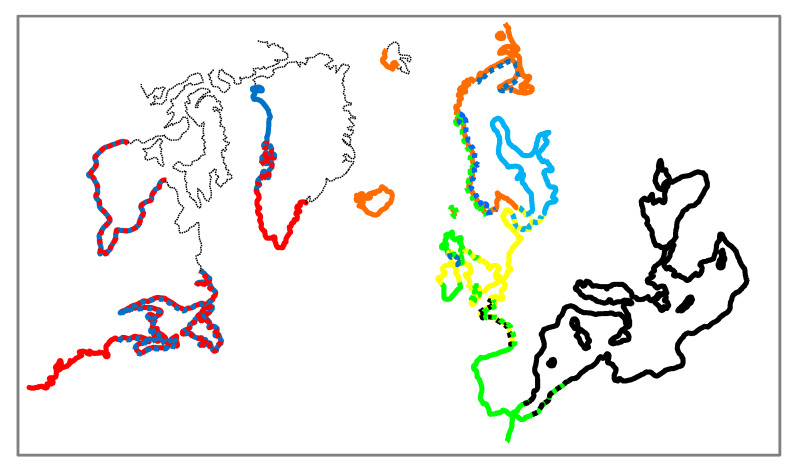
Map of *Mytilus* lineages distribution in North Atlantics according to original and literature data (see the text for references). *M. edulis* is depicted by red (American lineage), yellow (West European lineage) and orange (North European *M. edulis*, the product of intermingling between American and West European lineages), *M. trossulus* by blue (the Baltic lineage by light blue), *M. galloprovincialis* by black (Mediterranean lineage) and green (Atlantic lineage).

In this study we investigated spatial genetic variability in populations of the *Mytilus* species complex in the North Atlantic and adjacent Arctic waters at different geographical scales. Most of our results on taxonomic affinities of geographical populations are consistent with the earlier findings and complement the previous studies well. This allows us to provide a general picture on spatial distribution of mussel species (Figure 6) and intraspecific lineages and to discuss their evolutionary history. No such attempts have been undertaken since the introduction of SNP-based methods in blue mussel genetics in the early 2010 s (e.g., [21,44]).

According to our data the east coast of the USA is dominantly inhabited by *M. edulis,* while settlements of *M. trossulus* (as KKA and PBAY) are in Canada. In the literature, *M. edulis* populations are described to be distributed along the American continental coast from Delaware Bay (38.5° N) and north [57], while *M. trossulus* are found from the Gulf of Maine (44° N) and north [54]. Along the coasts of New Brunswick, Nova Scotia and Newfoundland the two species co-occur, hybridize and are generally distributed as partly overlapping mosaics [54,91,92]. It is not known how far north the species spans along the coasts of Canada. Intriguingly, the most northern genetically studied population, from Hudson Bay (58.5′ N) appeared to be predominantly *M. edulis* (the NCBI collection of the DNA barcode study of Canadian invertebrates [93]). Along the western coast of Greenland, *M. edulis* populations appear to be taken over by *M. trossulus* at 70.5° N (GLL, GLD) where these two species form a hybrid zone [25]. The northernmost site in Greenland (SAV, 76° N) is populated by pure *M. trossulus,* which was found to be more related to the North Pacific *M. trossulus* populations than the North Atlantic as for GLL and GLD [26]. At least two independent, heterochronous invasions from the Pacific were behind the diversity of *M. trossulus* in the Atlantic [22,26,35]. On the European coast of the Atlantic, the southern border of *M. edulis* is again located father south (LOI, 47° N) from that of *M. trossulus* (LET, 56° N). From our data, as well as from the literature [17,20,48], it is shown that while *M. edulis* is distributed mostly discontinuously along the coasts of Europe up to the very distribution border in the Pechora Sea (69.5° N, [18,20]), the distribution of *M. trossulus* is sparse with one large population in the inner Baltic Sea and small outposts in Loch Etive in Northern Scotland (LET), the Bergen area in Western Norway (BER), and harbor areas in the White (CHU) and Barents (KOL) Seas in Russia. Several more *M. trossulus* outposts in the White and Barents Seas, all from harbor areas, were also reported [20,94]. Single locus-based data indicated that *M. trossulus* could be more widespread in Western Norway [95] and in North-western Scotland [96]. In each of the outposts studied here, *M. trossulus* was found together with *M. edulis*. The northernmost population of *M. trossulus* in Europe – BAR is at 69° N. Only *M. edulis* populations were found in Iceland (ISLB, ISLR) and on Spitsbergen (SPI, 79° N, i.e., further north than northernmost *M. edulis* and *M. trossulus* in Greenland). Thus, taking into account only geographic distribution, it could be difficult to postulate that *M. trossulus* is a more cold-water adapted species compared to *M. edulis*. Therefore, analyses directly based on correlation between species distributions and temperature and other environmental proxies rather than simply latitude was performed in this study to understand what contemporary factors actually control biogeographical distribution of *Mytilus* species.

European *M. trossulus* are not genetically homogenous, but are subdivided into two clusters, Baltic populations and all other settlements. Distinctness of the Baltic mussel is primarily explained by its mixed nature due to the strong introgression of *M. edulis* genes (see [23,45] for more discussion). Other European *M. trossulus* populations are indistinguishable from the Canadian ones. The structure of mitochondrial DNA variation in these natural populations (e.g., LET), indicates either relatively long (thousands rather than hundreds of years) vicariance [22] or a long history of introgressive hybridization with *M. edulis* (Baltic mussel [12,24,97]). Singular or multiple natural invasions from continental populations of West Atlantic, most probably Holocene, better explain existence of *M. trossulus* populations in Scotland, Western Norway and the Baltic Sea.

*M. edulis* populations are subdivided into three clusters, the American and West European are most distinct, but internally quite homogenous, while North European (admixed) is heterogeneous and intermediate between the other two. The Danish Straits have been proposed as a border between the West- and the North European clusters [98]. The macrogeographic population structuring of *M. edulis* in Europe was revealed only in recent SNP-based research ([98], this study), but unrecognized in earlier mitochondrial (e.g., [23]) and allozyme (e.g., [99]) studies. Multilocus differences between populations from the two coasts of the Atlantic Ocean, most pronounced for the West European and the West Atlantic (American) clusters, were expected. But how do we explain the origin of the North European cluster that seems to be a product of intermingling between the West European and the West Atlantic ones? One hypothesis could be that in the course of colonization of Northern Europe by *M. edulis* (either post-glacial or after a hypothetical episode of climate-driven extirpation of mussels from the northern part of the area), the region was colonized both from Europe and from America. Subsequent gene flow homogenized mitochondrial diversity, but not the nuclear diversity through Europe. Indeed, mussels are prone to differential mitochondrial DNA introgression. For example, the Baltic populations, although being dominated by *M. trossulus* nuclear genes, are virtually fixed for mitochondrial haplotypes of *M. edulis* [32,100]. Atlantic *M. galloprovincialis* populations have been reported partly introgressed with *M. edulis* mitochondrial haplotypes [101,102].

*M. galloprovincialis* was underrepresented in our samples from outside its ancestral range in the Mediterranean (the Mediterranean lineage) and along the Atlantic coast of Iberian Peninsula (the Atlantic lineage). The complex contact zones with *M. edulis* in southern France [47] and on British Isles [103] were not sampled except for northwestern Scotland. Very few individuals of *M. galloprovincialis* from Scotland could be defined as ‘pure’ (i.e., with characteristic alleles > 80%), while the presence of *M. galloprovincialis* alleles was considerable in mixed populations with *M. edulis* from Scotland (average contribution of *M. galloprovincialis* alleles 20–50% in KRR, SCO, IONA, STA and CIS), but negligible (<5%) in other samples. The results of our study correspond to the results of the recent study by Simon and co-authors [98] on the distribution of *M. galloprovincialis* in Atlantic Europe and its hybridization with *M. edulis*. They discovered invasive populations of the Mediterranean lineage in multiple French harbors up to Le Havre north (49.5° N) and confirmed earlier records [18,95] of the Atlantic lineage of *M. galloprovincialis*) in Norway in the Ålesund area and Lofoten Iles (68° N). Each time *M. galloprovincialis* were found to hybridize with native mussels (Atlantic lineage of *M. galloprovincialis* or Western European lineage of *M. edulis* in France and North European – admixed lineage of *M. edulis* in Norway) and were being introgressed with their genes. In its turn, the reference natural Scottish *M. galloprovincialis* population was different because it represented the Atlantic lineage introgressed with the North European *M. edulis* genes. Further Simon et al. [98] re-analyzed reference samples from the SNP-based study of Mathiesen et al. [18] to show that *M. galloprovincialis* presence in the Arctic was overestimated considerably at the individual and population levels. The overestimation has been generated by using Atlantic *M. galloprovincialis* introgressed with *M. edulis* alleles as a reference sample, which reduced the actual diagnostic power of SNPs used by Mathiesen et al [18]. The only minor contribution of *M. galloprovincialis* genes to the genetic composition of Spitsbergen *M. edulis* populations was confirmed, which is in line with our observations. Occurrence of the Mediterranean mussel in Northern Europe well illustrates its long-known ‘superficial’ abilities as an invasive species, but also questions the prognostic power of species-distributions models (cf. [104]) predicting environmental requirements of a species biasing on conditions in its ancestral range.

### 4.2. Hybridization and Population Structure

Pure *M. trossulus* or *M. edulis* taxa and a low frequency of hybrid mussels, mainly F1 and single *M. trossulus* backcrosses, were found in the arctic and subarctic region in Russia and America (Figure 4 and Figure 5, Table 3). Large *F_IS_* values, deficiency of heterozygotes and high variance of *HI* (reaching 0.43 in PBAY) indicated high admixture of two taxa and a bimodal hybrid zone. This type of hybrid zone largely consists of genotypes resembling the parental forms, with only few intermediates [105]. This situation was previously presented in detail in relation to the Greenland populations [25]. A low frequency of hybrids in America was observed earlier by Riginos and Cunningham [36]. However, European populations where *M. trossulus* and *M. edulis* mussels coexist and hybridize present a completely different population structure. Pure taxa plus all hybrid categories were found in Norway and a large number of hybrids in other locations like Scotland and Baltic Sea [21,24,49]. However, in the Baltic Sea, in contrast to the Scotland and Norway hybrid populations, variance of *HI* was very low (reaching 0.07 in inner Baltic), pointing to a unimodal hybrid swarm. In Scotland and Norway, we observed intermediate genotypic distributions that consist of a slightly more even mixture of parental and hybrid genotypes. A characteristic feature of the Scandinavian and Arctic mussels with *M. edulis* genomes is admixture of American and European clusters (Figure 4a,b).

According to our data pure *M. galloprovincialis* individuals were not observed in the Arctic and subarctic region. Only single *M. edulis*/*M. galloprovincialis* hybrids were detected using SNP. From our data and the literature pure *M. galloprovincialis* populations have been found in the Mediterranean and Azov Sea [12,21], whereas low numbers of pure *M. galloprovincialis* individuals were identified on the coasts of France, Spain and the British Isles. The British Isles, especially Inner Hebrides Iona and Staffa Islands were characterized by very high percentage of *M. galloprovincialis* characteristic alleles and a very high level of admixture with *M. edulis* that reached 90% of the individuals. The other areas of the British Isles (like nearby Oban) that are not directly exposed to the warm Gulf Stream of the Atlantic Ocean were not characterized by such large *M. galloprovincialis* introgression. Dias et al. [96], using only one molecular marker (Me 15/16), showed potential admixture of *M. galloprovincialis* alleles throughout the northwest and northeast of mainland Scotland and the Shetland Islands.

### 4.3. Relationships between Mytilus Genotypes and Environmental Factors

In order to evaluate the role of environmental conditions in shaping local genetic structure we quantified correlative links between key environmental drivers and the genetic variability of the three species *M. edulis*, *M. trossulus* and *M. galloprovincialis* and their hybrids. We expected that interregional differences would account for a large part of the genetic variability of *Mytilus* spp., but within region environmental variability would strongly modulate the genetic differentiation [12,51]. Our analyses only partly confirmed this with inter-regional variability being important in many allele models (as evidenced by distinct lineages in different regions), but this variability never exceeded variability that can be attributed to selection between environments. On the other hand, for 30% of alleles environmental variability only poorly explained the allele frequencies. Nevertheless, there are also other factors and processes not involved in our analyses (e.g., prehistoric relicts, ocean currents and ship trafficking) that may contribute to the variability of the genetics of *Mytilus* spp. [48,94,98,106,107,108]. In the current study, we found species-specific alleles, which correlated with environmental variables and discriminated best *M. trossulus* from *M. edulis* and *M. galloprovincialis*. These alleles did not involve the functionally important genes, which products can affect resistance to stress and tolerance to changes in environmental variables [109].

Earlier literature suggests that salinity and temperature drive the patterns of hard bottom intertidal species including *Mytilus* spp. [50]. Although the *Mytilus* species-complex possesses a high degree of phenotypic plasticity, different species show differences in physiological responses to environmental conditions. In general, *M. trossulus* is the northernmost species, tolerant to cold waters, but also brackish waters, and is often found in areas which have been ice-covered in previous ice ages. *M. edulis* inhabits the cold, but temperate waters, but can also occur in brackish waters. The European southern range of *M. edulis* on the Atlantic coast overlaps with *M. galloprovincialis*, which is a temperate marine warm water species inhabiting more exposed locations in the south European/Mediterranean Seas, e.g., [47,98].

This study identified salinity as the primary driver for at least the distribution of *M. trossulus* and *M. edulis*. The strong effect of salinity was also expected as salinity varied over a large range in the study area. Several studies have documented that *M. trossulus* tolerates lower salinity better than *M. edulis* both as adult [20,24,45,49,110] and larvae [111,112]. An example of the salinity driven distribution is the occurrence of the species in the Baltic Sea. The Baltic Sea is a large epicontinental brackish waterbody with a permanent strong salinity gradient driven by the inflow of saline waters from the North Sea coupled with high riverine runoff. The salinity varies from freshwater in its most marginal parts to almost oceanic salinity (20 psu) in its entrance area. The low saline areas of the Baltic Sea are inhabited by populations with a predominance of *M. trossulus* genes, which are more tolerant to low salinities [12,24,113,114]. As shown in this and other studies [12] there is little introgression of *M. trossulus* in the entrance area of the Baltic Sea, but a strong introgression of *M. edulis* in the Baltic Sea proper such that no pure *M. trossulus* genotypes were identified. The segregation of *M. trossulus* in the brackish Baltic Sea and in lowered saline central Greenland fjords impacted by a freshwater input from snow melt and glacial input (as, e.g., GLD) is in contrast to the western Atlantic clade (e.g., KKA, PBAY) and the North Greenland (SAV) where *M. trossulus* is found at more open wave-exposed coasts. Similarly, Riginos and Cunningham [36] have concluded that *M. edulis* dominates in sheltered areas of low salinity and *M. trossulus* on wave-exposed open coasts in the Western Atlantic.

In the present study, temperature was also found as an important driver of the spatial patterns of genetics of *Mytilus* spp., but to lesser extent than salinity. Relationship between temperature and intertidal animals are complex. During submergence intertidal organisms mostly experience a stable water temperature whereas during emersion when exposed to air they often experience much warmer or colder climate. The wetness gradient (i.e., a combined product of solar irradiance, coastal morphology, tidal and wave regime) defines the realized effect of temperature on intertidal organisms. In our modeling, however, solar irradiance, tidal and wave regime had only marginal effects on the variability in the studied allele frequencies.

Adult *M. edulis* for example require summer temperatures of a minimum of 4 °C [115]. On the other hand, at 10 °C or above the larvae of *M. edulis* are favored over *M. trossulus* [54]. Similarly, ice cover favors the cold-water *M. trossulus* that often occurs in ice covered areas. While *M. edulis* has been shown to start their spawning in late spring, *M. trossulus* generally starts later in the summer [116,117], paving a possibility for *M. trossulus* to dominate areas with longer-lasting sea ice. Popovic and Riginos [118] summarized data on distribution and temperature of four *Mytilus* taxa in Northern Hemisphere and demonstrated that *M. galloprovincialis* inhabits waters characterized by much higher mean surface temperature in comparison with *M. edulis* and *M. trossulus*. *M. galloprovincialis* has physiological and metabolic adaptations to higher sea water temperature in comparison with *M. edulis* and *M. trossulus* [119,120,121,122]. As salinity, water temperature, sea ice and tidal range have been reported to be the most important factors driving the fitness differences in *Mytilus* hybrid zones, we expected that the taxa specific alleles would be correlated to some extent with the functional genes related hereto (e.g., adhesive foot protein gene; ubiquitin conjugates, hsp genes). *Mytilus* spp. attach to their substrate by byssus threads and studies have shown differences in preferences to wave action between *Mytilus* species [36,52]. In addition, a study on proteomic responses to acute heat stress found for example that *M. trossulus* tended to induce changes in Hsp levels at lower temperatures than the warmer-adapted *M. galloprovincialis* [122]. In this study, in a more targeted analysis based on functional genes, 2 SNPs, namely BM116A and BM206A located on Hsp70 and ATPase genes were correlated with environmental variables: salinity, ice cover, wave height and temperature. These genes can be involved in stress response in *Mytilus*.

Finding differences at the level of genotype/allele dependent gene expression in response to environmental variables requires a candidate gene approach and transcriptome analysis. Mantle transcriptome comparison among *Mytilus* taxa has been performed by Malachowicz and Wenne [123]. While Fraïsse et al. [44] demonstrated some SNP loci under selection (outliers), Popovic and Riginos [118] used transcriptome analysis for temperature selection studies in *Mytilus* specimens from natural environments. Their study performed on 24 individuals of *Mytilus* congeners collected from natural environments differing in temperature suggested a positive temperature-dependent selection as a factor influencing molecular divergence between warm and cold tolerant *Mytilus* taxa. Among thermotolerant candidate genes exhibiting differentiated expression were mentioned genes of oxidative stress response and cytoskeletal stabilization, including Hsp. The authors concluded that expression and sequence divergence can be correlated at the multigene level. Changes in transcriptome expression have been observed after exposure of *M. edulis* from the Gulf of Maine to increased temperature, CO_2_ induced acidification, and lowered food concentrations under laboratory experimental conditions [124]. Those changes in environmental factors are expected to occur under the predicted climate change scenario for the Gulf of Maine. Differentially expressed genes related to stress response, including upregulation of chaperones, aerobic metabolism, cellular stress and calcification were observed. Other reactions at the transcriptome level were observed in *M. edulis* and *M. galloprovincialis* larvae in response to elevated temperature [125]. Differences in thermotolerance have also been reported between western and eastern Atlantic populations of *M. edulis* [59]. A contraction of the western Atlantic *M. edulis* population northward by 350 km of Cape Hatteras has been a sign of serious displacements of populations, which should be expected as a consequence of global climate changes [57]. A northward shift of *M. edulis* and *M. galloprovincialis* populations in Europe in response to climate changes in the North Atlantic area has been also predicted by an individual-based model [58].

While climate reconstructions have demonstrated that the Earth has experienced oscillations in both warming and cooling periods over the past several millennia [126], the current rate of observed warming is unparalleled [57]. Ongoing global climate changes have increased the focus on the introduction and disappearance of species and especially on marine species with special emphasis on changes in temperature and ocean currents [62,127,128]. In the times of global climate changes, it is important to establish baseline knowledge of species distribution and abundance, but also ecosystem structure and function as well as level of genetic native biodiversity [62]; it is assumed that the climate changes will provide room for introduction or changes in the species distribution also with respect to blue mussels. Thus, the climate changes may result in introductions of an invader to existing mussel populations resulting in change of abundance of the native mussel species, but also hybridization and introgression between the species, e.g., [44]. Although *Mytilus* spp. display high plasticity to environmental changes, studies on ecological effects of introgression and hybridization points to significant differences in fitness, including less resilience in hybrids and backcrosses compared to parental species [42,62,129]. Prediction of how climate change may influence the redistribution of species is a key task in ecology conservation.

## 5. Conclusions

We studied the genetics of Amphi-Atlantic and Arctic populations of *Mytilus* mussels from over 50 locations using single nucleotide polymorphisms. We found three main groups of populations representing *M. edulis*, *M. trossulus* and *M. galloprovincialis* as well as nine clades with a strong evidence of geographic structure and different admixture level. Populations of *M. trossulus* from North America and North Russia grouped separately from introgressed *M. trossulus*/*M. edulis* Baltic Sea and other populations from Europe (Russia, Norway, Scotland) and Greenland. Three separate groups of *M. edulis* populations were found: pure American, American and European admixed in Scandinavia and Arctic and pure European. In addition, a separate group was composed of admixed Scotland *M. edulis*/*M. galloprovincialis* populations. As expected, populations of Atlantic and Mediterranean *M. galloprovincialis* formed two separate groups.

All three *Mytilus* taxa hybridize in the contact area and create hybrid zones. Hybridization and introgression with local congeners result in mixed populations. The mixed populations are mainly bimodal in the Arctic and Subarctic region in Russia and America for *M. trossulus* and *M. edulis* taxa. However, in other European populations intermediate genotypic distributions between *M. trossulus* and *M. edulis* (Scotland and Norway) or *M. edulis* and *M. galloprovincialis* (France, Spain and the British Isles) were observed. Finally, the hybridization zone can take the unimodal form as in the inner Baltic, creating a hybrid swarm.

Correlative species distribution models are frequently applied as a sophisticated tool to improve our understanding of the relationship between environment and biota. Here, we applied boosted regression trees modeling technique to evaluate the role of environmental conditions in shaping local genetic structure of *Mytilus* spp. Importantly, the linkage between environmental variables and the frequency of most alleles was not affected whether alleles were located in the coding or non-coding regions.

However, in a more targeted analysis based on functional genes, two SNPs at Hsp70 and ATPase genes showed linkage with environmental variables. In addition, inter-regional variability was important in many allele models, but this source of variability did not prevail over variability in local environmental variables.

Among the studied environmental variables salinity, water temperature, ice cover and chlorophyll *a* concentration were the greatest predictors among different allele models in the studied populations of *Mytilus*. In most cases changes in the allele frequencies along these environmental gradients were abrupt and occurred at a very narrow range of environmental variables. The few established functions between environmental gradients and the genetics of *Mytilus* spp. provide good starting conditions to understand how local environment would potentially shape species distributions and help us forecast future scenarios.

## Figures and Tables

**Table 1:** Genetic parameters of the 53 Mytilus mussel samples, including proportion of polymorphic loci, FIS values, loci not in HWE, observed and expected heterozygosity, minor allele frequency, average gene diversity per locus and average number of pairwise differences within populations.

Sample Name	Localisation	Country	Water Area	No. of Individuals	*P_O_*	*F_IS_*	Loci with HWE Departure	*H_O_*	*H_E_*	MAF	Average Gene Diversity over Loci	Average No. of Pairwise Differences within Population	Coordinates		Sample Collection
BNJ	Belmar, New Jersey	USA	Atlantic	32	46.30	**0.111**	2	0.233	0.275	0.086	0.122	5.23	40°11′13.56″ N	74°0′36.36″ W	2012
IRD * ^1, 2, 3, 4^	Indian River Inlet, Delaware,	USA	Atlantic	30	44.44	**0.108**	0	0.239	0.271	0.081	0.116	4.96	36°52′6.19″ N	75°58′2.16″ W	2012
KPV	Kiptopeke State Park, Virginia,	USA	Atlantic	31	44.44	**0.110**	1	0.229	0.259	0.074	0.106	4.67	37° 9′51.12″ N	75°59′29.40″ W	2012
MID	Mispillion Inlet, Delaware,	USA	Atlantic	30	44.44	**0.222**	1	0.215	0.282	0.084	0.123	5.16	38°56′42.00″ N	75°18′38.88″ W	2012
PNY	Point Lookout, New York	USA	Atlantic	33	48.15	0.059	2	0.215	0.243	0.079	0.106	4.73	40°35′34.80″ N	73°34′30.72″ W	2012
SNY	Stony Brook, New York	USA	Atlantic	30	48.15	0.058	0	0.236	0.254	0.081	0.110	4.95	40°55′15.96″ N	73°9′0.36″ W	2012
NWF	North coast of New Foundland	Canada	Atlantic	24	51.85	**0.109**	1	0.198	0.232	0.075	0.102	4.57	49°30′5.32″ N	55°41′44.21″ W	2012
PEI	Prince Edward Island	Canada	Atlantic	31	46.30	−0.008	1	0.226	0.250	0.078	0.093	4.35	46°26′11.10″ N	62°40′24.06″ W	2012
SNS	Ship Harbour, Nova Scotia	Canada	Atlantic	23	51.85	0.046	1	0.241	0.246	0.087	0.111	5.05	44°48′5.13″ N	62°50′13.55″ W	2012
KKA	Halifax, Nova Scotia	Canada	Atlantic	40	85.19	**0.120**	4	0.218	0.261	0.158	0.198	10.44	44°30′33.79″ N	63°29′24.91″ W	1996
PBAY	Placentia Bay, New Foundland	Canada	Atlantic	17	81.48	**0.535**	18	0.172	0.388	0.248	0.312	15.29	47° 2′40.05″ N	54°11′34.72″ W	2012
GLD ^1^	North-west Greenland, Maarmorilik, 17	Denmark	Atlantic	33	79.63	**0.328**	10	0.233	0.373	0.229	0.266	13.45	71°8′42.96″ N	51°16′31.99″ W	2012
GLL ^1^	North-west Greenland, Maarmorilik, L	Denmark	Atlantic	30	77.78	**0.493**	21	0.193	0.382	0.214	0.291	14.15	70°59′42.42″ N	52°16′41.37″ W	2012
SAV * ^2^	North Greenland, Savissivik	Denmark	Atlantic	27	40.38	**0.144**	1	0.243	0.284	0.082	0.089	4.59	76°1′5.26″ N	65°7′4.18″ W	2015
NUU	South-west Greenland, Nuuk	Denmark	Atlantic	25	37.04	0.003	1	0.296	0.336	0.081	0.113	4.90	64°10′24.36″ N	51°29′25.86″ W	2015
ISLB	Reykjavik	Iceland	Atlantic	29	42.59	0.083	2	0.282	0.322	0.087	0.127	5.74	64°8′59.03″ N	21°53′16.61″ W	1986
ISLR	Reykjavik	Iceland	Atlantic	30	46.30	−0.043	1	0.283	0.290	0.086	0.123	5.57	64°5′44.18″ N	21°56′48.83″ W	2004
SPI	Spitsbergen, Smerenburg	Norway	Atlantic	4	37.04	NA	NA	NA	NA	NA	NA	NA	79°38′46.94″ N	11°14′10.38″ E	2014
BRN	Bergen	Norway	Atlantic	36	88.89	**0.219**	7	0.281	0.379	0.262	0.325	15.71	60°23′25.65″ N	5°12′27.08″ E	2012
BODS	Bodø, Rundholmen	Norway	Atlantic	30	85.19	−0.004	0	0.194	0.201	0.106	0.156	7.54	67°17′0.61″ N	14°21′48.02″ E	2013
BODZ	Bodø, Rønvikleira	Norway	Atlantic	29	85.19	0.067	0	0.197	0.223	0.120	0.185	8.52	67°17′45.25″ N	14°23′49.53″ E	2013
TRO	Tromsø	Norway	Atlantic	29	64.81	0.068	1	0.198	0.213	0.089	0.123	5.92	69°35′27.68″ N	18°53′20.62″ E	2006
BAR	Barents Sea	Russia	Barents Sea	19	79.63	**0.562**	18	0.157	0.348	0.185	0.268	13.02	69°20′18″ N	34°01′28″ E	2004
KOL	Kola Bay, Abram Mys	Russia	Barents Sea	27	85.19	**0.444**	21	0.151	0.290	0.165	0.228	11.64	68°58′56.47″ N	33°1′36.08″ E	2014
DLZ	Dalnie Zelentsy, Yarnyshnaya	Russia	Barents Sea	30	83.33	**0.309**	2	0.135	0.201	0.108	0.162	7.35	69°5′16.56″ N	36°3′3.42″ E	2014
WSBS	White Sea Biological Station	Russia	White Sea	30	83.33	**0.278**	2	0.142	0.186	0.093	0.146	6.85	66°33′5.62″ N	33°6′50.58″ E	2014
CHU	Chupa Inlet, Kandalaksha Bay	Russia	White Sea	32	81.48	**0.465**	19	0.168	0.325	0.186	0.234	12.11	66°16′12.31″ N	33°4′12.93″ E	2014
ONE	Chupa	Russia	White Sea	28	53.70	**0.210**	4	0.202	0.247	0.089	0.128	5.59	66°15′51.67″ N	33°2′54.21″ E	1997
KER	Keret, Kandalaksha Bay	Russia	White Sea	33	83.33	**0.647**	26	0.135	0.357	0.207	0.297	14.36	66°17′22.66″ N	33°40′6.28″ E	2014
BIA	Białogóra	Poland	Baltic Sea	30	85.19	**0.098**	6	0.273	0.339	0.215	0.261	12.92	54°49′55.99″ N	17°57′9.02″ E	2014
BOR	Bornholm	Denmark	Baltic Sea	30	85.19	**0.075**	4	0.297	0.339	0.216	0.272	13.82	55°4′25.89″ N	14°43′56.56″ E	2013
KLA	Klaipeda	Lithuania	Baltic Sea	30	81.48	**0.094**	4	0.290	0.348	0.209	0.262	13.33	55°49′4″ N	20°30′2″ E	2013
STC	Stevns Klint	Denmark	Baltic Sea	28	87.04	**0.120**	5	0.305	0.374	0.241	0.306	15.03	55°16′50.25″ N	12°26′49.85″ E	2014
CIS	Cullivoe intertidal Shetland	Great Britain	Atlantic	33	46.30	−0.004	1	0.318	0.339	0.101	0.117	6.22	60°40′0.37″ N	0°56′40.85″ W	2012
SCO	Malage, Scotland	Great Britain	Atlantic	29	53.70	0.044	1	0.262	0.290	0.099	0.149	6.74	57°4′24.00″ N	5°47′24.00″ W	2014
LET	Loch Etive, Scotland	Great Britain	Atlantic	31	85.19	**0.527**	27	0.182	0.403	0.287	0.324	15.96	56°27′21.35″ N	5°18′26.62″ W	2008
OBA	Oban, Scotland	Great Britain	Atlantic	29	48.15	**0.127**	2	0.209	0.270	0.091	0.112	5.28	56°24′49.40″ N	5°28′23.00″ W	2014
IONA	Iona, Inner Hebrides, Scotland	Great Britain	Atlantic	29	53.70	**0.128**	3	0.274	0.318	0.111	0.147	7.30	56°19′52.72″ N	6°23′29.93″ W	2014
KRR	Kerrera, Inner Hebrides, Scotland	Great Britain	Atlantic	30	46.30	**0.128**	2	0.257	0.312	0.095	0.127	5.95	56°22′42.56″ N	5°33′17.14″ W	2014
STA	Staffa, Inner Hebrides, Scotland	Great Britain	Atlantic	30	48.15	**0.090**	0	0.310	0.347	0.111	0.144	7.06	56°26′9.98″ N	6°20′15.43″ W	2014
GBLO ^5^	Lowestoft	Great Britain	Atlantic	11	35.19	0.012	0	0.297	0.330	0.078	0.114	4.60	52°20′44.07″ N	1°45′27.63″ E	2000
LGF ^3, 4^	Lough Foyle	Ireland	Atlantic	28	48.15	0.062	1	0.232	0.266	0.088	0.096	5.14	55°5′35.50″ N	7°4′48.92″ W	2006
SAL	Saltö	Sweden	Atlantic	29	64.81	**0.084**	1	0.194	0.217	0.088	0.132	6.02	58°52′45.38″ N	11° 7′13.18″ E	2014
NLOO ^5^	Oosterschelde	Netherlands	Atlantic	17	42.59	−0.033	0	0.261	0.271	0.075	0.113	4.62	51°50′7.10″ N	3°49′18.21″ E	2000
MSM	Mont Saint-Michel	France	Atlantic	4	22.22	NA	NA	NA	NA	NA	NA	NA	48°39′0.06″ N	1°31′40.26″ W	2013
LOI * ^1^	Loire	France	Atlantic	30	50.00	**0.097**	0	0.229	0.251	0.088	0.108	5.08	47°14′43.83″ N	2°13′48.88″ W	2004
BID * ^1^	Bidasoa	Spain	Atlantic	30	50.00	0.033	1	0.331	0.345	0.123	0.159	7.75	43°21′38.71″ N	1°51′11.15″ W	2004
VIG	Vigo	Spain	Atlantic	30	53.70	0.081	2	0.285	0.318	0.125	0.161	7.65	42°13′54.12″ N	8°45′7.22″ W	2004
CAS	Cascais	Portugal	Atlantic	30	50.00	0.070	0	0.282	0.311	0.108	0.150	7.20	38°34′14.89″ N	9°19′8.95″ W	2013
CAM ^3, 4^	Camarinal	Spain	Atlantic	29	46.30	0.035	0	0.329	0.345	0.118	0.147	7.10	36° 3′30.09″ N	5°46′8.88″ W	2004
IMC	Torre Grande port	Italy	Mediterranean Sea	20	53.70	0.055	1	0.259	0.284	0.102	0.151	7.40	39°47′59.88″ N	8°31′9.72″ E	2004
NEA	Gulf of Naples	Italy	Mediterranean Sea	30	51.85	0.038	1	0.280	0.308	0.108	0.154	7.55	40°46′44.64″ N	14°5′28.20″ E	2014
AZO *	Azov sea	Russia	Azow Sea	30	48.15	0.004	1	0.277	0.279	0.083	0.132	6.56	45°43′51.71″ N	35°5′0.26″ E	1997

Values with *P* < 0.05 after Benjamini − Yekutieli correction are marked in bold; *P_O_*, % of polymorphic loci; *F_IS,_* inbreding coefficient; HWE, Hardy-Weinberg equilibrium; *H_O_*, observed heterozygosity; *H_E_*, expected heterozygosity; MAF, minor allele frequency; NA, not applicable; * reference sample. Samples used in other works: ^1^ [25]; ^2^ [26]; ^3^ [37]; ^4^ [38]; ^5^ [62].

**Table 2:** The spatial and temporal resolution, data range and units of environmental variables used in the modeling.

Variable	Unit	Temporal Resolution	Spatial Resolution	Data Range
Sea ice concentration	% coverage	monthly	0.25°	1986–1995
Cloud amount	percent total cloud amount	monthly	2°	1986–1995
Wind speed	m s^−1^	monthly	1°	1983–1993
Solar radiation	kJ m^−2^ day^−1^	monthly	0.04167°	1970–2000
Precipitation	mm	monthly	0.04167°	1970–2000
Water temperature	°C	monthly	0.25°	2005–2017
Salinity	psu	monthly	0.25°	2005–2017
Swell height	m	monthly	2°	1970–2015
Tide height	cm	monthly	0.0625°	modeled data
Chlorophyll-*a* concentration	mg m^−3^	monthly	0.04167°	1997–2017
Concentration of nitrates	µmol L^−2^	monthly	1°	1955–2012
Concentration of phosphates	µmol L^−3^	monthly	1°	1955–2012

**Table 3:** *M. trossulus* × *M. edulis* hybrid identification.

						Structure K = 2				NewHybrids			
Sample	Location		*HI*	*V_HI_*	*M. edulis*	*M. trossulus*	Hybrids	*M. edulis*	*M. trossulus*	F1 hybrids	F2 hybrids	tr_BAX	edu_BAX
North America					%	%	%	%	%	%	%	%	%
BNJ	USA	Atlantic	0.0254	0.0256	100	0	0	100	0	0	0	0	0
KPV	USA	Atlantic	0.0182	0.0210	100	0	0	100	0	0	0	0	0
MID	USA	Atlantic	0.0125	0.0194	100	0	0	100	0	0	0	0	0
PNY	USA	Atlantic	0.0114	0.0171	100	0	0	100	0	0	0	0	0
SNY	USA	Atlantic	0.0168	0.0229	100	0	0	100	0	0	0	0	0
NWF	Canada	Atlantic	0.0336	0.0281	100	0	0	100	0	0	0	0	0
PEI	Canada	Atlantic	0.0319	0.0320	100	0	0	100	0	0	0	0	0
SNS	Canada	Atlantic	0.0287	0.0282	100	0	0	100	0	0	0	0	0
KKA	Canada	Atlantic	0.8469	0.1996	2.5	80	17.5	0	77.5	17.5	0	2.5	2.5
PBAY	Canada	Atlantic	0.6132	0.4315	29.41	52.94	17.65	29.41	52.94	11.76	0	5.88	0
GLD	Greenland	Atlantic	0.6399	0.3781	21.21	48.48	30.30	21.21	48.48	24.24	0	6.06	0
GLL	Greenland	Atlantic	0.3728	0.4122	56.67	26.67	16.67	56.67	26.67	16.67	0	0	0
NUU	Greenland	Atlantic	0.0444	0.0306	100	0	0	100	0	0	0	0	0
North Russia													
ONE	Russia	White Sea	0.0202	0.0309	100	0	0	100	0	0	0	0	0
WSBS	Russia	White Sea	0.0531	0.1811	96.67	3.33	0	96.67	3.33	0	0	0	0
KER	Russia	White Sea	0.2804	0.4247	72.73	27.27	0	72.73	27.27	0	0	0	0
CHU	Russia	White Sea	0.7558	0.3486	15.63	71.88	12.50	15.63	71.88	9.38	0	3.13	0
DLZ	Russia	Barents Sea	0.0634	0.1958	93.33	3.33	3.33	93.33	3.33	3.33	0	0	0
BAR	Russia	Barents Sea	0.2503	0.4056	73.68	21.05	5.26	73.68	21.05	5.26	0	0	0
KOL	Russia	Barents Sea	0.8235	0.3100	11.11	85.19	3.70	11.11	77.78	3.70	0	7.41	0
Scandinavia and Loch Etive													
SAL	Sweden	Atlantic	0.0405	0.0465	100	0	0	96.55	0	0	0	0	3.45
TRO	Norway	Atlantic	0.0304	0.0295	100	0	0	96.55	0	0	0	0	3.45
BODS	Norway	Atlantic	0.0604	0.0921	96.67	0	3.33	80.00	0	0	3.33	0	16.67
BODZ	Norway	Atlantic	0.0752	0.1291	86.21	0	13.79	82.76	0	3.45	3.45	0	10.34
BRN	Norway	Atlantic	0.5867	0.2780	13.89	36.11	50.00	5.56	30.56	13.89	8.33	25.00	16.67
LET	Great Britain	Atlantic	0.5111	0.4374	38.71	48.39	12.90	38.71	41.94	12.90	0	6.45	0
Baltic Sea													
KLA	Lithuania	Baltic Sea	0.7255	0.0763	0	60	40	0	50	0	10	40	0
BIA	Poland	Baltic Sea	0.7010	0.0893	0	50	50	0	36,67	0	10	53.33	0
BOR	Denmark	Baltic Sea	0.7259	0.1152	0	63.33	36.67	0	43.33	0	6.67	50	0
STC	Denmark	Baltic Sea	0.6321	0.2004	3.57	46.43	50	3.57	32.14	0	14.29	39.29	10.71
reference M. edulis					0								
LOI	France	Atlantic	0.0073	0.0158	100	0	0	100	0	0	0	0	0
IRD	USA	Atlantic	0.0199	0.0255	100	0	0	100	0	0	0	0	0
reference M. trossulus													
SAV	Greenland	Atlantic	0.9629	0.0313	0	100	0	0	100	0	0	0	0

*HI*, hybrid index score; *V_HI_* ,variance of *HI*; tr_BAX, *M. trossulus* backcrosses; edu_BAX, *M. edulis* backcrosses.

## Supplementary Materials

The following are available online at https://www.mdpi.com/2073-4425/11/5/530/s1, Figure S1: The results of the Boosted Regression Tree technique (BRT) showing response functions of some allele frequencies of *Mytilus* spp. to the selected environmental variables (salinity, water temperature, ice cover and chlorophyll *a* concentration). The names of alleles are shown in the upper left corner of each figure and the respective allele is shown as a letter close to the regression line. The relative contribution of environmental variables in the BRT model is shown in brackets. Upward tick marks on the x-axis show the frequency of distribution of data along the respective environmental gradient. See the methods section for further information on environmental variables and BRT modeling. Table S1: SNP properties, genome location, references, GenBank annotation, substitution type and FST P-value associated with test for outlier status. Table S2: Allele frequencies of 54 SNP loci for 53 Mytilus spp. samples. Table S3: The environmental variables data for 53 *Mytilus* spp. samples. Table S4: Population specific FIS indices per polymorphic locus (absolute values). Table S5: FST distance matrix for 54 SNPs. P < 0.05 after FDR-BY correction is marked in bold. See Table 1.
