# Supplementary material for: Trans-Atlantic Distribution and Introgression as Inferred from Single Nucleotide Polymorphism: Mussels Mytilus and Environmental Factors
Source: Genes (Basel). 2020 May 10;11(5):530. doi: 10.3390/genes11050530 (PMC7288462; doi:10.3390/genes11050530)
Supplement: Supplementary file 1 [file genes-11-00530-s001.pdf]

Supplementary Materials

Trans-Atlantic Distribution and Introgression as Inferred from Single Nucleotide Polymorphism: Mussels *Mytilus* and Environmental Factors.

Roman Wenne [1], Małgorzata Zbawicka [1], Lis Bach [2], Petr Strelkov [3], Mikhail Gantsevich [4], Piotr Kukliński [1], Tomasz Kijewski [1], John H. McDonald [5], Kristil Kindem Sundsaasen [6], Mariann Árnyasi [6], Sigbjørn Lien [6], Ants Kaasik [7], Kristjan Herkül [7] and Jonne Kotta [7]

[1]Institute of Oceanology, Polish Academy of Sciences, 81-712 Sopot, Poland; RWenne@iopan.gda.pl; mzbawicka@iopan.pl; kuki@iopan.pl; tkijewski@iopan.gda.pl;
[2]Arctic Research Centre, Department of Bioscience, Aarhus University, 4000 Roskilde, Denmark; lb@bios.au.dk;
[3]Department of Ichthyology and Hydrobiology, St. Petersburg State University, 199034 St. Petersburg, Russia; p_strelkov@yahoo.com;
[4]Department of Invertebrate Zoology, Faculty of Biology, Moscow MV Lomonosov State University, 119234 Moscow, Russia; mgantsevich@gmail.com;
[5]Biology Department, Western Washington University, Bellingham, Washington, 98225, USA; mcdonald@udel.edu ;
[6]Centre for Integrative Genetics (CIGENE), Department of Animal and Aquacultural Sciences, Faculty of Biosciences, Norwegian University of Life Sciences, 1432 Ås, Norway; kristil.sundsaasen@nmbu.no (K.K.S.); mariann.arnyasi@nmbu.no (M.Á.); sigbjorn.lien@nmbu.no;
[7]Estonian Marine Institute, University of Tartu, 12619 Tallinn, Estonia; ants.kaasik@ut.ee; kristjan.herkul@ut.ee; jonne@sea.ee

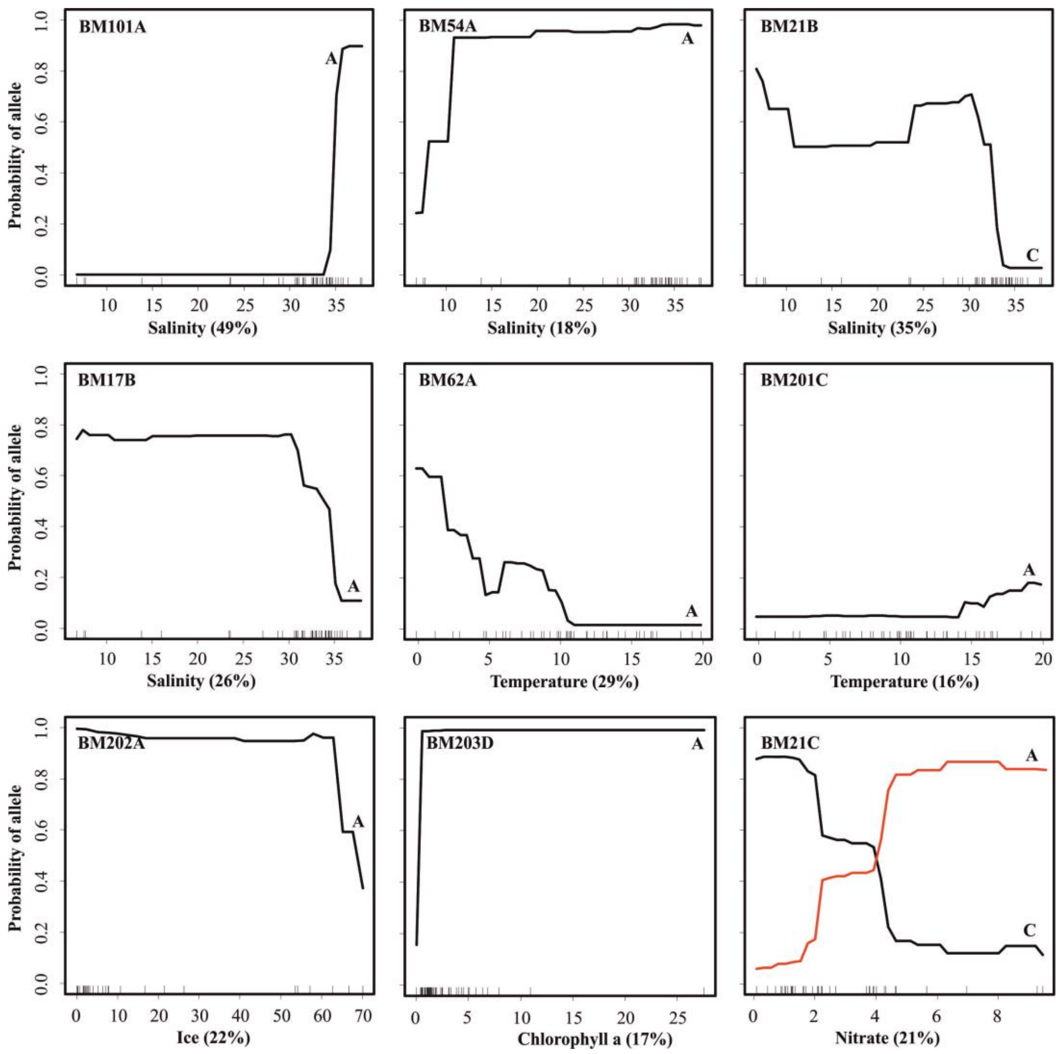

Fig. S1. The results of the Boosted Regression Tree technique (BRT) showing response functions of some allele frequencies of *Mytilus* spp. to the selected environmental variables (salinity, water temperature, ice cover and chlorophyll a concentration). The names of alleles are shown in

the upper left corner of each figure and the respective allele is shown as a letter close to the regression line. The relative contribution of environmental variables in the BRT model is shown in brackets. Upward tick-marks on the x-axis show the frequency of distribution of data along the respective environmental gradient. See the methods section for further information on environmental variables and BRT modelling.

Supplementary Table S1. SNP properties, genome location, references, GenBank annotation, substitution type and FST P-value associated with test for outlier status.

		GenBank					16pop			53pop			
Locus		Location	Annotation	Region	Substitution	Allele	H_O	F_{ST}		H_O	F_{ST}		Reference
BM101A		Ribosomal protein L7a	KT713378	coding	synon	A/T	0,4103	**0,8056**	*	0,2897	**0,8309**	*****	Wenne et al. 2016
BM102A		Proteasome subunit beta type-6-like	KT713379	coding	synon	C/T	0,4422	**0,0846**	*	0,4464	**0,0370**	*****	Wenne et al. 2016
BM103B	HI	Proteasome subunit beta type-5-like	KT713380	coding	nonsyn	A/G	0,2865	0,5651		0,3006	0,4879		Wenne et al. 2016
BM105A		UnKnown	KT713381	NA	NA	A/G	0,1776	0,2995		0,1424	0,3274		Wenne et al. 2016
BM106A		UnKnown	KT713382	NA	NA	A/G	0,3702	**0,6928**		NA	NA		Wenne et al. 2016
BM106B		UnKnown	KT713382	NA	NA	A/G	0,0518	0,2009		0,0180	0,1636		Wenne et al. 2016
BM10B	HI	Ribosomal protein S20	KJ871040	coding	synon	A/C	0,2127	0,5075		0,2575	0,4469		Zbawicka et al. 2014
BM113A	HI	Protein BTG1	KT713385	coding	synon	A/T	0,3324	**0,8036**	*	0,3407	**0,6877**	*****	Wenne et al. 2016
BM114B		UnKnown	KT713386	NA	NA	A/C	0,3503	**0,7592**		NA	NA		present study
BM115B		UnKnown	KT713384	NA	NA	A/G	0,3258	**0,7267**		NA	NA		Wenne et al. 2016
BM116A		ATP synthase beta subunit	KT713387	coding	synon	A/C	0,2801	**0,6648**		NA	NA		present study
BM118A		UnKnown	KT713388	NA	NA	A/G	0,0889	**0,1162**		0,0610	**0,0940**	*****	Wenne et al. 2016
BM11A	HI	Ribosomal protein L22	KJ871041	coding	synon	A/G	0,3370	**0,7877**		0,3502	**0,6488**	*****	Zbawicka et al. 2014
BM121A		Ribosomal protein L30	KT713389	coding	synon	A/T	0,1007	0,3325		0,0816	0,2981		present study
BM124A		UnKnown		NA	NA	C/T	0,3696	0,4946		NA	NA		present study
BM12A	HI	Ribosomal protein L23a	KJ871042	coding	synon	C/T	0,3255	**0,7169**		0,3508	**0,5505**	*****	Zbawicka et al. 2014
BM12C		Ribosomal protein L23a	KJ871042	coding	synon	C/T	0,4012	0,3132		0,3781	**0,2286**	*****	Wenne et al. 2016
BM144C		UnKnown	KT713377	NA	NA	C/T	0,3484	**0,8467**	*	NA	NA		present study
BM147A		UnKnown	KT713383	NA	NA	C/T	0,4735	**0,6590**		NA	NA		Wenne et al. 2016
BM151A		adhesive foot protein gene	HQ257471.1	coding	synon	G/T	NA	NA		0,0014	-0,0015		Gardner et al. 2016
BM16B		ribosomal protein L21	KJ871044	coding	synon	C/T	0,3338	**0,0112**	*	0,3038	**0,0247**	*****	Wenne et al. 2016

BM17B		Ribosomal protein L7a	KJ871045	coding	synon	A/G	0,5189	0,4572		0,4885	0,3877		Zbawicka et al. 2014
BM201B		*H4 histone* gene	AY267750.1	noncoding	NA	A/C	0,2600	0,5875		0,2519	0,4604		Zbawicka et al. 2012
BM201C		*H4 histone* gene	AY267750.1	coding	synon	G/T	0,3555	0,5245		0,2550	0,4313		Zbawicka et al. 2012
BM202A		*H3 histone* gene	AY267749.1	noncoding	NA	A/C	0,3265	**0,8057**	*	0,3388	**0,6911**	*****	Zbawicka et al. 2012
BM202B	HI	*H3 histone* gene	AY267749.1	coding	synon	A/T	0,3279	**0,8391**	*	0,3390	**0,7039**	*****	Zbawicka et al. 2012
BM203B		*H2B histone* gene	AY267742.1	noncoding	NA	C/T	0,3363	0,5064		0,2378	0,4179		Zbawicka et al. 2012
BM203C		*H2B histone* gene	AY267742.1	noncoding	NA	C/T	0,3822	**0,1677**		0,4138	**0,1486**	*****	Zbawicka et al. 2012
BM203D	HI	*H2A histone* gene	AY267757.1	noncoding	NA	A/T	0,3290	**0,8232**	*	0,3403	**0,6992**	*****	Zbawicka et al. 2012
BM204A		*p53*	DQ865151	coding	synon	C/T	0,2756	0,2638		0,1968	**0,2572**		Zbawicka et al. 2012
BM206A		*hsp70 gene*	AJ783713	coding	synon	A/G	0,4235	**0,1654**	*	NA	NA		Zbawicka et al. 2012
BM206B		*hsp70* gene	AJ783713	coding	nonsyn	A/G	0,0067	0,0182		NA	NA		Zbawicka et al. 2012
BM21B		qm-like protein	KJ871047	coding	nonsyn	C/G	0,3889	0,5414		0,4912	**0,6100**	*****	Zbawicka et al. 2014
BM21C		qm-like protein	KJ871047	coding	synon	A/C/T	0,5624	0,3675		0,5112	0,4036		Zbawicka et al. 2014
BM26B	HI	UnKnown13	KJ871050	NA	NA	A/T	0,3595	0,5970		0,3565	**0,5765**	*****	Zbawicka et al. 2014
BM2G	HI	UnKnown05	KJ871032	coding	synon	G/T	0,2263	0,6002		0,2688	**0,5553**	*****	Zbawicka et al. 2014
BM30A	HI	Ribosomal protein l17	KJ871052	coding	synon	A/G	0,2620	0,5888		0,2715	0,4812		Zbawicka et al. 2014
BM30C		Ribosomal protein l17	KJ871052	coding	synon	A/T	0,2074	0,4279		0,2309	0,3724		Zbawicka et al. 2014
BM32A		Ubiquinol-cytochrome c reductase subunit 6	KT713371	coding	synon	A/G	0,4738	**0,0458**	*	0,4444	**0,0490**	*****	Wenne et al. 2016
BM33B		Cytochrome c oxidase subunit IV	KJ871054	coding	synon	A/T	0,1521	0,3244		0,1783	0,3301		Zbawicka et al. 2014
BM35C		Ribosomal protein L7	KJ871055	coding	synon	A/T	0,5012	**0,1411**	*	0,4923	**0,1287**	*****	Wenne et al. 2016
BM35D		Ribosomal protein L7	KJ871055	coding	synon	A/G	0,2175	0,2880		0,2238	0,2962		Zbawicka et al. 2014
BM36F	HI	ribosomal protein S3a	KT713373	coding	synon	A/C	0,3019	0,3690		0,3598	0,3873		Wenne et al. 2016
BM38B		ribosomal protein S8e	KT713368	coding	synon	A/G	0,2936	0,2658		0,3267	0,3181		Wenne et al. 2016
BM44B		ubiquitin/ribosomal protein S27a	KJ871057	coding	synon	A/G	0,2513	0,5068		0,2301	0,4018		Zbawicka et al. 2014
BM50B		CoA-binding protein	KJ871059	coding	synon	A/G	0,0409	0,0584		0,0437	**0,0489**	*****	Zbawicka et al. 2014
BM54A	HI	ETC_C1_NDUFA4	KJ871060	coding	synon	A/G	0,3642	**0,8418**	*	0,3492	**0,6930**	*****	Zbawicka et al. 2014
BM57A		NADH-ubiquinone_oxidoreductase	KT713374	coding	nonsyn	C/T	0,3089	0,5098		0,2004	**0,5234**		Wenne et al. 2016
BM57D		NADH-ubiquinone_oxidoreductase	KT713374	coding	synon	A/C	0,2971	0,4706		0,1864	0,4487		Wenne et al. 2016
BM5B		Ribosomal protein S6e	KJ871035	coding	synon	A/G	0,0593	**0,0531**		0,0311	0,0389		Wenne et al. 2016
BM5D		Ribosomal protein S6e	KJ871035	coding	synon	C/T	0,4350	**0,1086**	*	0,4415	**0,0726**	*****	Zbawicka et al. 2014
BM60A		UnKnown08	KJ871063	coding	synon	A/G	0,1711	0,3419		0,1592	0,2635		Zbawicka et al. 2014
BM61A		Ribosomal_L1	KT713375	coding	synon	C/T	0,4254	**0,1122**	*	0,4763	**0,1501**	*****	Wenne et al. 2016
BM62A	HI	Ribosomal L13e	KJ871064	coding	synon	A/G	0,4097	**0,6830**		0,4130	**0,5894**	*****	Zbawicka et al. 2014
BM64A	HI	Ribosomal protein L35	KJ871065	coding	synon	C/T	0,2705	0,6358		0,3100	**0,5677**	*****	Zbawicka et al. 2014
BM67C		Ribosomal protein S6e	KJ871066	coding	synon	A/T	0,5134	0,4884		0,4802	0,3630		Wenne et al. 2016
BM6C		EFG_N	KJ871036	coding	synon	C/T	0,3741	**0,1799**		0,3934	**0,1466**	*****	Zbawicka et al. 2014
BM74A		TMA7_Translation_machinery_associated_TMA7	KT713372	coding	synon	A/T	0,0903	**0,0517**		NA	NA		present study
BM75C		UBA_UBA-TS-N_domain	KT713370	coding	synon	C/G	0,0668	0,1285		0,0622	**0,1337**		Wenne et al. 2016
BM78B		UnKnown12	KJ871069	coding	synon	A/G	0,3478	0,4654		0,2690	0,3536		Zbawicka et al. 2014

BM83A		UnKnown14	KJ871071	NA	NA	A/C	0,4675	**0,6667**		NA	NA		Zbawicka et al. 2014
BM8E	HI	Ribosomal protein L3	KJ871038	coding	synon	A/G	0,2817	0,5742		0,3254	**0,5350**	*****	Zbawicka et al. 2014
BM92B	HI	UnKnown06	KJ871074	coding	synon	A/T	0,3097	**0,7663**		0,3313	**0,6598**	*****	Zbawicka et al. 2014
BM96B		UnKnown09	KJ871076	coding	synon	C/T	0,2838	0,6420		NA	NA		Zbawicka et al. 2014
BM9B		Ribosomal protein S2	KJ871039	coding	synon	A/G	0,4802	**0,0844**	*	0,4993	**0,0648**	*****	Zbawicka et al. 2014
BM9C		Ribosomal protein S2	KJ871039	coding	synon	A/C/T	0,5687	**0,2296**		0,5365	**0,1615**	*****	Zbawicka et al. 2014

Values with P < 0.05 are marked in bold; *, values with P < 0.05 after Benjamini–Yekutieli correction; $F_{IS,}$ inbreding coefficient; $H_{O,}$ observed heterozygosity; NA, not applicable; HI, SNPs used to hybrid index score calculation.

Supplementary Table S2. Allele frequencies of 54 SNP loci for 53 Mytilus. spp.samples

Locus	Allele	BNJ	IRD	KPV	MID	PNY	SNY	NWF	PEI	SNS	KKA	PBAY	GLD	GLL	SAV	NUU	ISLB	ISLR	SPI	BRN	BODS	BODZ	TRO	BAR	KOL	DLZ	WSBS	CHU	ONE	KER	BIA	BOR	KLA	STC	CIS	SCO	LET	OBA	IONA	KRR	STA	GBLO	LGF	SAL	NLOO	MSM	LOI	BID	VIG	CAS	CAM	IMC	NEA	AZO
BM101A	A	0	0	0	0	0	0	0	0	0	0	0	0	0	0	0	0	0	0.125	0	0.01667	0.05172	0.03448	0	0.03704	0	0	0	0	0	0	0	0	0	0.21667	0.14815	0	0	0.28846	0.11538	0.42857	0	0.04	0	0	0	0	0.95	0.95	0.96667	1	0.975	1	1
BM101A	T	1	1	1	1	1	1	1	1	1	1	1	1	1	1	1	1	1	0.875	1	0.98333	0.94828	0.96552	1	0.96296	1	1	1	1	1	1	1	1	1	0.78333	0.85185	1	1	0.71154	0.88462	0.57143	1	0.96	1	1	1	1	0.05	0.05	0.03333	0	0.025	0	0
BM102A	C	0.4375	0.46667	0.40323	0.51667	0.35938	0.37931	0.41667	0.35185	0.38636	0.02778	0.125	0.47368	0.36364	0	0.44	0.43103	0.48276	0.375	0.46552	0.34483	0.41379	0.5	0.3125	0.16667	0.42593	0.34483	0.12	0.44444	0.33871	0.48077	0.40909	0.1875	0.40476	0.29032	0.39286	0.325	0.35714	0.40741	0.46667	0.38462	0.40909	0.34	0.2931	0.34375	0.75	0.44231	0.27778	0.32143	0.26667	0.33929	0.175	0.12963	0.13793
BM102A	T	0.5625	0.53333	0.59677	0.48333	0.64062	0.62069	0.58333	0.64815	0.61364	0.97222	0.875	0.52632	0.63636	1	0.56	0.56897	0.51724	0.625	0.53448	0.65517	0.58621	0.5	0.6875	0.83333	0.57407	0.65517	0.88	0.55556	0.66129	0.51923	0.59091	0.8125	0.59524	0.70968	0.60714	0.675	0.64286	0.59259	0.53333	0.61538	0.59091	0.66	0.7069	0.65625	0.25	0.55769	0.72222	0.67857	0.73333	0.66071	0.825	0.87037	0.86207
BM103B	A	1	1	1	1	1	1	1	1	1	0.32051	0.5	0.58333	0.8	0.21154	1	1	1	1	0.42593	0.9	0.93103	1	0.84211	0.34615	0.93333	0.88333	0.375	0.83333	0.65152	0.33333	0.42	0.18333	0.30769	1	1	0.53333	1	1	1	1	1	1	0.89655	1	1	1	1	1	1	1	1	1	1
BM103B	G	0	0	0	0	0	0	0	0	0	0.67949	0.5	0.41667	0.2	0.78846	0	0	0	0	0.57407	0.1	0.06897	0	0.15789	0.65385	0.06667	0.11667	0.625	0.16667	0.34848	0.66667	0.58	0.81667	0.69231	0	0	0.46667	0	0	0	0	0	0	0.10345	0	0	0	0	0	0	0	0	0	0
BM105A	A	1	1	1	1	1	1	1	1	1	1	1	1	1	1	1	1	1	1	1	0.96667	0.94828	0.98276	1	1	1	1	1	1	1	1	1	1	1	0.86364	0.94828	0.98333	0.93103	0.82759	0.93333	0.8	0.95455	0.98214	1	0.97059	1	0.9	0.51724	0.55	0.6	0.43103	0.525	0.46667	0.93103
BM105A	G	0	0	0	0	0	0	0	0	0	0	0	0	0	0	0	0	0	0	0	0.03333	0.05172	0.01724	0	0	0	0	0	0	0	0	0	0	0	0.13636	0.05172	0.01667	0.06897	0.17241	0.06667	0.2	0.04545	0.01786	0	0.02941	0	0.1	0.48276	0.45	0.4	0.56897	0.475	0.53333	0.06897
BM106B	A	1	1	1	1	1	1	1	1	1	0.98529	1	1	1	1	1	1	1	1	1	1	1	1	1	1	1	1	1	1	1	1	1	1	1	1	1	1	1	1	1	1	1	1	1	1	1	1	1	0.93333	1	1	0.85	0.73333	1
BM106B	G	0	0	0	0	0	0	0	0	0	0.01471	0	0	0	0	0	0	0	0	0	0	0	0	0	0	0	0	0	0	0	0	0	0	0	0	0	0	0	0	0	0	0	0	0	0	0	0	0	0.06667	0	0	0.15	0.26667	0
BM10B	A	1	1	1	1	1	1	1	1	1	0.28947	0.47059	0.42188	0.68333	0.19231	1	1	1	1	0.55556	0.95	0.98276	0.98276	0.76316	0.37037	0.95	0.96667	0.35	1	0.77273	0.65517	0.4	0.58333	0.57143	1	1	0.65517	1	1	1	1	1	1	1	1	1	1	1	1	1	1	1	1	1
BM10B	C	0	0	0	0	0	0	0	0	0	0.71053	0.52941	0.57812	0.31667	0.80769	0	0	0	0	0.44444	0.05	0.01724	0.01724	0.23684	0.62963	0.05	0.03333	0.65	0	0.22727	0.34483	0.6	0.41667	0.42857	0	0	0.34483	0	0	0	0	0	0	0	0	0	0	0	0	0	0	0	0	0
BM113A	A	1	1	1	1	1	1	1	1	1	0.15	0.38235	0.36364	0.63333	0.01923	1	1	1	0.875	0.33333	0.93333	0.96552	0.98214	0.76316	0.12963	0.93333	0.96667	0.22581	1	0.74242	0.08333	0.1	0.05	0.19643	1	1	0.48333	1	1	1	1	1	1	0.96552	1	1	1	1	1	1	1	1	1	1
BM113A	T	0	0	0	0	0	0	0	0	0	0.85	0.61765	0.63636	0.36667	0.98077	0	0	0	0.125	0.66667	0.06667	0.03448	0.01786	0.23684	0.87037	0.06667	0.03333	0.77419	0	0.25758	0.91667	0.9	0.95	0.80357	0	0	0.51667	0	0	0	0	0	0	0.03448	0	0	0	0	0	0	0	0	0	0
BM118A	A	0	0	0	0	0	0	0	0	0	0.025	0	0	0	0	0	0.06897	0	0	0.01389	0.01667	0	0.03448	0.02632	0	0	0.01667	0	0.03571	0	0	0	0	0	0.03125	0.06897	0	0	0.03448	0	0.05	0	0.05556	0	0.03571	0	0.01667	0.18966	0.18333	0.08929	0.09259	0.275	0.21667	0.08333
BM118A	G	1	1	1	1	1	1	1	1	1	0.975	1	1	1	1	1	0.93103	1	1	0.98611	0.98333	1	0.96552	0.97368	1	1	0.98333	1	0.96429	1	1	1	1	1	0.96875	0.93103	1	1	0.96552	1	0.95	1	0.94444	1	0.96429	1	0.98333	0.81034	0.81667	0.91071	0.90741	0.725	0.78333	0.91667
BM11A	A	0.95312	0.98333	0.96774	1	0.98485	0.98333	0.91667	0.95161	0.95652	0.10256	0.35294	0.34848	0.61667	0	1	1	1	1	0.34722	0.96667	0.93103	0.96552	0.76316	0.16667	0.94828	0.93333	0.15625	0.98214	0.72727	0.1	0.15	0.1	0.23214	1	1	0.48333	1	1	1	1	1	1	0.93103	1	1	0.98333	1	1	1	1	1	1	0.98333
BM11A	G	0.04688	0.01667	0.03226	0	0.01515	0.01667	0.08333	0.04839	0.04348	0.89744	0.64706	0.65152	0.38333	1	0	0	0	0	0.65278	0.03333	0.06897	0.03448	0.23684	0.83333	0.05172	0.06667	0.84375	0.01786	0.27273	0.9	0.85	0.9	0.76786	0	0	0.51667	0	0	0	0	0	0	0.06897	0	0	0.01667	0	0	0	0	0	0	0.01667
BM121A	A	0	0	0	0	0	0	0	0	0	0.0125	0	0	0	0	0	0	0	0	0.31944	0.01667	0.06897	0	0	0.05556	0	0	0	0	0	0.44643	0.36667	0.4	0.28846	0	0	0.03226	0	0	0	0	0	0	0.03448	0	0	0	0	0	0	0	0	0	0
BM121A	T	1	1	1	1	1	1	1	1	1	0.9875	1	1	1	1	1	1	1	1	0.68056	0.98333	0.93103	1	1	0.94444	1	1	1	1	1	0.55357	0.63333	0.6	0.71154	1	1	0.96774	1	1	1	1	1	1	0.96552	1	1	1	1	1	1	1	1	1	1
BM12A	C	0.79688	0.81667	0.91935	0.93333	0.92424	0.95	0.97826	0.96667	0.97727	0.2	0.44118	0.37879	0.58333	0	1	1	0.98333	0.875	0.52778	0.96667	0.87931	0.96552	0.76316	0.16667	0.93333	0.95	0.20968	0.98077	0.75758	0.13333	0.15	0.21667	0.24074	1	1	0.45161	1	1	1	1	0.95455	0.98214	0.89655	0.97059	1	0.96667	1	1	0.98333	0.98276	1	1	1
BM12A	T	0.20312	0.18333	0.08065	0.06667	0.07576	0.05	0.02174	0.03333	0.02273	0.8	0.55882	0.62121	0.41667	1	0	0	0.01667	0.125	0.47222	0.03333	0.12069	0.03448	0.23684	0.83333	0.06667	0.05	0.79032	0.01923	0.24242	0.86667	0.85	0.78333	0.75926	0	0	0.54839	0	0	0	0	0.04545	0.01786	0.10345	0.02941	0	0.03333	0	0	0.01667	0.01724	0	0	0
BM12C	C	0.25806	0.21429	0.09677	0.15	0.10345	0.05172	0.06522	0.03226	0.04545	0.56579	0.32353	0.5	0.44643	0.44444	0	0.08621	0.13333	0.25	0.26786	0.2	0.2069	0.08929	0.23684	0.69231	0.19643	0.1	0.61667	0.03571	0.19697	0.30769	0.75862	0.625	0.28125	0.13333	0.08621	0.44444	0.14583	0.09259	0.03448	0.08621	0.05	0.08929	0.04	0.1875	0	0.17241	0.10345	0.21667	0.13333	0.18966	0.65	0.55172	0.71667
BM12C	T	0.74194	0.78571	0.90323	0.85	0.89655	0.94828	0.93478	0.96774	0.95455	0.43421	0.67647	0.5	0.55357	0.55556	1	0.91379	0.86667	0.75	0.73214	0.8	0.7931	0.91071	0.76316	0.30769	0.80357	0.9	0.38333	0.96429	0.80303	0.69231	0.24138	0.375	0.71875	0.86667	0.91379	0.55556	0.85417	0.90741	0.96552	0.91379	0.95	0.91071	0.96	0.8125	1	0.82759	0.89655	0.78333	0.86667	0.81034	0.35	0.44828	0.28333
BM151A	G	1	1	1	1	1	1	1	1	1	1	1	1	1	1	1	1	1	1	1	1	1	1	1	1	1	1	1	1	1	1	1	1	1	1	0.98276	1	1	1	1	1	1	0.98148	1	1	1	1	1	1	1	1	1	1	1
BM151A	T	0	0	0	0	0	0	0	0	0	0	0	0	0	0	0	0	0	0	0	0	0	0	0	0	0	0	0	0	0	0	0	0	0	0	0.01724	0	0	0	0	0	0	0.01852	0	0	0	0	0	0	0	0	0	0	0
BM16B	A	0.73438	0.83333	0.87097	0.71667	0.75	0.88333	0.95652	0.8871	0.88095	0.925	0.94118	0.89394	0.85	1	0.76	0.82759	0.78333	1	0.76389	0.82759	0.86207	0.69643	0.92105	0.94231	0.76667	0.8	1	0.75	0.80303	0.78333	0.75	0.9	0.78571	0.72727	0.68966	0.87097	0.74138	0.81034	0.71667	0.84483	0.81818	0.75926	0.82759	0.85294	1	0.81667	0.75	0.75	0.76667	0.7931	0.7	0.75	0.66667
BM16B	G	0.26562	0.16667	0.12903	0.28333	0.25	0.11667	0.04348	0.1129	0.11905	0.075	0.05882	0.10606	0.15	0	0.24	0.17241	0.21667	0	0.23611	0.17241	0.13793	0.30357	0.07895	0.05769	0.23333	0.2	0	0.25	0.19697	0.21667	0.25	0.1	0.21429	0.27273	0.31034	0.12903	0.25862	0.18966	0.28333	0.15517	0.18182	0.24074	0.17241	0.14706	0	0.18333	0.25	0.25	0.23333	0.2069	0.3	0.25	0.33333
BM17B	A	0.89062	0.86667	0.83871	0.85	0.84848	0.78333	0.91304	1	0.93478	0.26562	0.42857	0.55357	0.66667	0	0.88	0.85185	0.86667	0.625	0.54412	0.75	0.69643	0.75862	0.47368	0.14583	0.84483	0.85	0.31034	0.96429	0.6875	0.78125	0.8	0.83333	0.88462	0.4375	0.62069	0.48214	0.63793	0.48214	0.58333	0.39286	0.63636	0.67857	0.93103	0.67647	0.5	0.6	0.06897	0.03333	0	0	0.025	0	0
BM17B	G	0.10938	0.13333	0.16129	0.15	0.15152	0.21667	0.08696	0	0.06522	0.73438	0.57143	0.44643	0.33333	1	0.12	0.14815	0.13333	0.375	0.45588	0.25	0.30357	0.24138	0.52632	0.85417	0.15517	0.15	0.68966	0.03571	0.3125	0.21875	0.2	0.16667	0.11538	0.5625	0.37931	0.51786	0.36207	0.51786	0.41667	0.60714	0.36364	0.32143	0.06897	0.32353	0.5	0.4	0.93103	0.96667	1	1	0.975	1	1
BM201B	A	0	0	0	0	0	0	0	0	0	0.63235	0.40625	0.375	0.18333	0.71154	0	0	0	0	0.37143	0	0	0	0.17647	0.61538	0.01724	0.03333	0.53448	0	0.20312	0.65	0.58621	0.66667	0.66071	0	0	0.41071	0	0	0	0	0	0	0	0	0	0	0	0	0	0	0	0	0
BM201B	C	1	1	1	1	1	1	1	1	1	0.36765	0.59375	0.625	0.81667	0.28846	1	1	1	1	0.62857	1	1	1	0.82353	0.38462	0.98276	0.96667	0.46552	1	0.79688	0.35	0.41379	0.33333	0.33929	1	1	0.58929	1	1	1	1	1	1	1	1	1	1	1	1	1	1	1	1	1
BM201C	G	0.03125	0.01667	0.06452	0.15	0.07576	0.01786	0.13043	0.06452	0.06522	0	0.06667	0	0	0	0.02	0.09259	0.03333	0.25	0.01389	0.06667	0.03448	0.10345	0	0	0.05	0.05	0.03125	0.07143	0.07812	0.01724	0.01667	0	0.01786	0.19355	0.125	0	0.06897	0.3	0.1	0.31034	0	0.07407	0.01724	0.02941	0	0	0.65	0.61538	0.72414	0.58929	0.75	0.85185	0.81667
BM201C	T	0.96875	0.98333	0.93548	0.85	0.92424	0.98214	0.86957	0.93548	0.93478	1	0.93333	1	1	1	0.98	0.90741	0.96667	0.75	0.98611	0.93333	0.96552	0.89655	1	1	0.95	0.95	0.96875	0.92857	0.92188	0.98276	0.98333	1	0.98214	0.80645	0.875	1	0.93103	0.7	0.9	0.68966	1	0.92593	0.98276	0.97059	1	1	0.35	0.38462	0.27586	0.41071	0.25	0.14815	0.18333
BM202A	A	1	1	1	1	1	1	1	1	1	0.09211	0.45833	0.32692	0.64815	0	1	1	1	1	0.4	0.98333	0.94643	1	0.77778	0.15385	0.96667	0.96667	0.2	1	0.72727	0.19231	0.12	0.08333	0.22222	1	1	0.51667	1	1	1	1	1	1	1	1	1	1	1	1	1	1	1	1	1
BM202A	C	0	0	0	0	0	0	0	0	0	0.90789	0.54167	0.67308	0.35185	1	0	0	0	0	0.6	0.01667	0.05357	0	0.22222	0.84615	0.03333	0.03333	0.8	0	0.27273	0.80769	0.88	0.91667	0.77778	0	0	0.48333	0	0	0	0	0	0	0	0	0	0	0	0	0	0	0	0	0
BM202B	A	0	0	0	0	0	0	0	0	0	0.8875	0.625	0.66071	0.35	1	0	0	0	0	0.65625	0.01724	0.03704	0	0.23684	0.85185	0.03448	0.03333	0.78125	0	0.27273	0.94	0.87037	0.92593	0.77778	0	0	0.51724	0	0	0	0	0	0	0	0	0	0	0	0	0	0	0	0	0
BM202B	T	1	1	1	1	1	1	1	1	1	0.1125	0.375	0.33929	0.65	0	1	1	1	1	0.34375	0.98276	0.96296	1	0.76316	0.14815	0.96552	0.96667	0.21875	1	0.72727	0.06	0.12963	0.07407	0.22222	1	1	0.48276	1	1	1	1	1	1	1	1	1	1	1	1	1	1	1	1	1
BM203B	C	0.98387	0.98333	0.96552	0.86207	0.96667	0.94828	0.875	0.93548	0.93478	1	0.875	1	1	0.98148	1	0.90741	0.96667	0.75	0.98611	0.94828	0.96552	0.92857	1	1	0.94828	0.98276	0.98387	0.92857	0.96774	0.96667	0.98333	1	0.98214	0.88333	0.87037	1	0.94828	0.71429	0.93333	0.7069	1	0.91071	0.98276	1	1	1	0.46296	0.38889	0.32759	0.4	0.275	0.24138	0.18333
BM203B	T	0.01613	0.01667	0.03448	0.13793	0.03333	0.05172	0.125	0.06452	0.06522	0	0.125	0	0	0.01852	0	0.09259	0.03333	0.25	0.01389	0.05172	0.03448	0.07143	0	0	0.05172	0.01724	0.01613	0.07143	0.03226	0.03333	0.01667	0	0.01786	0.11667	0.12963	0	0.05172	0.28571	0.06667	0.2931	0	0.08929	0.01724	0	0	0	0.53704	0.61111	0.67241	0.6	0.725	0.75862	0.81667
BM203C	A	0.33333	0.67241	0.46296	0.48333	0.42857	0.4375	0.30952	0.36538	0.47222	0.0875	0.03125	0.125	0.26786	0	0.39474	0.32	0.38462	0.125	0.13235	0.5	0.51786	0.36	0.41667	0.07692	0.33929	0.43182	0.06452	0.54167	0.2963	0.05172	0.03448	0.03704	0.14	0.38	0.42593	0.2	0.47826	0.31818	0.52174	0.28	0.4	0.40909	0.30435	0.46667	0.375	0.66667	0.16	0.17241	0.1	0.12963	0	0.01667	0
BM203C	G	0.66667	0.32759	0.53704	0.51667	0.57143	0.5625	0.69048	0.63462	0.52778	0.9125	0.96875	0.875	0.73214	1	0.60526	0.68	0.61538	0.875	0.86765	0.5	0.48214	0.64	0.58333	0.92308	0.66071	0.56818	0.93548	0.45833	0.7037	0.94828	0.96552	0.96296	0.86	0.62	0.57407	0.8	0.52174	0.68182	0.47826	0.72	0.6	0.59091	0.69565	0.53333	0.625	0.33333	0.84	0.82759	0.9	0.87037	1	0.98333	1
BM203D	A	1	1	1	1	1	1	1	1	1	0.1125	0.375	0.32759	0.65	0	1	1	1	1	0.34375	0.98276	0.94643	1	0.76316	0.13462	0.96552	0.96667	0.20968	1	0.72727	0.10714	0.12963	0.08929	0.22222	1	1	0.48276	1	1	1	1	1	1	1	1	1	1	1	1	1	1	1	1	1
BM203D	T	0	0	0	0	0	0	0	0	0	0.8875	0.625	0.67241	0.35	1	0	0	0	0	0.65625	0.01724	0.05357	0	0.23684	0.86538	0.03448	0.03333	0.79032	0	0.27273	0.89286	0.87037	0.91071	0.77778	0	0	0.51724	0	0	0	0	0	0	0	0	0	0	0	0	0	0	0	0	0
BM204A	C	0.0625	0.08333	0.01613	0.01667	0.01515	0.03333	0.04167	0.06452	0.08696	0	0.02941	0.01515	0.05	0	0.06	0.17241	0.15	0	0.01389	0.08333	0.01724	0.03448	0	0.01852	0.01724	0	0	0.01786	0.01515	0	0	0.01667	0.01786	0.12121	0.18966	0.03226	0.05172	0.15517	0.15	0.21667	0	0.05357	0.01724	0	0.125	0.03448	0.46552	0.5	0.51724	0.35714	0.45	0.51667	0.6
BM204A	T	0.9375	0.91667	0.98387	0.98333	0.98485	0.96667	0.95833	0.93548	0.91304	1	0.97059	0.98485	0.95	1	0.94	0.82759	0.85	1	0.98611	0.91667	0.98276	0.96552	1	0.98148	0.98276	1	1	0.98214	0.98485	1	1	0.98333	0.98214	0.87879	0.81034	0.96774	0.94828	0.84483	0.85	0.78333	1	0.94643	0.98276	1	0.875	0.96552	0.53448	0.5	0.48276	0.64286	0.55	0.48333	0.4
BM21B	C	0.92188	0.95	0.90323	0.95	0.90909	0.83333	0.86364	0.96774	0.95455	0.9875	0.97059	0.9697	0.8	1	0.82	0.25862	0.36667	0.375	0.54167	0.18966	0.2931	0.08621	0.34211	0.85185	0.13333	0.25	0.82812	0.26786	0.43939	0.36667	0.51667	0.41667	0.32143	0	0	0.53226	0	0	0	0	0	0	0.08929	0	0	0	0	0	0	0	0	0	0
BM21B	G	0.07812	0.05	0.09677	0.05	0.09091	0.16667	0.13636	0.03226	0.04545	0.0125	0.02941	0.0303	0.2	0	0.18	0.74138	0.63333	0.625	0.45833	0.81034	0.7069	0.91379	0.65789	0.14815	0.86667	0.75	0.17188	0.73214	0.56061	0.63333	0.48333	0.58333	0.67857	1	1	0.46774	1	1	1	1	1	1	0.91071	1	1	1	1	1	1	1	1	1	1
BM21C	A	0.92188	0.95	0.90323	0.95	0.90909	0.83333	0.875	0.96774	0.95652	0.9875	0.97059	0.9697	0.81034	1	0.82	0.25862	0.36667	0.375	0.54167	0.24138	0.31034	0.18966	0.36842	0.85185	0.13793	0.26667	0.82812	0.26786	0.4375	0.41667	0.51667	0.46429	0.35714	0.34375	0.46552	0.53226	0.01724	0.62069	0.5	0.76667	0	0.03704	0.16071	0.02941	0	0.03333	0.77586	0.9	0.86667	0.93103	0.875	0.8	0.81667
BM21C	C	0.07812	0.05	0.08065	0.05	0.09091	0.15	0.125	0.03226	0.04348	0.0125	0.02941	0.0303	0.18966	0	0.18	0.68966	0.63333	0.5	0.43056	0.72414	0.63793	0.77586	0.63158	0.14815	0.82759	0.73333	0.17188	0.73214	0.5625	0.58333	0.45	0.53571	0.64286	0.64062	0.41379	0.46774	0.94828	0.22414	0.46667	0.06667	1	0.94444	0.82143	0.85294	1	0.93333	0.03448	0.01667	0.01667	0.01724	0	0	0
BM21C	T	0	0	0.01613	0	0	0.01667	0	0	0	0	0	0	0	0	0	0.05172	0	0.125	0.02778	0.03448	0.05172	0.03448	0	0	0.03448	0	0	0	0	0	0.03333	0	0	0.01562	0.12069	0	0.03448	0.15517	0.03333	0.16667	0	0.01852	0.01786	0.11765	0	0.03333	0.18966	0.08333	0.11667	0.05172	0.125	0.2	0.18333
BM26B	A	1	1	1	1	0.98485	0.98333	0.95833	1	0.95652	0.10256	0.38235	0.35938	0.65	0	1	0.96552	1	1	0.31429	0.93333	0.94828	0.93103	0.77778	0.14815	0.93333	0.91667	0.20312	0.96429	0.65152	0.17308	0.16667	0.16667	0.32692	1	0.98276	0.5	1	0.94828	1	0.96667	1	1	0.87931	1	1	1	0.92857	0.96667	1	0.94828	0.775	0.83333	0.96552
BM26B	T	0	0	0	0	0.01515	0.01667	0.04167	0	0.04348	0.89744	0.61765	0.64062	0.35	1	0	0.03448	0	0	0.68571	0.06667	0.05172	0.06897	0.22222	0.85185	0.06667	0.08333	0.79688	0.03571	0.34848	0.82692	0.83333	0.83333	0.67308	0	0.01724	0.5	0	0.05172	0	0.03333	0	0	0.12069	0	0	0	0.07143	0.03333	0	0.05172	0.225	0.16667	0.03448
BM2G	G	1	1	1	1	1	1	1	1	1	0.17949	0.35294	0.36364	0.65	0	1	1	1	1	0.52941	1	0.98276	1	0.76316	0.16667	0.95	0.96667	0.21667	1	0.72727	0.71429	0.75862	0.7931	0.6	1	1	0.51724	1	1	1	1	1	1	1	1	1	1	1	1	1	1	1	1	1
BM2G	T	0	0	0	0	0	0	0	0	0	0.82051	0.64706	0.63636	0.35	1	0	0	0	0	0.47059	0	0.01724	0	0.23684	0.83333	0.05	0.03333	0.78333	0	0.27273	0.28571	0.24138	0.2069	0.4	0	0	0.48276	0	0	0	0	0	0	0	0	0	0	0	0	0	0	0	0	0
BM30A	A	1	1	1	1	1	1	1	1	1	0.225	0.41176	0.45312	0.66667	0.19231	1	1	1	1	0.76389	0.95	0.94828	1	0.75	0.22222	0.96667	0.95	0.40625	1	0.74242	0.38333	0.43333	0.5	0.51786	1	1	0.56452	1	1	1	1	1	1	0.96429	1	1	1	1	1	1	1	1	1	1
BM30A	G	0	0	0	0	0	0	0	0	0	0.775	0.58824	0.54688	0.33333	0.80769	0	0	0	0	0.23611	0.05	0.05172	0	0.25	0.77778	0.03333	0.05	0.59375	0	0.25758	0.61667	0.56667	0.5	0.48214	0	0	0.43548	0	0	0	0	0	0	0.03571	0	0	0	0	0	0	0	0	0	0
BM30C	A	0.01613	0	0.01613	0	0	0	0.02083	0.01667	0	0.46154	0.32353	0.36364	0.11667	0.59259	0	0	0.01724	0	0.56944	0.06667	0.03448	0.03448	0.13889	0.57692	0.03448	0.03333	0.56897	0	0.17188	0.53333	0.5	0.51667	0.51786	0	0	0.21667	0	0	0	0	0	0	0	0	0	0.01786	0	0	0	0	0	0	0
BM30C	T	0.98387	1	0.98387	1	1	1	0.97917	0.98333	1	0.53846	0.67647	0.63636	0.88333	0.40741	1	1	0.98276	1	0.43056	0.93333	0.96552	0.96552	0.86111	0.42308	0.96552	0.96667	0.43103	1	0.82812	0.46667	0.5	0.48333	0.48214	1	1	0.78333	1	1	1	1	1	1	1	1	1	0.98214	1	1	1	1	1	1	1
BM32A	A	0.28125	0.33333	0.15	0.17241	0.26562	0.23333	0.19048	0.23333	0.34783	0.14103	0.28125	0.07812	0.17241	0.02273	0.30435	0.22414	0.3	0.5	0.36765	0.36667	0.37931	0.36207	0.25	0.14583	0.48333	0.25862	0.16071	0.46429	0.35938	0.51852	0.43333	0.43333	0.48148	0.37037	0.41379	0.34483	0.39655	0.44444	0.40741	0.48214	0.35	0.53571	0.39286	0.38235	0.25	0.5	0.27586	0.51667	0.43333	0.375	0.5	0.53448	0.41667
BM32A	G	0.71875	0.66667	0.85	0.82759	0.73438	0.76667	0.80952	0.76667	0.65217	0.85897	0.71875	0.92188	0.82759	0.97727	0.69565	0.77586	0.7	0.5	0.63235	0.63333	0.62069	0.63793	0.75	0.85417	0.51667	0.74138	0.83929	0.53571	0.64062	0.48148	0.56667	0.56667	0.51852	0.62963	0.58621	0.65517	0.60345	0.55556	0.59259	0.51786	0.65	0.46429	0.60714	0.61765	0.75	0.5	0.72414	0.48333	0.56667	0.625	0.5	0.46552	0.58333
BM33B	A	1	1	1	1	1	1	0.97917	1	1	0.52941	0.58824	0.59375	0.7963	0.36538	1	1	1	1	0.85938	1	1	1	0.91667	0.38095	0.96667	1	0.5625	1	0.85484	0.7069	0.92857	0.71053	0.71429	1	1	0.68519	1	1	1	1	1	1	0.98276	1	1	1	1	1	1	1	1	1	1
BM33B	T	0	0	0	0	0	0	0.02083	0	0	0.47059	0.41176	0.40625	0.2037	0.63462	0	0	0	0	0.14062	0	0	0	0.08333	0.61905	0.03333	0	0.4375	0	0.14516	0.2931	0.07143	0.28947	0.28571	0	0	0.31481	0	0	0	0	0	0	0.01724	0	0	0	0	0	0	0	0	0	0
BM35C	A	0.42188	0.48333	0.3871	0.4	0.5	0.48276	0.30435	0.48333	0.3913	0.8375	0.67647	0.85484	0.76667	1	0.58	0.48276	0.60345	0.75	0.73611	0.53333	0.73214	0.46552	0.73684	0.84	0.65	0.6	0.9375	0.61111	0.63636	0.7	0.67241	0.62069	0.67308	0.56061	0.51724	0.80645	0.55357	0.53571	0.7	0.61111	0.5	0.55769	0.57143	0.66667	0.5	0.55	0.3	0.25	0.3	0.38889	0.275	0.22414	0.11667
BM35C	T	0.57812	0.51667	0.6129	0.6	0.5	0.51724	0.69565	0.51667	0.6087	0.1625	0.32353	0.14516	0.23333	0	0.42	0.51724	0.39655	0.25	0.26389	0.46667	0.26786	0.53448	0.26316	0.16	0.35	0.4	0.0625	0.38889	0.36364	0.3	0.32759	0.37931	0.32692	0.43939	0.48276	0.19355	0.44643	0.46429	0.3	0.38889	0.5	0.44231	0.42857	0.33333	0.5	0.45	0.7	0.75	0.7	0.61111	0.725	0.77586	0.88333
BM35D	A	1	1	1	0.98333	1	1	1	1	1	0.41892	0.67647	0.56667	0.81034	0.29167	1	1	1	1	0.875	1	0.96154	0.98276	0.8125	0.56522	0.98148	0.96429	0.40323	1	0.74138	0.76	0.73214	0.68519	0.83333	1	0.98148	0.53571	1	1	1	1	0.81818	1	1	1	1	1	0.91071	0.96667	0.96552	0.96296	0.825	0.78571	0.75
BM35D	G	0	0	0	0.01667	0	0	0	0	0	0.58108	0.32353	0.43333	0.18966	0.70833	0	0	0	0	0.125	0	0.03846	0.01724	0.1875	0.43478	0.01852	0.03571	0.59677	0	0.25862	0.24	0.26786	0.31481	0.16667	0	0.01852	0.46429	0	0	0	0	0.18182	0	0	0	0	0	0.08929	0.03333	0.03448	0.03704	0.175	0.21429	0.25
BM36F	A	0.90625	0.94828	0.91667	0.93333	0.96774	0.94643	0.97727	0.96552	0.93478	0.14103	0.35294	0.33333	0.65	0	0.91667	0.92	0.92857	0.875	0.41176	0.86667	0.89286	0.86207	0.63889	0.12963	0.88333	0.89286	0.18333	0.96154	0.71212	0.7069	0.46667	0.51852	0.67308	0.82	0.94828	0.41379	0.92857	0.94444	0.92857	0.93103	0.90909	0.92	0.98214	0.88235	1	0.90476	0.87037	0.95	0.92308	0.96552	0.925	0.9	0.89286
BM36F	C	0.09375	0.05172	0.08333	0.06667	0.03226	0.05357	0.02273	0.03448	0.06522	0.85897	0.64706	0.66667	0.35	1	0.08333	0.08	0.07143	0.125	0.58824	0.13333	0.10714	0.13793	0.36111	0.87037	0.11667	0.10714	0.81667	0.03846	0.28788	0.2931	0.53333	0.48148	0.32692	0.18	0.05172	0.58621	0.07143	0.05556	0.07143	0.06897	0.09091	0.08	0.01786	0.11765	0	0.09524	0.12963	0.05	0.07692	0.03448	0.075	0.1	0.10714
BM38B	A	0	0.03333	0	0.01667	0.01515	0.03333	0	0.01613	0.02174	0.65	0.5	0.51515	0.21667	0.96296	0	0	0.01667	0	0.36111	0.06667	0.15517	0.03448	0.23684	0.72222	0.08333	0.03333	0.6875	0.07143	0.25758	0.48276	0.51667	0.55	0.32143	0.12121	0.15517	0.56452	0.10345	0.05172	0.06667	0.21667	0.09091	0.07143	0.08621	0.05882	0	0.03333	0.15517	0.16667	0.15	0.15517	0.175	0.11667	0.13333
BM38B	G	1	0.96667	1	0.98333	0.98485	0.96667	1	0.98387	0.97826	0.35	0.5	0.48485	0.78333	0.03704	1	1	0.98333	1	0.63889	0.93333	0.84483	0.96552	0.76316	0.27778	0.91667	0.96667	0.3125	0.92857	0.74242	0.51724	0.48333	0.45	0.67857	0.87879	0.84483	0.43548	0.89655	0.94828	0.93333	0.78333	0.90909	0.92857	0.91379	0.94118	1	0.96667	0.84483	0.83333	0.85	0.84483	0.825	0.88333	0.86667
BM44B	A	0	0	0	0	0	0	0.02083	0	0	0.48718	0.26667	0.2931	0.13793	0.56	0	0	0	0	0.48611	0.1	0.05357	0	0.05556	0.38462	0.01724	0.03333	0.35714	0.01786	0.125	0.68333	0.60345	0.72414	0.64815	0	0	0.21667	0.01724	0	0	0	0	0	0.10345	0.02941	0	0	0	0	0	0	0	0	0
BM44B	G	1	1	1	1	1	1	0.97917	1	1	0.51282	0.73333	0.7069	0.86207	0.44	1	1	1	1	0.51389	0.9	0.94643	1	0.94444	0.61538	0.98276	0.96667	0.64286	0.98214	0.875	0.31667	0.39655	0.27586	0.35185	1	1	0.78333	0.98276	1	1	1	1	1	0.89655	0.97059	1	1	1	1	1	1	1	1	1
BM50B	A	0	0	0	0	0	0	0	0	0	0.0641	0.14706	0.07576	0.01667	0.11111	0	0	0	0	0.02778	0	0	0	0.02632	0.05769	0	0.01724	0.08065	0	0.0303	0.13793	0.11667	0.11667	0.05357	0	0	0.05172	0	0	0	0	0	0	0.01724	0	0	0	0	0	0	0	0	0	0
BM50B	G	1	1	1	1	1	1	1	1	1	0.9359	0.85294	0.92424	0.98333	0.88889	1	1	1	1	0.97222	1	1	1	0.97368	0.94231	1	0.98276	0.91935	1	0.9697	0.86207	0.88333	0.88333	0.94643	1	1	0.94828	1	1	1	1	1	1	0.98276	1	1	1	1	1	1	1	1	1	1
BM54A	A	1	1	1	1	1	1	0.97619	0.95455	1	0.08108	0.34375	0.35938	0.64286	0	1	1	1	1	0.25758	0.96667	0.94828	0.96552	0.76316	0.19231	0.96154	0.96667	0.23333	1	0.72727	0.01724	0.1	0.10714	0.2381	1	1	0.48276	0.98214	0.98276	1	1	1	1	0.98214	1	1	1	1	1	1	1	0.975	0.98333	0.98333
BM54A	G	0	0	0	0	0	0	0.02381	0.04545	0	0.91892	0.65625	0.64062	0.35714	1	0	0	0	0	0.74242	0.03333	0.05172	0.03448	0.23684	0.80769	0.03846	0.03333	0.76667	0	0.27273	0.98276	0.9	0.89286	0.7619	0	0	0.51724	0.01786	0.01724	0	0	0	0	0.01786	0	0	0	0	0	0	0	0.025	0.01667	0.01667
BM57A	C	1	1	1	1	1	1	1	1	1	1	1	1	1	1	1	1	1	1	1	0.98333	1	1	1	1	0.98333	1	1	1	1	1	1	1	1	0.87931	0.87931	1	0.98276	0.73214	0.82759	0.7037	1	1	1	1	1	0.96667	0.46552	0.27586	0.36667	0.41379	0.23684	0.3	0.3
BM57A	T	0	0	0	0	0	0	0	0	0	0	0	0	0	0	0	0	0	0	0	0.01667	0	0	0	0	0.01667	0	0	0	0	0	0	0	0	0.12069	0.12069	0	0.01724	0.26786	0.17241	0.2963	0	0	0	0	0	0.03333	0.53448	0.72414	0.63333	0.58621	0.76316	0.7	0.7
BM57D	A	0	0	0	0	0	0	0	0	0	0	0	0	0	0	0	0	0	0	0.01389	0.02083	0	0	0.02632	0	0.01923	0	0	0	0	0	0	0	0	0.16667	0.13462	0	0.01724	0.24138	0.18333	0.32143	0	0	0	0	0	0.01923	0.45	0.61667	0.51724	0.5	0.725	0.7	0.61667
BM57D	C	1	1	1	1	1	1	1	1	1	1	1	1	1	1	1	1	1	1	0.98611	0.97917	1	1	0.97368	1	0.98077	1	1	1	1	1	1	1	1	0.83333	0.86538	1	0.98276	0.75862	0.81667	0.67857	1	1	1	1	1	0.98077	0.55	0.38333	0.48276	0.5	0.275	0.3	0.38333
BM5B	A	1	1	1	1	1	1	1	1	0.95652	0.925	0.97059	0.9697	0.98333	0.98077	1	1	1	1	1	1	0.98276	1	1	0.94444	1	0.98333	0.96552	1	1	0.98333	0.96429	1	0.96429	1	1	0.98333	1	0.98276	1	1	1	1	1	1	1	0.98333	1	0.96667	1	1	0.975	0.9	0.85
BM5B	G	0	0	0	0	0	0	0	0	0.04348	0.075	0.02941	0.0303	0.01667	0.01923	0	0	0	0	0	0	0.01724	0	0	0.05556	0	0.01667	0.03448	0	0	0.01667	0.03571	0	0.03571	0	0	0.01667	0	0.01724	0	0	0	0	0	0	0	0.01667	0	0.03333	0	0	0.025	0.1	0.15
BM5D	C	0.65625	0.8	0.77419	0.68333	0.68182	0.71667	0.80435	0.70968	0.6087	0.775	0.79412	0.83333	0.78333	0.85185	0.82	0.7069	0.61667	0.875	0.68056	0.65	0.56897	0.56897	0.65789	0.77778	0.66667	0.66667	0.8125	0.5	0.69697	0.36667	0.4	0.38333	0.44643	0.57812	0.58621	0.54839	0.5	0.7963	0.67241	0.77586	0.63636	0.42857	0.51786	0.47059	0.75	0.48333	0.77586	0.81667	0.73333	0.81034	0.95	0.85	0.85
BM5D	T	0.34375	0.2	0.22581	0.31667	0.31818	0.28333	0.19565	0.29032	0.3913	0.225	0.20588	0.16667	0.21667	0.14815	0.18	0.2931	0.38333	0.125	0.31944	0.35	0.43103	0.43103	0.34211	0.22222	0.33333	0.33333	0.1875	0.5	0.30303	0.63333	0.6	0.61667	0.55357	0.42188	0.41379	0.45161	0.5	0.2037	0.32759	0.22414	0.36364	0.57143	0.48214	0.52941	0.25	0.51667	0.22414	0.18333	0.26667	0.18966	0.05	0.15	0.15
BM60A	A	0	0	0	0	0	0.01667	0	0	0.05	0.24359	0.11765	0.21212	0.1	0.22	0.02083	0	0	0	0.26389	0.05	0.01724	0	0.13889	0.24074	0.01667	0.03333	0.1875	0	0.06061	0.3	0.58333	0.46667	0.625	0	0	0.14516	0	0	0.03333	0	0	0	0.06897	0.05882	0	0.01786	0	0	0.01667	0	0	0	0
BM60A	G	1	1	1	1	1	0.98333	1	1	0.95	0.75641	0.88235	0.78788	0.9	0.78	0.97917	1	1	1	0.73611	0.95	0.98276	1	0.86111	0.75926	0.98333	0.96667	0.8125	1	0.93939	0.7	0.41667	0.53333	0.375	1	1	0.85484	1	1	0.96667	1	1	1	0.93103	0.94118	1	0.98214	1	1	0.98333	1	1	1	1
BM61A	C	0.48333	0.4	0.56452	0.31667	0.4697	0.5	0.6875	0.67857	0.5	0.3125	0.41176	0.27273	0.33333	0	0.56	0.74138	0.7931	0.375	0.51471	0.78571	0.67857	0.82759	0.58333	0.27778	0.56897	0.75	0.23333	0.75	0.54688	0.58621	0.58929	0.66667	0.58929	0.69643	0.81034	0.55172	0.77586	0.74138	0.71154	0.76667	0.86364	0.68	0.7069	0.82353	0.75	0.82143	0.75926	0.75862	0.78571	0.80357	0.925	0.84483	0.89655
BM61A	T	0.51667	0.6	0.43548	0.68333	0.5303	0.5	0.3125	0.32143	0.5	0.6875	0.58824	0.72727	0.66667	1	0.44	0.25862	0.2069	0.625	0.48529	0.21429	0.32143	0.17241	0.41667	0.72222	0.43103	0.25	0.76667	0.25	0.45312	0.41379	0.41071	0.33333	0.41071	0.30357	0.18966	0.44828	0.22414	0.25862	0.28846	0.23333	0.13636	0.32	0.2931	0.17647	0.25	0.17857	0.24074	0.24138	0.21429	0.19643	0.075	0.15517	0.10345
BM62A	A	0.04688	0.05	0.04839	0.06667	0.0303	0.13333	0.32609	0.36207	0.23913	0.95	0.71875	0.83333	0.68333	1	0.58	0.17857	0.22414	0	0.69444	0.08333	0.18966	0.06897	0.42105	0.87037	0.15517	0.05	0.78125	0.03704	0.34375	0.88333	0.95	0.94828	0.80357	0	0	0.54839	0	0	0	0	0	0	0.06897	0	0	0	0	0	0	0	0	0	0
BM62A	G	0.95312	0.95	0.95161	0.93333	0.9697	0.86667	0.67391	0.63793	0.76087	0.05	0.28125	0.16667	0.31667	0	0.42	0.82143	0.77586	1	0.30556	0.91667	0.81034	0.93103	0.57895	0.12963	0.84483	0.95	0.21875	0.96296	0.65625	0.11667	0.05	0.05172	0.19643	1	1	0.45161	1	1	1	1	1	1	0.93103	1	1	1	1	1	1	1	1	1	1
BM64A	C	1	1	1	1	1	1	1	1	1	0.1375	0.35294	0.36364	0.65	0	1	1	1	1	0.41667	0.89655	0.89655	1	0.76316	0.12963	0.95	0.96667	0.23438	1	0.74242	0.43333	0.33333	0.45	0.53846	1	0.98276	0.46774	1	1	1	1	1	1	0.94828	1	1	1	1	1	0.98333	1	1	1	1
BM64A	T	0	0	0	0	0	0	0	0	0	0.8625	0.64706	0.63636	0.35	1	0	0	0	0	0.58333	0.10345	0.10345	0	0.23684	0.87037	0.05	0.03333	0.76562	0	0.25758	0.56667	0.66667	0.55	0.46154	0	0.01724	0.53226	0	0	0	0	0	0	0.05172	0	0	0	0	0	0.01667	0	0	0	0
BM67C	A	0.91935	0.74138	0.83871	0.81667	0.89062	0.89655	0.93478	0.75806	0.86364	0.1125	0.41176	0.22581	0.53448	0	0.86	0.81034	0.96667	0.625	0.41667	0.83333	0.7963	0.89286	0.63158	0.14815	0.72414	0.86667	0.17742	0.78571	0.62121	0.10714	0.01667	0.125	0.14286	0.8871	0.88889	0.43548	0.81034	0.74138	0.66667	0.86667	0.81818	0.68519	0.80357	0.875	0.625	0.80357	0.48276	0.5	0.58621	0.60345	0.10526	0.18966	0.1
BM67C	T	0.08065	0.25862	0.16129	0.18333	0.10938	0.10345	0.06522	0.24194	0.13636	0.8875	0.58824	0.77419	0.46552	1	0.14	0.18966	0.03333	0.375	0.58333	0.16667	0.2037	0.10714	0.36842	0.85185	0.27586	0.13333	0.82258	0.21429	0.37879	0.89286	0.98333	0.875	0.85714	0.1129	0.11111	0.56452	0.18966	0.25862	0.33333	0.13333	0.18182	0.31481	0.19643	0.125	0.375	0.19643	0.51724	0.5	0.41379	0.39655	0.89474	0.81034	0.9
BM6C	C	0.34375	0.36667	0.29032	0.33333	0.28788	0.28333	0.475	0.36	0.35	0.3875	0.40625	0.28788	0.41667	0.36538	0.375	0.37931	0.33333	0	0.36111	0.15	0.36207	0.30357	0.42105	0.37037	0.34483	0.31667	0.40625	0.23214	0.40909	0.58333	0.68333	0.6	0.46429	0.01515	0.08621	0.32258	0.32	0.07407	0.06897	0.07407	0.09091	0.08929	0.21429	0	0	0.08333	0	0.01667	0	0	0	0	0
BM6C	T	0.65625	0.63333	0.70968	0.66667	0.71212	0.71667	0.525	0.64	0.65	0.6125	0.59375	0.71212	0.58333	0.63462	0.625	0.62069	0.66667	1	0.63889	0.85	0.63793	0.69643	0.57895	0.62963	0.65517	0.68333	0.59375	0.76786	0.59091	0.41667	0.31667	0.4	0.53571	0.98485	0.91379	0.67742	0.68	0.92593	0.93103	0.92593	0.90909	0.91071	0.78571	1	1	0.91667	1	0.98333	1	1	1	1	1
BM75C	C	1	1	1	1	1	1	1	1	1	0.93056	1	1	1	0.98	1	1	1	1	0.85294	0.98333	0.94643	1	1	0.85185	1	0.96667	0.93333	1	0.98276	0.82759	0.75	0.71429	0.77778	1	1	0.94828	1	1	1	1	1	1	1	1	1	1	1	1	1	1	1	1	1
BM75C	G	0	0	0	0	0	0	0	0	0	0.06944	0	0	0	0.02	0	0	0	0	0.14706	0.01667	0.05357	0	0	0.14815	0	0.03333	0.06667	0	0.01724	0.17241	0.25	0.28571	0.22222	0	0	0.05172	0	0	0	0	0	0	0	0	0	0	0	0	0	0	0	0	0
BM78B	A	0.04839	0	0	0.06667	0.03226	0.05357	0	0	0.02632	0.02564	0	0.01613	0	0	0	0.03448	0.01786	0.25	0.08824	0.17857	0.14286	0.05172	0.02632	0.04	0.15517	0.05172	0.01613	0.01786	0.03125	0.01667	0.01667	0.03448	0.05357	0.27419	0.19643	0.1	0.08929	0.28571	0.03333	0.19643	0.1	0.16071	0.25926	0.17647	0.25	0.32759	0.63333	0.5	0.68966	0.51786	0.8	0.68333	0.81667
BM78B	G	0.95161	1	1	0.93333	0.96774	0.94643	1	1	0.97368	0.97436	1	0.98387	1	1	1	0.96552	0.98214	0.75	0.91176	0.82143	0.85714	0.94828	0.97368	0.96	0.84483	0.94828	0.98387	0.98214	0.96875	0.98333	0.98333	0.96552	0.94643	0.72581	0.80357	0.9	0.91071	0.71429	0.96667	0.80357	0.9	0.83929	0.74074	0.82353	0.75	0.67241	0.36667	0.5	0.31034	0.48214	0.2	0.31667	0.18333
BM8E	A	0.01562	0.01667	0.04839	0	0.01515	0	0.02083	0.01613	0.04348	0.8875	0.61765	0.65152	0.38333	1	0.04	0	0.05	0	0.63889	0.08333	0.14286	0.05172	0.23684	0.87037	0.05	0.06667	0.75806	0.01852	0.28788	0.46667	0.56667	0.66667	0.5	0	0	0.58333	0.01724	0.01786	0	0	0	0.01786	0	0.02941	0	0	0.01667	0	0	0	0.025	0.06897	0
BM8E	G	0.98438	0.98333	0.95161	1	0.98485	1	0.97917	0.98387	0.95652	0.1125	0.38235	0.34848	0.61667	0	0.96	1	0.95	1	0.36111	0.91667	0.85714	0.94828	0.76316	0.12963	0.95	0.93333	0.24194	0.98148	0.71212	0.53333	0.43333	0.33333	0.5	1	1	0.41667	0.98276	0.98214	1	1	1	0.98214	1	0.97059	1	1	0.98333	1	1	1	0.975	0.93103	1
BM92B	A	1	1	1	1	1	1	1	1	1	0.11538	0.38235	0.35938	0.68333	0	1	1	1	1	0.34722	0.91667	0.93103	0.98276	0.76316	0.11538	0.96552	0.96667	0.20968	1	0.72727	0.18966	0.15	0.16667	0.32143	1	1	0.45161	1	1	1	1	1	1	1	1	1	1	1	1	1	1	1	1	1
BM92B	T	0	0	0	0	0	0	0	0	0	0.88462	0.61765	0.64062	0.31667	1	0	0	0	0	0.65278	0.08333	0.06897	0.01724	0.23684	0.88462	0.03448	0.03333	0.79032	0	0.27273	0.81034	0.85	0.83333	0.67857	0	0	0.54839	0	0	0	0	0	0	0	0	0	0	0	0	0	0	0	0	0
BM9B	A	0.46667	0.64815	0.6	0.53448	0.59375	0.5	0.58696	0.55	0.47619	0.37931	0.5	0.32	0.54	0.45	0.35417	0.5	0.51724	0.25	0.32609	0.61538	0.56	0.69231	0.66667	0.44737	0.62963	0.36667	0.47917	0.57407	0.5	0.475	0.61905	0.60526	0.55769	0.58621	0.41071	0.63043	0.44828	0.33929	0.46667	0.31034	0.54545	0.56522	0.63793	0.67647	0.5	0.62069	0.24138	0.33333	0.19643	0.32759	0.125	0.08333	0.2
BM9B	G	0.53333	0.35185	0.4	0.46552	0.40625	0.5	0.41304	0.45	0.52381	0.62069	0.5	0.68	0.46	0.55	0.64583	0.5	0.48276	0.75	0.67391	0.38462	0.44	0.30769	0.33333	0.55263	0.37037	0.63333	0.52083	0.42593	0.5	0.525	0.38095	0.39474	0.44231	0.41379	0.58929	0.36957	0.55172	0.66071	0.53333	0.68966	0.45455	0.43478	0.36207	0.32353	0.5	0.37931	0.75862	0.66667	0.80357	0.67241	0.875	0.91667	0.8
BM9C	A	0.36667	0.24138	0.28333	0.31034	0.25758	0.39286	0.21739	0.26667	0.27273	0.07692	0.08824	0.16667	0.25862	0.05556	0.32609	0.42593	0.39286	0.625	0.10294	0.21667	0.2069	0.25	0.22222	0.11538	0.3	0.55172	0.12069	0.33929	0.21212	0.08333	0.13333	0.18333	0.23214	0.37097	0.53448	0.16129	0.44828	0.58621	0.53333	0.67241	0.36364	0.2963	0.27586	0.20588	0.25	0.28333	0.71667	0.65	0.73333	0.67241	0.825	0.86667	0.77586
BM9C	C	0.11667	0.12069	0.16667	0.13793	0.12121	0.10714	0.17391	0.16667	0.22727	0.01282	0.11765	0.07576	0.12069	0	0.30435	0.09259	0.16071	0	0	0.05	0.12069	0.01786	0.08333	0.01923	0.03333	0.03448	0.01724	0.08929	0.09091	0	0.01667	0.03333	0.05357	0.03226	0.03448	0.01613	0.08621	0.05172	0	0.01724	0.09091	0.07407	0.03448	0.05882	0.25	0.1	0.03333	0.01667	0.05	0	0.025	0.05	0.08621
BM9C	T	0.51667	0.63793	0.55	0.55172	0.62121	0.5	0.6087	0.56667	0.5	0.91026	0.79412	0.75758	0.62069	0.94444	0.36957	0.48148	0.44643	0.375	0.89706	0.73333	0.67241	0.73214	0.69444	0.86538	0.66667	0.41379	0.86207	0.57143	0.69697	0.91667	0.85	0.78333	0.71429	0.59677	0.43103	0.82258	0.46552	0.36207	0.46667	0.31034	0.54545	0.62963	0.68966	0.73529	0.5	0.61667	0.25	0.33333	0.21667	0.32759	0.15	0.08333	0.13793

Supplementary Table S3. The environmental variables data for 53 Mytilus mussel samples,

Name	Coordinates		Sample collection	Chlorophyll-a concentration	Sea ice concentration	Wind speed	Cloud amount	Concentration of cilicates	Concentration of nitrates	Concentration of phosphates	Salinity	Water temperature	Tide height	Swell height	Solar radiation	Precipitation
BNJ	40°11'13.56"N	74° 0'36.36"W	2012	3.110	0	6.34	4.323	0.712	1.126	0.363	31.506	13.252	60.9782	1.435	13780.583	97.417
IRD	36°52'6.19"N	75°58'2.16"W	2012	4.321	0	6.75	4.209	1.257	1.04	0.265	30.585	15.868	49.4345	1.646	14748.25	95.5
KPV	37° 9'51.12"N	75°59'29.40"W	2012	3.837	0	6.75	4.209	0.805	1.019	0.321	28.814	15.322	40.1819	1.646	14792.917	90
MID	38°56'42.00"N	75°18'38.88"W	2012	10.919	0	6.31	4.041	0.611	0.941	0.341	30.799	16.465	65.3946	1.508	14253.5	93.167
PNY	40°35'34.80"N	73°34'30.72"W	2012	2.486	0.708	5.38	4.407	0.712	1.126	0.363	31.374	13.215	62.1477	1.435	13749.833	87.75
SNY	40°55'15.96"N	73° 9'0.36"W	2012	4.538	2.017	5.37	4.407	2.094	2.241	0.416	30.767	11.835	118.651	1.435	13684.583	91.75
NWF	49°30'5.32"N	55°41'44.21"W	2012	1.038	26.3	7.11	5.370	5.274	4.265	0.598	31.512	4.601	34.9059	2.001	10933.333	86.333
PEI	46°26'11.10"N	62°40'24.06"W	2012	6.834	21.525	6.97	5.262	3.848	1.357	0.541	29.284	8.667	15.501	1.775	12129.333	100.833
SNS	44°48'5.13"N	62°50'13.55"W	2012	0.890	6.267	6.85	4.998	2.707	0.894	0.506	31.013	9.248	60.3177	1.837	12358.917	125
KKA	44°30'33.79"N	63°29'24.91"W	1996	1.233	7.883	6.85	4.998	2.447	1.247	0.488	30.839	8.815	61.2347	1.837	12475.5	118.417
PBAY	47° 2'40.05"N	54°11'34.72"W	2012	0.796	7.642	8.53	5.653	1.085	0.715	0.385	31.695	5.924	66.5668	2.134	11189.75	122.25
GLD	71° 8'42.96"N	51°16'31.99"W	2012	1.019	66.758	7.67	5.102	4.846	2.093	0.368	33.05	2.915	47.2764	1.816	8069.4167	18.083
GLL	70°59'42.42"N	52°16'41.37"W	2012	1.213	62.808	7.32	5.102	4.846	2.093	0.368	32.998	2.436	46.9213	1.816	8580	14.667
SAV	76° 1'5.26"N	65°7'4.18"W	2015	0.597	70.092	6.6	4.593	3.821	2.206	0.568	32.461	-0.149	62.0604	1.555	8387.75	12.917
NUU	64°10'24.36"N	51°29'25.86"W	2015	1.105	7.025	7.11	5.879	2.01	3.917	0.587	32.634	1.175	131.633	2.06	8911.3333	58.417
ISLB	64° 8'59.03"N	21°53'16.61"W	1986	1.673	0.264	8.41	6.299	5.499	9.273	0.682	34.714	7.891	129.027	2.59	7761	78.167
ISLR	64° 5'44.18"N	21°56'48.83"W	2004	1.834	0.264	8.41	6.299	5.499	9.273	0.682	34.714	7.891	128.738	2.59	7774.0833	79
SPI	79°38'46.94"N	11°14'10.38"E	2014	0.569	10.7	6.57	5.827	3.429	4.654	0.399	34.653	4.766	41.0366	1.793	6650.25	35
BRN	60°23'25.65"N	5°12'27.08"E	2012	0.029	1.508	7.29	5.717	2.408	3.8	0.332	32.846	9.883	46.8821	1.903	9029.8333	168.5
BODS	67°17'0.61"N	14°21'48.02"E	2013	1.358	2.092	6.74	5.716	2.5	4.025	0.371	32.543	8.098	84.7105	1.737	8165.8333	89.167
BODZ	67°17'45.25"N	14°23'49.53"E	2013	1.474	2.092	6.74	5.716	2.5	4.025	0.371	32.543	8.098	84.7105	1.737	8168.1667	87.667
TRO	69°35'27.68"N	18°53'20.62"E	2006	1.131	16.675	7.28	5.621	2.741	4.638	0.403	34.051	7.28	82.9871	1.644	7712.9167	80.167
BAR	69°47'36.00"N	32°45'59.68"E	2004	0.999	2.925	8.21	6.187	4.492	2.693	0.424	34.016	6.432	110.671	1.483	7598.9167	43.833
KOL	68°58'56.47"N	33° 1'36.08"E	2014	3.316	4.033	7.33	6.187	2.049	1.646	0.291	33.537	6.126	115.045	1.332	7733.9167	38.75
DLZ	69° 5'16.56"N	36° 3'3.42"E	2014	1.323	4.033	7.49	6.261	2.049	1.646	0.291	33.669	5.47	126.026	1.332	7664.0833	40.333
WSBS	66°33'5.62"N	33° 6'50.58"E	2014	3.149	57.108	4.57	5.953	7.697	2.487	0.142	23.421	4.754	71.8569	1.287	8193.75	42.667
CHU	66°16'12.31"N	33° 4'12.93"E	2014	4.040	54.108	4.11	5.953	7.697	2.487	0.142	23.534	4.609	65.1236	1.287	8156.6667	43.5
ONE	66°15'51.67"N	33° 2'54.21"E	1997	4.040	54.108	4.11	5.953	7.697	2.487	0.142	23.534	4.609	65.1236	1.287	8156.6667	43.5
KER	66°17'22.66"N	33°40'6.28"E	2014	2.368	53.425	4.13	5.953	7.697	2.487	0.142	23.534	4.609	64.0687	1.287	8216.75	41.667
BIA	54°49'55.99"N	17°57'9.02"E	2014	1.292	1.725	7.08	4.992	10.853	1.257	0.311	7.498	9.87	1.25491	1.201	10262.083	51.083
BOR	55° 4'25.89"N	14°43'56.56"E	2013	1.841	2.85	4.66	4.973	12.38	2.127	0.368	7.703	9.744	0.5041	1.152	10180.25	46.833
KLA	55°49,4'N	20°30,2E	2013	2.440	3.175	5.96	5.173	10.666	2.118	0.311	6.7	10.369	0.79823	1.24	10327	61.75
STC	55°16'50.25"N	12°26'49.85"E	2014	2.519	5.167	4.8	5.080	5.633	1.709	0.274	16.018	9.881	0.84969	1.135	9910.6667	47.917
CIS	60°40'0.37"N	0°56'40.85"W	2012	0.624	0.125	9.21	6.335	2.516	5.654	0.475	35.268	10.479	68.8636	2.09	7901.5	102.167
SCO	57° 4'24.00"N	5°47'24.00"W	2014	1.435	0	7.95	3.152	2.363	3.927	0.417	34.403	10.699	140.388	2.182	8392.0833	175.167
LET	56°27'21.35"N	5°18'26.62"W	2008	0.437	0	7.41	3.152	2.363	3.927	0.417	34.023	10.579	122.624	2.182	8459.3333	133.917
OBA	56°24'49.40"N	5°28'23.00"W	2014	0.855	0	7.41	3.152	2.363	3.927	0.417	34.023	10.579	121.022	2.182	8569.75	144.417
IONA	56°19'52.72"N	6°23'29.93"W	2014	0.866	0	7.9	4.781	2.363	3.927	0.417	34.338	10.725	122.023	2.182	8866.6667	132.917
KRR	56°22'42.56"N	5°33'17.14"W	2014	1.990	0	7.9	3.152	2.363	3.927	0.417	34.023	10.579	120.999	2.182	8606.8333	146.917
STA	56°26'9.98"N	6°20'15.43"W	2014	1.427	0	7.9	4.781	2.363	3.927	0.417	34.338	10.725	125.313	2.182	8672.8333	142.333
GBLO	52°20'44.07"N	1°45'27.63"E	2000	5.709	2.6	7.99	4.919	3.638	6.97	0.508	34.416	11.862	67.5736	1.505	10094.417	46.75
LGF	55° 5'35.50"N	7° 4'48.92"W	2006	27.548	0	8.53	5.609	2.269	4.326	0.397	34.407	10.924	64.4506	2.336	8924.3333	105.083
SAL	58°52'45.38"N	11° 7'13.18"E	2014	1.626	2.392	4.77	4.931	3.054	2.248	0.238	27.177	10.005	11.2114	1.354	9576.4167	62.083
NLOO	51°50'7.10"N	3°49'18.21"E	2000	6.361	1.7	7.07	5.278	4.435	9.446	0.569	32.592	12.393	95.4315	1.56	10190.583	62.417
MSM	48°39'0.06"N	1°31'40.26"W	2013	5.093	0	6.55	5.300	2.314	3.698	0.302	34.011	12.979	389.656	1.852	11483.167	59.25
LOI	47°14'43.83"N	2°13'48.88"W	2004	7.945	0	5.96	4.823	3.436	3.784	0.14	33.374	14.059	159.543	1.972	12423.583	66.833
BID	43°21'38.71"N	1°51'11.15"W	2004	1.191	0	5.41	4.809	2.845	1.926	0.1	35.043	16.359	131.985	1.998	12625.083	115
VIG	42°13'54.12"N	8°45'7.22"W	2004	1.577	0	6.72	4.593	1.203	1.223	0.098	35.589	15.52	108.124	2.486	14055.333	130.5
CAS	38°34'14.89"N	9°19'8.95"W	2013	1.369	0	5.51	3.753	1.021	1.609	0.18	35.867	16.765	98.2744	2.171	16501.5	55.25
CAM	36° 3'30.09"N	5°46'8.88"W	2004	0.512	0	3.23	3.087	2.169	1.286	0.167	36.399	18.465	61.5338	1.544	17636	70.417
IMC	39°47'59.88"N	8°31'9.72"E	2004	1.115	0	5.96	3.433	2.174	1.023	0.121	37.751	19.252	7.02792	1.559	15841.417	42
NEA	40°46'44.64"N	14° 5'28.20"E	2014	0.497	0	5.08	3.784	1.477	0.093	0.169	37.889	19.846	11.7012	1.148	15111.583	75.75
AZO	45°43'51.71"N	35° 5'0.26"E	1997	4.990	0	5.84	4.160	11.226	0.468	0.228	13.813	14.545	0.49364	1.173	12778.5	30.5

Supplementary Table S4. Population specific FIS indices per polymorphic locus (absolute values)

Locus		BNJ	IRD	KPV	MID	PNY	SNY	NWF	PEI	SNS	KKA	PBAY	GLD	GLL	SAV	NUU	ISLB	ISLR	SPI	BRN	BODS	BODZ	TRO	BAR	KOL	DLZ	WSBS	CHU	ONE	KER	BIA	BOR	KLA	STC	CIS	SCO	LET	OBA	IONA	KRR	STA	GBLO	LGF	SAL	NLOO	MSM	LOI	BID	VIG	CAS	CAM	IMC	NEA	AZO	Average FIS
BM101A	1	N.A.	N.A.	N.A.	N.A.	N.A.	N.A.	N.A.	N.A.	N.A.	N.A.	N.A.	N.A.	N.A.	N.A.	N.A.	N.A.	N.A.	0	N.A.	0	-0.04	-0.02	N.A.	1	N.A.	N.A.	N.A.	N.A.	N.A.	N.A.	N.A.	N.A.	N.A.	0.328	-0.16	N.A.	N.A.	0.176	0.265	0.143	N.A.	-0.02	N.A.	N.A.	N.A.	N.A.	-0.04	0.659	-0.02	N.A.	0	N.A.	N.A.	0.167
BM102A	2	-0.13	-0.19	0.011	0.149	-0	-0.3	-0.01	-0.04	-0.22	1	1	0.596	0.236	N.A.	0.208	0.103	0.051	-0.5	0.391	-0.05	0.305	-0.13	0.159	0.836	0.185	-0.05	0.634	-0.33	0.224	0.926	0.638	1	0.716	-0.08	-0.03	0.451	0.239	-0.06	0.345	-0.28	0.107	0.04	-0.06	0.063	-0.2	-0.31	-0.37	0.036	0.164	-0.18	0.159	-0.13	-0.14	0.102
BM103B	3	N.A.	N.A.	N.A.	N.A.	N.A.	N.A.	N.A.	N.A.	N.A.	-0.11	0.437	0.333	0.515	-0.02	N.A.	N.A.	N.A.	N.A.	0.335	0.275	-0.06	N.A.	0.621	0.169	0.477	0.527	0.028	0.35	0.28	-0.1	-0.21	0.237	-0.06	N.A.	N.A.	0.609	N.A.	N.A.	N.A.	N.A.	N.A.	N.A.	0.273	N.A.	N.A.	N.A.	N.A.	N.A.	N.A.	N.A.	N.A.	N.A.	N.A.	0.198
BM105A	4	N.A.	N.A.	N.A.	N.A.	N.A.	N.A.	N.A.	N.A.	N.A.	N.A.	N.A.	N.A.	N.A.	N.A.	N.A.	N.A.	N.A.	N.A.	N.A.	-0.02	-0.04	0	N.A.	N.A.	N.A.	N.A.	N.A.	N.A.	N.A.	N.A.	N.A.	N.A.	N.A.	-0.14	-0.04	0	0.477	0.291	-0.05	-0.02	0	0	N.A.	0	N.A.	-0.09	0.188	0.275	0.458	-0.04	0.518	0.477	0.477	0.211
BM106B	5	N.A.	N.A.	N.A.	N.A.	N.A.	N.A.	N.A.	N.A.	N.A.	0	N.A.	N.A.	N.A.	N.A.	N.A.	N.A.	N.A.	N.A.	N.A.	N.A.	N.A.	N.A.	N.A.	N.A.	N.A.	N.A.	N.A.	N.A.	N.A.	N.A.	N.A.	N.A.	N.A.	N.A.	N.A.	N.A.	N.A.	N.A.	N.A.	N.A.	N.A.	N.A.	N.A.	N.A.	N.A.	N.A.	N.A.	1	N.A.	N.A.	1	1	N.A.	0.955
BM10B	6	N.A.	N.A.	N.A.	N.A.	N.A.	N.A.	N.A.	N.A.	N.A.	-0.14	0.776	0.183	0.474	0.029	N.A.	N.A.	N.A.	N.A.	0.002	-0.04	0	0	0.862	0.066	0.659	1	0.5	N.A.	0.916	-0.05	-0.23	-0.43	0.143	N.A.	N.A.	0.404	N.A.	N.A.	N.A.	N.A.	N.A.	N.A.	N.A.	N.A.	N.A.	N.A.	N.A.	N.A.	N.A.	N.A.	N.A.	N.A.	N.A.	0.196
BM113A	7	N.A.	N.A.	N.A.	N.A.	N.A.	N.A.	N.A.	N.A.	N.A.	0.032	0.644	0.359	0.581	0	N.A.	N.A.	N.A.	0	0.139	0.477	-0.02	0	0.862	0.841	0.477	1	0.641	N.A.	0.923	-0.07	-0.09	-0.04	0	N.A.	N.A.	0.545	N.A.	N.A.	N.A.	N.A.	N.A.	N.A.	-0.02	N.A.	N.A.	N.A.	N.A.	N.A.	N.A.	N.A.	N.A.	N.A.	N.A.	0.421
BM118A	8	N.A.	N.A.	N.A.	N.A.	N.A.	N.A.	N.A.	N.A.	N.A.	-0.01	N.A.	N.A.	N.A.	N.A.	N.A.	0.477	N.A.	N.A.	0	0	N.A.	-0.02	0	N.A.	N.A.	0	N.A.	-0.02	N.A.	N.A.	N.A.	N.A.	N.A.	-0.02	-0.06	N.A.	N.A.	-0.02	N.A.	-0.04	N.A.	-0.04	N.A.	0	N.A.	0	-0.22	0.015	0.357	-0.08	0.147	0.133	-0.07	0.037
BM11A	9	-0.03	0	-0.02	N.A.	0	0	-0.07	-0.03	-0.02	0.177	0.508	0.412	0.657	N.A.	N.A.	N.A.	N.A.	N.A.	0.095	-0.02	-0.06	-0.02	0.862	0.612	0.659	1	0.537	0	1	-0.09	-0.16	-0.09	0.513	N.A.	N.A.	0.676	N.A.	N.A.	N.A.	N.A.	N.A.	N.A.	0.477	N.A.	N.A.	0	N.A.	N.A.	N.A.	N.A.	N.A.	N.A.	0	0.396
BM121A	10	N.A.	N.A.	N.A.	N.A.	N.A.	N.A.	N.A.	N.A.	N.A.	0	N.A.	N.A.	N.A.	N.A.	N.A.	N.A.	N.A.	N.A.	0.183	0	-0.06	N.A.	N.A.	-0.04	N.A.	N.A.	N.A.	N.A.	N.A.	0.079	0.298	0.183	-0.01	N.A.	N.A.	-0.02	N.A.	N.A.	N.A.	N.A.	N.A.	N.A.	-0.02	N.A.	N.A.	N.A.	N.A.	N.A.	N.A.	N.A.	N.A.	N.A.	N.A.	0.128
BM12A	11	-0.05	0.237	-0.07	-0.05	-0.07	-0.04	0	-0.02	0	-0.08	0.66	0.306	0.532	N.A.	N.A.	N.A.	0	0	0.233	-0.02	0.205	-0.02	0.862	0.612	0.477	0.659	0.716	0	1	0.437	0.102	-0.06	0.107	N.A.	N.A.	0.747	N.A.	N.A.	N.A.	N.A.	0	0	-0.1	0	N.A.	-0.02	N.A.	N.A.	0	0	N.A.	N.A.	N.A.	0.320
BM12C	12	0.006	0.169	-0.09	0.102	-0.1	-0.04	-0.05	-0.02	-0.02	0.422	0.355	0.265	0.508	-0.3	N.A.	-0.08	0.151	1	0.017	0.183	-0.03	-0.08	0.008	-0.06	0	-0.09	0.1	-0.02	0.907	0.314	0.449	0.481	-0.05	-0.14	-0.08	0.563	0.514	0.787	-0.02	-0.08	0	-0.08	-0.02	-0.2	N.A.	-0.19	-0.1	-0.26	-0.14	0.008	-0.07	-0.24	0.277	0.157
BM151A	13	N.A.	N.A.	N.A.	N.A.	N.A.	N.A.	N.A.	N.A.	N.A.	N.A.	N.A.	N.A.	N.A.	N.A.	N.A.	N.A.	N.A.	N.A.	N.A.	N.A.	N.A.	N.A.	N.A.	N.A.	N.A.	N.A.	N.A.	N.A.	N.A.	N.A.	N.A.	N.A.	N.A.	N.A.	0	N.A.	N.A.	N.A.	N.A.	N.A.	N.A.	0	N.A.	N.A.	N.A.	N.A.	N.A.	N.A.	N.A.	N.A.	N.A.	N.A.	N.A.	0.001
BM16B	14	0.135	0.057	0.155	0.439	0.016	0.208	-0.02	-0.11	0.341	-0.07	-0.03	0.216	0.102	N.A.	0.143	0.291	-0.26	N.A.	0.167	-0.19	-0.14	0.089	-0.06	-0.04	0.085	-0.23	N.A.	-0.13	0.153	0.521	-0.14	-0.09	0.379	0.099	0.051	-0.13	-0.15	0.008	-0.05	-0.17	-0.18	-0.1	0.051	0.778	N.A.	0.237	0.039	0.039	0.27	-0.03	0.073	-0.32	-0.03	0.051
BM17B	15	0.213	-0.14	0.063	0.102	-0.16	0.328	-0.07	N.A.	-0.05	0.61	1	0.93	0.855	N.A.	-0.12	0.138	0.151	-0.5	0.827	0.392	0.257	-0.11	0.182	0.514	0.097	0.102	1	-0.02	0.717	0.828	1	1	0.647	-0.26	0.138	0.792	-0.25	-0.2	-0.29	0.12	-0.54	0.361	-0.06	-0.18	-1	0.183	-0.06	-0.02	N.A.	N.A.	0	N.A.	N.A.	0.315
BM201B	16	N.A.	N.A.	N.A.	N.A.	N.A.	N.A.	N.A.	N.A.	N.A.	0.815	0.631	0.779	0.675	0.361	N.A.	N.A.	N.A.	N.A.	0.881	N.A.	N.A.	N.A.	1	0.367	0	1	0.663	N.A.	0.906	0.787	0.724	0.839	0.923	N.A.	N.A.	0.786	N.A.	N.A.	N.A.	N.A.	N.A.	N.A.	N.A.	N.A.	N.A.	N.A.	N.A.	N.A.	N.A.	N.A.	N.A.	N.A.	N.A.	0.723
BM201C	17	-0.02	0	-0.05	0.361	-0.07	0	0.254	-0.05	0.656	N.A.	-0.04	N.A.	N.A.	N.A.	0	-0.08	-0.02	-0.2	0	-0.05	-0.02	0.273	N.A.	N.A.	0.659	-0.04	-0.02	1	0.363	0	0	N.A.	0	-0.22	-0.13	N.A.	0.477	0.163	-0.09	0.371	N.A.	-0.06	0	0	N.A.	N.A.	-0.23	0.045	0.154	0.059	-0.04	-0.16	0.237	0.090
BM202A	18	N.A.	N.A.	N.A.	N.A.	N.A.	N.A.	N.A.	N.A.	N.A.	0.226	0.845	0.747	0.764	N.A.	N.A.	N.A.	N.A.	N.A.	0.458	0	-0.04	N.A.	1	0.714	1	1	0.798	N.A.	1	0.761	0.262	0.471	0.584	N.A.	N.A.	0.806	N.A.	N.A.	N.A.	N.A.	N.A.	N.A.	N.A.	N.A.	N.A.	N.A.	N.A.	N.A.	N.A.	N.A.	N.A.	N.A.	N.A.	0.682
BM202B	19	N.A.	N.A.	N.A.	N.A.	N.A.	N.A.	N.A.	N.A.	N.A.	0.136	0.748	0.613	0.644	N.A.	N.A.	N.A.	N.A.	N.A.	0.184	0	-0.02	N.A.	0.862	0.716	1	1	0.644	N.A.	1	-0.04	-0.13	-0.06	0.584	N.A.	N.A.	0.732	N.A.	N.A.	N.A.	N.A.	N.A.	N.A.	N.A.	N.A.	N.A.	N.A.	N.A.	N.A.	N.A.	N.A.	N.A.	N.A.	N.A.	0.543
BM203B	20	0	0	-0.02	0.434	-0.02	-0.04	0.258	-0.05	0.656	N.A.	-0.11	N.A.	N.A.	0	N.A.	-0.08	-0.02	-0.2	0	-0.04	-0.02	0.476	N.A.	N.A.	0.659	0	0	1	-0.02	-0.02	0	N.A.	0	-0.12	-0.13	N.A.	0.659	0.143	-0.05	0.432	N.A.	-0.08	0	N.A.	N.A.	N.A.	-0.1	0.161	0.311	0.186	-0.1	0.076	0.237	0.146
BM203C	21	0.562	0.311	0.199	0.282	0.288	0.425	0.243	0.598	0.025	-0.08	0	1	0.912	N.A.	0.684	0.644	0.527	0	-0.14	0.299	0.372	0.495	0.673	1	0.457	0.004	0.478	0.512	0.656	-0.04	-0.02	-0.02	0.517	0.423	0.633	0.798	0.833	0.184	0.323	0.422	0.617	0.638	0.403	0.229	-0.5	0.27	0.127	0.291	0.275	-0.13	N.A.	0	N.A.	0.415
BM203D	22	N.A.	N.A.	N.A.	N.A.	N.A.	N.A.	N.A.	N.A.	N.A.	0.136	0.748	0.62	0.644	N.A.	N.A.	N.A.	N.A.	N.A.	0.184	0	-0.04	N.A.	0.862	0.841	1	1	0.716	N.A.	1	0.27	-0.13	-0.08	0.584	N.A.	N.A.	0.732	N.A.	N.A.	N.A.	N.A.	N.A.	N.A.	N.A.	N.A.	N.A.	N.A.	N.A.	N.A.	N.A.	N.A.	N.A.	N.A.	N.A.	0.543
BM204A	23	-0.05	-0.07	0	0	0	-0.02	-0.02	-0.05	0.47	N.A.	0	0	-0.04	N.A.	-0.04	-0.19	-0.16	N.A.	0	0.36	0	-0.02	N.A.	0	0	N.A.	N.A.	0	0	N.A.	N.A.	0	0	0.443	-0.22	-0.02	-0.04	-0.17	-0.16	-0.26	N.A.	-0.04	0	N.A.	0	-0.02	0.117	-0.45	-0.23	0.085	0.216	0.016	-0.52	-0.071
BM21B	24	-0.07	-0.04	-0.09	-0.04	-0.08	-0.18	0.25	-0.02	-0.02	0	0	-0.02	0.183	N.A.	-0.2	-0.15	0.012	0.571	0.063	-0.22	0.102	0.788	0.438	0.716	0.151	0.216	0.679	0.378	0.215	-0.28	0.016	0.262	0.199	N.A.	N.A.	0.685	N.A.	N.A.	N.A.	N.A.	N.A.	N.A.	0.357	N.A.	N.A.	N.A.	N.A.	N.A.	N.A.	N.A.	N.A.	N.A.	N.A.	0.157
BM21C	25	-0.07	-0.04	-0.07	-0.04	-0.08	-0.16	0.258	-0.02	-0.02	0	0	-0.02	0.231	N.A.	-0.2	-0.12	0.012	0.294	0.107	-0.23	0.11	0.157	0.345	0.716	0.082	0.164	0.679	0.378	0.253	-0.43	0.01	0.299	0.085	0.416	-0.02	0.685	-0.02	0.251	0.634	-0.12	N.A.	-0.03	0.063	-0.11	N.A.	-0.04	0.157	0.286	-0.12	-0.04	0.337	-0.23	0.015	0.129
BM26B	26	N.A.	N.A.	N.A.	N.A.	0	0	-0.02	N.A.	-0.02	-0.1	0.644	0.403	0.644	N.A.	N.A.	-0.02	N.A.	N.A.	-0.05	-0.05	0.659	-0.06	1	0.716	0.477	0.36	0.718	-0.02	0.675	0.346	0.35	-0.18	0.405	N.A.	0	0.53	N.A.	-0.04	N.A.	-0.02	N.A.	N.A.	0.205	N.A.	N.A.	N.A.	-0.06	1	N.A.	-0.04	-0.27	0.057	-0.02	0.340
BM2G	27	N.A.	N.A.	N.A.	N.A.	N.A.	N.A.	N.A.	N.A.	N.A.	-0.03	0.756	0.359	0.644	N.A.	N.A.	N.A.	N.A.	N.A.	0.305	N.A.	0	N.A.	0.862	0.612	0.659	1	0.714	N.A.	1	0.316	0.076	0.385	-0.15	N.A.	N.A.	0.597	N.A.	N.A.	N.A.	N.A.	N.A.	N.A.	N.A.	N.A.	N.A.	N.A.	N.A.	N.A.	N.A.	N.A.	N.A.	N.A.	N.A.	0.439
BM30A	28	N.A.	N.A.	N.A.	N.A.	N.A.	N.A.	N.A.	N.A.	N.A.	-0.13	0.536	0.445	0.709	0.029	N.A.	N.A.	N.A.	N.A.	0.013	-0.04	-0.04	N.A.	0.86	0.373	1	0.659	0.109	N.A.	0.923	-0.04	-0.21	0.084	-0.34	N.A.	N.A.	0.423	N.A.	N.A.	N.A.	N.A.	N.A.	N.A.	-0.02	N.A.	N.A.	N.A.	N.A.	N.A.	N.A.	N.A.	N.A.	N.A.	N.A.	0.234
BM30C	29	0	N.A.	0	N.A.	N.A.	N.A.	0	0	N.A.	0.084	0.355	0.099	0.208	0.098	N.A.	N.A.	0	N.A.	0.389	0.477	-0.02	-0.02	0.329	0.387	-0.02	-0.02	0.382	N.A.	0.679	-0.05	0.084	-0.12	-0.05	N.A.	N.A.	0.133	N.A.	N.A.	N.A.	N.A.	N.A.	N.A.	N.A.	N.A.	N.A.	0	N.A.	N.A.	N.A.	N.A.	N.A.	N.A.	N.A.	0.176
BM32A	30	0.547	-0.18	-0.16	0.291	0.294	0.27	0.098	0.455	0.63	0.481	0.257	0.363	0.291	0	0.2	0.322	-0.09	0.143	0.567	0.012	0.138	0.344	0.575	0.184	0.282	0.208	0.614	0.44	0.268	0.128	0.202	0.336	-0.02	0.224	0.305	0.404	0.081	0.563	0.098	0.088	-0.05	0.013	0.416	0.158	-0.2	0.348	0.665	0.545	0.067	0.481	0.421	0.391	0.397	0.286
BM33B	31	N.A.	N.A.	N.A.	N.A.	N.A.	N.A.	0	N.A.	N.A.	-0.17	0.536	0.494	0.668	0.272	N.A.	N.A.	N.A.	N.A.	0.622	N.A.	N.A.	N.A.	0.653	0.612	-0.02	N.A.	0.425	N.A.	0.62	0.102	0.655	0.384	-0.03	N.A.	N.A.	0.415	N.A.	N.A.	N.A.	N.A.	N.A.	N.A.	0	N.A.	N.A.	N.A.	N.A.	N.A.	N.A.	N.A.	N.A.	N.A.	N.A.	0.380
BM35C	32	0.055	0.149	0.2	0.183	-0.01	-0.09	0.2	-0.12	0.109	0.368	0.355	0.106	0.085	N.A.	-0.05	-0.09	-0.21	-0.2	0.227	-0.19	0.198	0.117	-0.33	0.127	0.21	-0.23	0.479	-0.15	0.359	0.223	0	0.138	0.405	-0.03	-0.09	-0.02	0.079	-0.13	0.065	0.161	0.138	0.316	-0.44	-0.17	0.5	0.141	0.065	0.216	-0.09	-0.15	-0.36	0.322	-0.12	0.053
BM35D	33	N.A.	N.A.	N.A.	0	N.A.	N.A.	N.A.	N.A.	N.A.	0.511	0.616	0.47	0.231	0.413	N.A.	N.A.	N.A.	N.A.	0.722	N.A.	1	0	1	0.313	0	1	0.278	N.A.	0.913	0.36	0.378	0.415	0.35	N.A.	0	0.581	N.A.	N.A.	N.A.	N.A.	0.429	N.A.	N.A.	N.A.	N.A.	N.A.	0.357	1	-0.02	-0.02	0.159	0.379	0.255	0.419
BM36F	34	0.279	-0.04	-0.07	-0.05	-0.02	-0.04	0	-0.02	-0.05	0.06	0.756	0.197	0.644	N.A.	-0.07	-0.07	-0.06	0	0.043	-0.14	-0.1	-0.14	0.422	0.521	0.527	0.27	0.457	-0.02	0.928	0.102	0.345	-0.17	-0.12	0.072	-0.04	0.585	-0.06	-0.04	-0.06	-0.06	-0.05	-0.07	0	-0.1	N.A.	-0.08	0.198	-0.04	-0.06	-0.02	-0.06	-0.09	-0.1	0.203
BM38B	35	N.A.	-0.02	N.A.	0	0	-0.02	N.A.	0	0	0.133	0.664	0.045	0.133	-0.02	N.A.	N.A.	0	N.A.	0.051	-0.05	0.097	-0.02	0.581	0.188	0.36	-0.02	0.287	-0.06	0.769	0.325	-0.25	-0.13	0.199	0.162	-0.17	0.809	-0.1	-0.04	-0.05	0.328	-0.05	-0.06	0.359	-0.03	N.A.	-0.02	-0.17	-0.18	0.102	0.097	-0.19	0.208	-0.14	0.157
BM44B	36	N.A.	N.A.	N.A.	N.A.	N.A.	N.A.	0	N.A.	N.A.	0.497	1	0.595	1	0.686	N.A.	N.A.	N.A.	N.A.	0.4	0.275	-0.04	N.A.	-0.03	0.843	0	1	0.698	0	0.722	0.169	0.509	-0.02	0.287	N.A.	N.A.	0.905	0	N.A.	N.A.	N.A.	N.A.	N.A.	0.639	0	N.A.	N.A.	N.A.	N.A.	N.A.	N.A.	N.A.	N.A.	N.A.	0.523
BM50B	37	N.A.	N.A.	N.A.	N.A.	N.A.	N.A.	N.A.	N.A.	N.A.	-0.06	0.778	0.364	0	0.268	N.A.	N.A.	N.A.	N.A.	-0.01	N.A.	N.A.	N.A.	0	0.658	N.A.	0	0.789	N.A.	1	0.147	0.208	0.208	-0.04	N.A.	N.A.	0.659	N.A.	N.A.	N.A.	N.A.	N.A.	N.A.	0	N.A.	N.A.	N.A.	N.A.	N.A.	N.A.	N.A.	N.A.	N.A.	N.A.	0.345
BM54A	38	N.A.	N.A.	N.A.	N.A.	N.A.	N.A.	0	-0.02	N.A.	0.287	0.87	0.536	0.698	N.A.	N.A.	N.A.	N.A.	N.A.	0.458	-0.02	-0.04	-0.02	0.862	1	1	1	0.819	N.A.	1	0	0.275	0.638	0.748	N.A.	N.A.	0.732	0	0	N.A.	N.A.	N.A.	N.A.	0	N.A.	N.A.	N.A.	N.A.	N.A.	N.A.	N.A.	0	0	0	0.638
BM57A	39	N.A.	N.A.	N.A.	N.A.	N.A.	N.A.	N.A.	N.A.	N.A.	N.A.	N.A.	N.A.	N.A.	N.A.	N.A.	N.A.	N.A.	N.A.	N.A.	0	N.A.	N.A.	N.A.	N.A.	0	N.A.	N.A.	N.A.	N.A.	N.A.	N.A.	N.A.	N.A.	-0.12	0.525	N.A.	0	0.378	0.051	0.307	N.A.	N.A.	N.A.	N.A.	N.A.	-0.02	-0.16	-0.02	-0.13	0.164	0.297	-0.09	-0.09	0.062
BM57D	40	N.A.	N.A.	N.A.	N.A.	N.A.	N.A.	N.A.	N.A.	N.A.	N.A.	N.A.	N.A.	N.A.	N.A.	N.A.	N.A.	N.A.	N.A.	0	0	N.A.	N.A.	0	N.A.	0	N.A.	N.A.	N.A.	N.A.	N.A.	N.A.	N.A.	N.A.	-0.19	0.519	N.A.	0	0.263	0.015	0.199	N.A.	N.A.	N.A.	N.A.	N.A.	0	-0.26	0.24	0.051	-0.02	0.147	-0.09	-0.04	0.044
BM5B	41	N.A.	N.A.	N.A.	N.A.	N.A.	N.A.	N.A.	N.A.	-0.02	-0.07	0	-0.02	0	0	N.A.	N.A.	N.A.	N.A.	N.A.	N.A.	0	N.A.	N.A.	-0.04	N.A.	0	-0.02	N.A.	N.A.	0	-0.02	N.A.	-0.02	N.A.	N.A.	0	N.A.	0	N.A.	N.A.	N.A.	N.A.	N.A.	N.A.	N.A.	0	N.A.	-0.02	N.A.	N.A.	0	-0.09	-0.16	-0.057
BM5D	42	0.046	-0.02	0.277	0.169	-0.31	-0.05	-0.22	-0.08	-0.26	-0.28	-0.23	0.251	-0.06	-0.16	-0.2	0.102	-0.18	0	-0.33	-0.23	-0.18	0.103	0.208	0.161	0.414	0.266	0.195	-0.13	-0.13	-0.13	0.183	-0.04	-0.36	-0.2	0.022	0.104	0.121	-0.01	0.466	-0.07	-0.13	-0	0.088	-0.39	-0.2	0.016	0.125	0.015	0.164	-0.22	-0.03	-0.16	-0.16	-0.014
BM60A	43	N.A.	N.A.	N.A.	N.A.	N.A.	0	N.A.	N.A.	-0.03	0.108	-0.1	0.109	0.64	0.204	0	N.A.	N.A.	N.A.	0.085	-0.04	0	N.A.	0.329	0.107	0	-0.02	0.195	N.A.	-0.05	-0.09	-0.15	-0.05	0.028	N.A.	N.A.	0.62	N.A.	N.A.	-0.02	N.A.	N.A.	N.A.	-0.06	-0.03	N.A.	0	N.A.	N.A.	0	N.A.	N.A.	N.A.	N.A.	0.095
BM61A	44	0.149	0.321	0.032	0.016	0.103	-0.05	0.148	0.199	-0.25	0.256	0.059	0.099	0.266	N.A.	-0.28	0.208	-0.03	-0.5	0.132	-0.04	-0.13	-0.19	0.227	0.188	0.243	0.216	0.455	0.63	0.196	0.305	-0.09	0.019	0.059	0.089	0.008	-0.24	0.322	0.028	-0.01	0.455	-0.11	0.101	-0.23	-0.19	-0.2	0.526	0.308	-0.11	-0.04	-0.23	-0.06	-0.17	-0.1	0.084
BM62A	45	-0.03	0.659	0.659	-0.05	-0.02	0.151	0.328	0.047	-0.05	0.483	0.559	0.251	0.474	N.A.	0.28	0.526	0.322	N.A.	0.616	0.36	0.453	0.477	1	0.841	0.358	0.659	0.464	1	0.866	-0.12	0.659	-0.04	0.449	N.A.	N.A.	0.747	N.A.	N.A.	N.A.	N.A.	N.A.	N.A.	0.477	N.A.	N.A.	N.A.	N.A.	N.A.	N.A.	N.A.	N.A.	N.A.	N.A.	0.436
BM64A	46	N.A.	N.A.	N.A.	N.A.	N.A.	N.A.	N.A.	N.A.	N.A.	0.064	0.756	0.359	0.644	N.A.	N.A.	N.A.	N.A.	N.A.	0.327	0.273	-0.1	N.A.	0.862	0.841	0.659	1	0.575	N.A.	0.923	0.202	-0.18	0.275	-0.06	N.A.	0	0.685	N.A.	N.A.	N.A.	N.A.	N.A.	N.A.	-0.04	N.A.	N.A.	N.A.	N.A.	N.A.	0	N.A.	N.A.	N.A.	N.A.	0.395
BM67C	47	0.362	0.208	0.768	0.457	0.53	0.639	0.656	0.049	0.628	0.385	0.536	0.641	0.799	N.A.	0.517	0.891	1	0.571	0.44	-0.18	0.444	0.638	0.566	1	0.496	0.72	0.893	0.795	0.812	0.638	0	0.201	0.432	0.844	0.636	0.681	0.453	0.563	0.855	0.437	0.429	0.415	0.671	0.455	0.571	0.449	0.462	0.216	0.305	0.367	-0.09	0.231	-0.09	0.524
BM6C	48	-0.09	0.298	-0.08	0.117	0.055	0.277	-0.08	0.152	-0.07	0.222	0.125	-0.09	-0.01	-0.06	-0.15	-0.15	-0.18	N.A.	-0.07	0.361	-0.25	-0.25	-0.05	0.224	-0.05	0.016	-0.02	-0.08	-0.05	0.125	0.016	-0.09	0.013	0	-0.08	-0.02	0.101	-0.06	-0.06	-0.06	-0.05	-0.08	0.169	N.A.	N.A.	-0.07	N.A.	0	N.A.	N.A.	N.A.	N.A.	N.A.	-0.004
BM75C	49	N.A.	N.A.	N.A.	N.A.	N.A.	N.A.	N.A.	N.A.	N.A.	-0.06	N.A.	N.A.	N.A.	0	N.A.	N.A.	N.A.	N.A.	0.31	0	0.658	N.A.	N.A.	0.429	N.A.	1	0.477	N.A.	0	0.291	0.066	0.316	0.161	N.A.	N.A.	0.659	N.A.	N.A.	N.A.	N.A.	N.A.	N.A.	N.A.	N.A.	N.A.	N.A.	N.A.	N.A.	N.A.	N.A.	N.A.	N.A.	N.A.	0.283
BM78B	50	0.659	N.A.	N.A.	1	1	-0.04	N.A.	N.A.	0	-0.01	N.A.	0	N.A.	N.A.	N.A.	-0.02	0	-0.2	0.283	-0.2	-0.15	-0.04	0	1	0.097	-0.04	0	0	-0.02	0	0	1	0.658	0.125	0	0.275	-0.08	0.316	-0.02	0.225	-0.06	0.091	-0.14	-0.19	-0.2	0	0.155	0.216	0.211	-0.05	-0.23	0.016	0.015	0.100
BM8E	51	0	0	-0.03	N.A.	0	N.A.	0	0	-0.02	-0.11	0.644	0.412	0.519	N.A.	-0.02	N.A.	-0.04	N.A.	0.41	-0.07	0.143	-0.04	0.862	0.841	0.659	0.477	0.571	0	0.928	-0.05	0.067	-0.18	-0.13	N.A.	N.A.	0.8	0	0	N.A.	N.A.	N.A.	0	N.A.	0	N.A.	N.A.	0	N.A.	N.A.	N.A.	0	0.477	N.A.	0.319
BM92B	52	N.A.	N.A.	N.A.	N.A.	N.A.	N.A.	N.A.	N.A.	N.A.	-0.12	0.644	0.403	0.776	N.A.	N.A.	N.A.	N.A.	N.A.	0.095	-0.07	0.477	0	0.862	1	1	1	0.716	N.A.	1	0.231	-0.16	0.057	0.036	N.A.	N.A.	0.747	N.A.	N.A.	N.A.	N.A.	N.A.	N.A.	N.A.	N.A.	N.A.	N.A.	N.A.	N.A.	N.A.	N.A.	N.A.	N.A.	N.A.	0.460
BM9B	53	0.345	0.287	0.183	0.391	0.494	0.056	0.214	0.275	-0.31	0.284	0.732	0.101	0.294	0.216	0.381	0.018	0.051	-0.2	0.714	0.206	0.368	-0.25	0.277	0.695	0.537	0.155	0.269	0.034	0.216	0.712	0.415	0.684	0.47	-0.12	0.206	0.731	0.456	-0.02	0.213	-0.11	0.31	0.137	0.047	0.355	0.143	-0.01	-0.11	-0.03	0	0.156	-0.12	-0.07	-0.02	0.234
BM9C	54	0.275	0.155	0.281	0.362	0.444	0.101	0.466	-0.02	-0.14	-0.07	0.678	0.012	0.369	-0.04	0.365	0.122	0.097	-0.5	0.218	0.048	0.243	-0.14	0.422	0.209	0.44	0.16	0.303	-0.09	0.356	-0.07	-0.14	-0.22	0.193	0.059	0.167	0.255	0.474	0.028	0.213	-0.05	0.394	0.075	0.092	0.316	0.333	0.01	-0.09	-0.06	-0.03	0.156	-0.16	0.042	-0	0.155

Supplementary Table S5. FST distance matrix for 54 SNP. P<0.05 after FDR-BY correction is marked in bold. See Table 1 for site name definition.

		BNJ	IRD	KPV	MID	PNY	SNY	NWF	PEI	SNS	KKA	PBAY	GLD	GLL	NUU	ISLB	ISLR	SPI	BRN	BODS	BODZ	TRO	BAR	KOL	DLZ	CHU	KER	ONE	WSBS	BIA	BOR	KLA	STC	SAL	CIS	SCO	LET	OBA	IONA	KRR	STA	LGF	GBLO	NLOO	LOI	MSM	BID	VIG	CAS	CAM	IMC	NEA	AZO
1	BNJ																																																				
2	IRD	0.004																																																			
3	KPV	0.003	0.009																																																		
4	MID	0.005	0.012	0.010																																																	
5	PNY	-0.006	0.003	0.000	-0.002																																																
6	SNY	0.004	0.014	-0.004	0.011	-0.002																																															
7	NWF	**0.040**	**0.050**	**0.020**	**0.051**	**0.042**	0.024																																														
8	PEI	**0.036**	**0.035**	**0.025**	**0.047**	**0.036**	**0.027**	0.009																																													
9	SNS	0.003	0.014	0.011	0.011	0.006	0.004	0.014	0.005																																												
10	KKA	**0.659**	**0.654**	**0.662**	**0.658**	**0.665**	**0.655**	**0.647**	**0.654**	**0.641**																																											
11	PBAY	**0.468**	**0.460**	**0.471**	**0.466**	**0.477**	**0.461**	**0.442**	**0.460**	**0.435**	**0.090**																																										
12	GLD	**0.476**	**0.466**	**0.477**	**0.473**	**0.483**	**0.468**	**0.457**	**0.463**	**0.449**	0.071	-0.006																																									
13	GLL	**0.223**	**0.212**	**0.225**	**0.225**	**0.229**	**0.211**	**0.202**	**0.204**	**0.197**	**0.305**	**0.078**	0.100																																								
14	LBG1	**0.063**	**0.068**	**0.065**	**0.076**	**0.066**	**0.040**	**0.036**	**0.027**	**0.032**	**0.641**	**0.439**	**0.447**	**0.183**																																							
15	ISLB	**0.155**	**0.177**	**0.146**	**0.174**	**0.154**	**0.114**	**0.124**	**0.168**	**0.149**	**0.662**	**0.478**	**0.490**	**0.245**	**0.126**																																						
16	ISLR	**0.128**	**0.160**	**0.131**	**0.162**	**0.128**	**0.093**	**0.113**	**0.144**	**0.126**	**0.665**	**0.482**	**0.492**	**0.242**	**0.098**	0.002																																					
17	SPI	**0.160**	**0.159**	**0.161**	**0.158**	**0.149**	**0.104**	**0.199**	**0.221**	**0.166**	**0.608**	**0.349**	**0.395**	**0.159**	**0.167**	0.067	**0.092**																																				
18	BRN	**0.415**	**0.410**	**0.417**	**0.418**	**0.420**	**0.406**	**0.388**	**0.404**	**0.387**	**0.148**	0.041	**0.057**	**0.101**	**0.384**	**0.397**	**0.400**	**0.308**																																			
19	BODS	**0.166**	**0.181**	**0.159**	**0.187**	**0.156**	**0.129**	**0.153**	**0.190**	**0.162**	**0.623**	**0.425**	**0.447**	**0.217**	**0.161**	**0.024**	**0.032**	0.035	**0.345**																																		
20	BODZ	**0.137**	**0.145**	**0.138**	**0.158**	**0.129**	**0.103**	**0.126**	**0.152**	**0.129**	**0.593**	**0.383**	**0.409**	**0.169**	**0.118**	**0.033**	**0.027**	0.028	**0.314**	0.006																																	
21	TRO	**0.205**	**0.233**	**0.206**	**0.230**	**0.200**	**0.174**	**0.190**	**0.239**	**0.210**	**0.661**	**0.481**	**0.496**	**0.266**	**0.203**	0.018	**0.032**	**0.101**	**0.389**	0.006	**0.027**																																
22	BAR	**0.209**	**0.205**	**0.205**	**0.219**	**0.208**	**0.177**	**0.179**	**0.205**	**0.186**	**0.429**	0.183	**0.222**	0.041	**0.166**	**0.133**	**0.139**	0.076	**0.150**	**0.092**	0.054	**0.132**																															
23	KOL	**0.651**	**0.646**	**0.655**	**0.650**	**0.658**	**0.646**	**0.638**	**0.650**	**0.631**	-0.003	0.074	0.064	**0.279**	**0.631**	**0.647**	**0.651**	**0.570**	**0.124**	**0.602**	**0.569**	**0.644**	**0.388**																														
24	DLZ	**0.186**	**0.193**	**0.192**	**0.203**	**0.179**	**0.152**	**0.179**	**0.210**	**0.181**	**0.622**	**0.426**	**0.445**	**0.207**	**0.164**	**0.029**	**0.041**	0.033	**0.342**	0.007	0.005	0.017	0.083	**0.601**																													
25	CHU	**0.594**	**0.586**	**0.597**	**0.593**	**0.600**	**0.587**	**0.579**	**0.590**	**0.573**	0.013	0.033	0.024	**0.215**	**0.572**	**0.592**	**0.596**	**0.510**	**0.081**	**0.547**	**0.511**	**0.591**	**0.325**	0.000	**0.543**																												
26	KER	**0.171**	**0.167**	**0.170**	**0.178**	**0.171**	**0.153**	**0.147**	**0.166**	**0.152**	**0.387**	**0.152**	**0.193**	0.030	**0.146**	**0.130**	**0.134**	0.075	**0.127**	**0.093**	**0.065**	**0.128**	-0.007	**0.351**	**0.085**	**0.291**																											
27	ONE	**0.167**	**0.190**	**0.176**	**0.196**	**0.161**	**0.141**	**0.174**	**0.197**	**0.167**	**0.660**	**0.477**	**0.490**	**0.252**	**0.168**	**0.028**	0.023	0.076	**0.389**	**0.015**	**0.023**	0.016	**0.140**	**0.645**	0.016	**0.589**	**0.123**																										
28	WSBS	**0.149**	**0.176**	**0.148**	**0.177**	**0.149**	**0.114**	**0.145**	**0.179**	**0.156**	**0.637**	**0.445**	**0.461**	**0.221**	**0.143**	0.007	**0.010**	0.044	**0.366**	0.015	0.019	0.017	**0.105**	**0.619**	0.017	**0.563**	**0.103**	0.009																									
29	BIA	**0.581**	**0.578**	**0.588**	**0.585**	**0.590**	**0.577**	**0.559**	**0.574**	**0.555**	**0.184**	**0.146**	**0.161**	**0.248**	**0.556**	**0.556**	**0.561**	**0.482**	**0.073**	**0.507**	**0.473**	**0.545**	**0.307**	**0.164**	**0.498**	**0.141**	**0.272**	**0.545**	**0.526**																								
30	BOR	**0.579**	**0.575**	**0.585**	**0.581**	**0.588**	**0.575**	**0.555**	**0.569**	**0.550**	**0.150**	**0.130**	**0.133**	**0.243**	**0.551**	**0.560**	**0.565**	**0.485**	**0.074**	**0.515**	**0.482**	**0.553**	**0.313**	**0.143**	**0.507**	**0.122**	**0.282**	**0.551**	**0.531**	0.007																							
31	KLA	**0.593**	**0.591**	**0.599**	**0.597**	**0.602**	**0.588**	**0.569**	**0.585**	**0.566**	**0.174**	**0.150**	**0.158**	**0.262**	**0.567**	**0.570**	**0.575**	**0.500**	**0.077**	**0.523**	**0.491**	**0.562**	**0.328**	**0.161**	**0.517**	**0.142**	**0.289**	**0.559**	**0.539**	-0.002	0.005																						
32	STC	**0.513**	**0.510**	**0.519**	**0.517**	**0.522**	**0.506**	**0.487**	**0.507**	**0.482**	**0.187**	**0.114**	**0.125**	**0.183**	**0.482**	**0.481**	**0.487**	**0.389**	0.038	**0.429**	**0.398**	**0.470**	**0.226**	**0.160**	**0.421**	**0.129**	**0.201**	**0.468**	**0.447**	0.014	0.013	0.008																					
33	SAL	**0.208**	**0.230**	**0.215**	**0.235**	**0.203**	**0.176**	**0.209**	**0.241**	**0.206**	**0.651**	**0.469**	**0.481**	**0.251**	**0.211**	**0.044**	**0.050**	0.078	**0.374**	0.003	0.020	0.018	**0.127**	**0.634**	0.006	**0.579**	**0.119**	0.012	0.021	**0.526**	**0.536**	**0.543**	**0.449**																				
34	CIS	**0.244**	**0.268**	**0.245**	**0.255**	**0.234**	**0.203**	**0.257**	**0.295**	**0.249**	**0.667**	**0.495**	**0.508**	**0.293**	**0.252**	**0.092**	**0.108**	0.048	**0.412**	**0.041**	**0.067**	**0.058**	**0.165**	**0.648**	**0.071**	**0.598**	**0.166**	**0.095**	**0.084**	**0.569**	**0.578**	**0.586**	**0.497**	**0.083**																			
35	SCO	**0.202**	**0.231**	**0.205**	**0.222**	**0.198**	**0.166**	**0.207**	**0.242**	**0.202**	**0.662**	**0.482**	**0.499**	**0.279**	**0.205**	**0.062**	**0.070**	0.050	**0.405**	**0.040**	**0.064**	**0.048**	**0.166**	**0.642**	**0.067**	**0.592**	**0.160**	**0.066**	**0.058**	**0.556**	**0.564**	**0.572**	**0.484**	**0.068**	0.009																		
36	LET	**0.360**	**0.354**	**0.362**	**0.365**	**0.365**	**0.347**	**0.338**	**0.354**	**0.333**	**0.185**	0.027	0.053	0.051	**0.332**	**0.339**	**0.340**	**0.241**	0.026	**0.284**	**0.245**	**0.330**	**0.083**	**0.149**	**0.280**	0.102	0.069	**0.326**	**0.304**	**0.127**	**0.131**	**0.138**	0.082	**0.313**	**0.348**	**0.343**																	
37	OBA	**0.269**	**0.297**	**0.275**	**0.293**	**0.267**	**0.231**	**0.273**	**0.315**	**0.278**	**0.682**	**0.516**	**0.525**	**0.302**	**0.266**	**0.042**	**0.062**	**0.114**	**0.417**	**0.033**	**0.044**	0.011	0.158	**0.665**	0.028	**0.613**	0.159	**0.040**	0.030	**0.567**	**0.576**	**0.584**	**0.492**	**0.034**	**0.059**	**0.055**	**0.355**																
38	IONA	**0.227**	**0.242**	**0.221**	**0.231**	**0.220**	**0.187**	**0.223**	**0.260**	**0.221**	**0.652**	**0.468**	**0.489**	**0.278**	**0.226**	**0.117**	**0.139**	0.032	**0.404**	**0.090**	**0.107**	**0.118**	**0.183**	**0.631**	**0.116**	**0.581**	**0.174**	**0.141**	**0.117**	**0.555**	**0.562**	**0.571**	**0.483**	**0.140**	**0.033**	0.019	**0.346**	**0.130**															
39	KRR	**0.214**	**0.226**	**0.214**	**0.230**	**0.204**	**0.173**	**0.233**	**0.255**	**0.222**	**0.663**	**0.487**	**0.497**	**0.271**	**0.212**	**0.071**	**0.084**	0.032	**0.405**	**0.047**	**0.060**	**0.063**	**0.156**	**0.646**	**0.059**	**0.592**	**0.155**	**0.070**	**0.061**	**0.561**	**0.568**	**0.578**	**0.486**	**0.083**	**0.025**	0.007	**0.343**	**0.065**	0.032														
40	STA	**0.257**	**0.274**	**0.256**	**0.265**	**0.254**	**0.220**	**0.258**	**0.293**	**0.254**	**0.660**	**0.481**	**0.502**	**0.300**	**0.259**	**0.171**	**0.185**	0.084	**0.422**	**0.143**	**0.148**	**0.174**	**0.214**	**0.640**	**0.172**	**0.591**	**0.203**	**0.193**	**0.167**	**0.571**	**0.577**	**0.585**	**0.503**	**0.200**	**0.067**	**0.046**	**0.363**	**0.185**	-0.001	**0.064**													
41	LGF	**0.280**	**0.300**	**0.288**	**0.298**	**0.271**	**0.243**	**0.295**	**0.326**	**0.282**	**0.680**	**0.514**	**0.521**	**0.303**	**0.282**	**0.075**	**0.095**	**0.087**	**0.413**	**0.028**	**0.051**	**0.027**	**0.166**	**0.664**	0.028	**0.611**	**0.160**	**0.040**	**0.062**	**0.565**	**0.574**	**0.584**	**0.489**	**0.028**	**0.045**	**0.056**	**0.350**	0.013	**0.127**	**0.061**	**0.188**												
42	GBLO	**0.288**	**0.316**	**0.290**	**0.317**	**0.287**	**0.244**	**0.298**	**0.341**	**0.302**	**0.661**	**0.463**	**0.484**	**0.266**	**0.286**	**0.048**	**0.069**	**0.109**	**0.372**	0.009	0.041	0.008	0.130	**0.635**	0.028	**0.581**	0.133	**0.042**	0.027	**0.530**	**0.539**	**0.548**	**0.445**	**0.021**	**0.049**	**0.050**	**0.310**	-0.007	**0.125**	**0.063**	**0.185**	0.004											
43	NLOO	**0.293**	**0.319**	**0.294**	**0.319**	**0.283**	**0.248**	**0.301**	**0.335**	**0.297**	**0.663**	**0.479**	**0.491**	**0.272**	**0.289**	**0.074**	**0.082**	**0.120**	**0.376**	0.006	**0.035**	0.020	**0.134**	**0.643**	0.034	**0.587**	**0.138**	**0.046**	**0.050**	**0.538**	**0.547**	**0.556**	**0.457**	**0.017**	**0.050**	**0.060**	**0.313**	0.025	**0.139**	**0.074**	**0.198**	0.005	**-0.010**										
44	LOI	**0.295**	**0.321**	**0.300**	**0.321**	**0.290**	**0.255**	**0.307**	**0.342**	**0.299**	**0.685**	**0.524**	**0.529**	**0.314**	**0.296**	**0.088**	**0.099**	**0.112**	**0.418**	**0.024**	**0.056**	**0.034**	**0.169**	**0.669**	**0.042**	**0.617**	**0.171**	**0.066**	**0.067**	**0.574**	**0.583**	**0.591**	**0.497**	**0.032**	**0.041**	**0.061**	**0.357**	0.018	**0.129**	**0.075**	**0.191**	0.004	**-0.009**	-0.005									
45	MSM	**0.291**	**0.306**	**0.280**	**0.307**	**0.286**	**0.232**	**0.298**	**0.341**	**0.293**	**0.639**	**0.405**	**0.446**	0.222	**0.280**	0.036	**0.080**	0.043	**0.336**	-0.008	0.024	0.028	0.087	0.603	0.019	**0.549**	0.101	0.063	0.031	**0.500**	**0.508**	**0.519**	**0.407**	0.022	0.028	0.039	0.271	0.010	**0.087**	0.044	**0.158**	0.003	-0.043	0.000	-0.014								
46	BID	**0.438**	**0.449**	**0.436**	**0.428**	**0.439**	**0.412**	**0.433**	**0.464**	**0.423**	**0.679**	**0.526**	**0.544**	**0.397**	**0.439**	**0.369**	**0.401**	**0.267**	**0.476**	**0.331**	**0.338**	**0.374**	**0.349**	**0.657**	**0.362**	**0.619**	**0.325**	**0.403**	**0.373**	**0.605**	**0.608**	**0.618**	**0.544**	**0.391**	**0.240**	**0.247**	**0.432**	**0.383**	**0.148**	**0.277**	**0.134**	**0.373**	**0.377**	**0.393**	**0.377**	**0.321**							
47	VIG	**0.460**	**0.467**	**0.460**	**0.452**	**0.461**	**0.438**	**0.454**	**0.484**	**0.444**	**0.689**	**0.540**	**0.560**	**0.417**	**0.460**	**0.401**	**0.429**	**0.304**	**0.491**	**0.358**	**0.365**	**0.401**	**0.374**	**0.669**	**0.387**	**0.631**	**0.346**	**0.428**	**0.404**	**0.614**	**0.619**	**0.628**	**0.556**	**0.421**	**0.271**	**0.274**	**0.450**	**0.413**	**0.171**	**0.299**	**0.147**	**0.402**	**0.409**	**0.426**	**0.408**	**0.360**	0.008						
48	CAS	**0.475**	**0.488**	**0.477**	**0.467**	**0.477**	**0.452**	**0.473**	**0.503**	**0.462**	**0.694**	**0.549**	**0.567**	**0.427**	**0.479**	**0.415**	**0.441**	**0.315**	**0.499**	**0.372**	**0.378**	**0.416**	**0.387**	**0.675**	**0.403**	**0.638**	**0.357**	**0.444**	**0.415**	**0.623**	**0.627**	**0.636**	**0.564**	**0.434**	**0.280**	**0.286**	**0.457**	**0.426**	**0.181**	**0.320**	**0.159**	**0.417**	**0.426**	**0.440**	**0.420**	**0.379**	-0.001	-0.001					
49	CAM	**0.457**	**0.468**	**0.456**	**0.450**	**0.457**	**0.432**	**0.454**	**0.485**	**0.447**	**0.690**	**0.541**	**0.558**	**0.410**	**0.460**	**0.397**	**0.423**	**0.298**	**0.489**	**0.352**	**0.354**	**0.397**	**0.366**	**0.671**	**0.385**	**0.631**	**0.339**	**0.425**	**0.395**	**0.616**	**0.622**	**0.631**	**0.557**	**0.417**	**0.258**	**0.266**	**0.445**	**0.408**	**0.158**	**0.292**	**0.131**	**0.401**	**0.407**	**0.423**	**0.404**	**0.362**	0.003	0.001	0.004				
50	IMC	**0.554**	**0.561**	**0.555**	**0.545**	**0.557**	**0.535**	**0.550**	**0.573**	**0.537**	**0.698**	**0.553**	**0.571**	**0.449**	**0.549**	**0.492**	**0.523**	**0.396**	**0.502**	**0.441**	**0.443**	**0.495**	**0.425**	**0.677**	**0.465**	**0.640**	**0.386**	**0.517**	**0.485**	**0.622**	**0.624**	**0.635**	**0.562**	**0.504**	**0.380**	**0.375**	**0.467**	**0.508**	**0.265**	**0.403**	**0.255**	**0.498**	**0.510**	**0.527**	**0.503**	**0.457**	**0.046**	**0.036**	**0.044**	**0.065**			
51	NEA	**0.539**	**0.546**	**0.539**	**0.530**	**0.542**	**0.520**	**0.534**	**0.558**	**0.523**	**0.704**	**0.571**	**0.586**	**0.464**	**0.536**	**0.480**	**0.510**	**0.385**	**0.521**	**0.439**	**0.443**	**0.484**	**0.437**	**0.686**	**0.462**	**0.651**	**0.401**	**0.505**	**0.479**	**0.636**	**0.638**	**0.648**	**0.579**	**0.497**	**0.369**	**0.368**	**0.486**	**0.492**	**0.259**	**0.393**	**0.247**	**0.482**	**0.493**	**0.510**	**0.488**	**0.446**	**0.037**	**0.028**	**0.029**	**0.056**	-0.008		
52	AZO	**0.562**	**0.570**	**0.563**	**0.552**	**0.566**	**0.545**	**0.562**	**0.583**	**0.551**	**0.717**	**0.591**	**0.600**	**0.482**	**0.565**	**0.503**	**0.535**	**0.430**	**0.534**	**0.461**	**0.467**	**0.508**	**0.461**	**0.701**	**0.486**	**0.666**	**0.417**	**0.532**	**0.504**	**0.650**	**0.652**	**0.662**	**0.594**	**0.520**	**0.396**	**0.393**	**0.502**	**0.520**	**0.291**	**0.424**	**0.288**	**0.508**	**0.529**	**0.543**	**0.515**	**0.484**	**0.077**	**0.072**	**0.059**	**0.117**	0.034	**0.035**	
53	**SAV**	**0.804**	**0.8**	**0.814**	**0.806**	**0.813**	**0.81**	**0.81**	**0.82**	**0.81**	**0.047**	**0.265**	**0.21**	**0.47**	**0.81**	**0.81**	**0.81**	**0.8**	**0.3**	**0.763**	**0.73**	**0.8**	**0.598**	**0.06**	**0.76**	**0.1**	**0.53**	**0.81**	**0.78**	**0.333**	**0.29**	**0.31**	**0.34**	**0.8**	**0.8**	**0.8**	**0.33**	**0.819**	**0.785**	**0.8**	**0.79**	**0.82**	**0.829**	**0.824**	**0.821**	**0.83**	**0.791**	**0.798**	**0.81**	**0.8**	**0.813**	**0.8**	**0.82**

(shaded) excluded from NJ tree

Supplementary Table S6. The explained variability of the studied environmental variables in different allele models of *Mytilus* spp. Variables that explained over 10% of variability in a model are highlighted in green.

Allele	Chlorophyll	Cloudiness	Icecover	Nitrate	Phosphate	Precipitation	Region	Salinity	SolarRadiation	Temperature	TidalRange	WaveHeight	WindSpeed
BM101A	0	4	1	0	2	6	4	**38**	2	**12**	7	0	3
BM102A	0	0	0	0	0	0	0	0	0	0	0	0	0
BM103B	5	0	5	1	0	2	6	3	0	4	1	1	1
BM105A	0	0	0	0	1	0	0	1	0	0	0	0	0
BM106B	0	0	0	0	0	0	0	0	0	0	0	0	0
BM10B	3	0	5	1	0	2	3	3	0	2	1	1	1
BM113A	**10**	0	**10**	2	0	6	**15**	2	2	7	1	1	2
BM118A	0	0	0	0	0	0	0	0	0	0	0	0	0
BM11A	9	0	**10**	2	1	4	**12**	4	1	7	1	2	2
BM121A	0	0	0	0	0	0	0	0	0	0	0	0	0
BM12A	8	0	7	2	1	4	**10**	5	1	4	2	1	2
BM12C	2	0	1	1	1	2	2	1	1	1	2	1	1
BM151A	0	0	0	0	0	0	0	0	0	0	0	0	0
BM16B	0	0	0	0	0	0	0	0	0	0	0	0	0
BM17B	4	2	2	3	3	7	1	**11**	3	1	4	1	2
BM201B	4	0	5	1	0	2	7	3	0	2	1	1	1
BM201C	1	1	0	2	4	1	3	**10**	4	6	3	1	2
BM202A	**10**	0	**12**	2	0	5	**14**	3	1	5	1	1	2
BM202B	**11**	0	**11**	2	0	5	**16**	3	1	7	1	2	2
BM203B	1	1	0	2	4	1	2	8	4	5	3	1	1
BM203C	1	0	0	0	0	0	0	0	0	0	0	0	0
BM203D	**10**	0	**11**	2	0	5	**16**	3	1	7	1	1	2
BM204A	0	0	0	0	0	0	0	0	0	0	0	0	0
BM21B	5	1	5	5	8	1	1	**24**	4	3	3	2	1
BM21C	4	4	2	**10**	2	5	1	4	6	3	3	2	2
BM26B	8	0	8	3	1	3	9	3	1	6	2	3	2

BM2G	7	0	9	1	0	4	3	3	1	3	1	2	2
BM30A	3	0	5	1	0	3	4	4	0	3	1	2	1
BM30C	1	0	1	0	0	0	1	1	0	1	0	0	0
BM32A	0	0	0	0	0	0	0	0	0	0	0	0	0
BM33B	1	0	3	0	0	1	1	2	0	1	0	1	0
BM35C	2	1	1	2	1	1	0	1	3	2	2	1	1
BM35D	1	0	2	1	0	1	1	1	0	1	1	1	0
BM36F	4	1	6	1	1	3	2	3	1	2	2	1	2
BM38B	2	0	2	1	0	2	1	2	1	1	1	1	1
BM44B	3	0	3	0	0	1	5	3	0	2	1	1	1
BM50B	0	0	0	0	0	0	0	0	0	0	0	0	0
BM54A	**10**	0	**10**	3	0	5	**15**	3	1	8	1	2	2
BM57A	0	1	1	2	5	2	2	**13**	4	4	5	0	1
BM57D	0	1	0	1	3	1	2	6	2	2	2	0	1
BM5B	0	0	0	0	0	0	0	0	0	0	0	0	0
BM5D	1	0	0	0	0	1	1	0	1	1	0	0	0
BM60A	1	0	1	0	0	0	2	2	0	0	1	1	0
BM61A	2	1	1	1	1	2	0	2	1	1	2	1	1
BM62A	7	1	8	2	3	1	7	4	2	**16**	1	1	2
BM64A	7	0	7	1	0	3	5	6	1	5	1	2	2
BM67C	5	1	2	5	2	7	7	1	3	2	3	1	2
BM6C	0	0	0	0	0	0	0	1	0	1	0	0	0
BM75C	0	0	0	0	0	0	0	0	0	0	0	0	0
BM78B	1	1	1	1	3	1	2	6	2	2	4	0	1
BM8E	7	0	7	1	0	2	4	5	1	4	1	2	1
BM92B	**10**	0	**10**	2	0	4	**12**	5	1	8	1	2	2
BM9B	1	1	0	1	1	2	2	3	1	1	1	1	1
BM9C	2	1	2	1	2	1	1	5	2	1	2	0	1
